# Characterization of Noise Signatures of Involuntary Head Motion in the Autism Brain Imaging Data Exchange Repository

**DOI:** 10.3389/fnint.2018.00007

**Published:** 2018-03-05

**Authors:** Carla Caballero, Sejal Mistry, Joe Vero, Elizabeth B Torres

**Affiliations:** ^1^Department of Psychology, Rutgers University, New Brunswick, NJ, United States; ^2^Department of Mathematics, Rutgers University, Piscataway, NJ, United States; ^3^Department of Biomedical Engineering, Rutgers University, New Brunswick, NJ, United States; ^4^Cognitive Science Center, Rutgers University, New Brunswick, NJ, United States; ^5^Computational Biomedicine Imaging and Modeling Center, Rutgers University, New Brunswick, NJ, United States

**Keywords:** autism, Asperger's, noise, stochastic process, head motion, resting-state fMRI

## Abstract

The variability inherently present in biophysical data is partly contributed by disparate sampling resolutions across instrumentations. This poses a potential problem for statistical inference using pooled data in open access repositories. Such repositories combine data collected from multiple research sites using variable sampling resolutions. One example is the Autism Brain Imaging Data Exchange repository containing thousands of imaging and demographic records from participants in the spectrum of autism and age-matched neurotypical controls. Further, statistical analyses of groups from different diagnoses and demographics may be challenging, owing to the disparate number of participants across different clinical subgroups. In this paper, we examine the noise signatures of head motion data extracted from resting state fMRI data harnessed under different sampling resolutions. We characterize the quality of the noise in the variability of the raw linear and angular speeds for different clinical phenotypes in relation to age-matched controls. Further, we use bootstrapping methods to ensure compatible group sizes for statistical comparison and report the ranges of physical involuntary head excursions of these groups. We conclude that different sampling rates do affect the quality of noise in the variability of head motion data and, consequently, the type of random process appropriate to characterize the time series data. Further, given a qualitative range of noise, from pink to brown noise, it is possible to characterize different clinical subtypes and distinguish them in relation to ranges of neurotypical controls. These results may be of relevance to the pre-processing stages of the pipeline of analyses of resting state fMRI data, whereby head motion enters the criteria to clean imaging data from motion artifacts.

## Introduction

The advent of open-access data repositories across various scientific fields has initiated new avenues with the potential for transformative discoveries. While poised for a rapid change in pace across many medical fields, particularly those related to the health and brain sciences, these new initiatives have also started to encourage novel exchange and reproducibility of results across labs worldwide. The field of autism research is among those beginning to greatly benefit from these new databanks. Scientists now have at their disposal the opportunity of uncovering new mechanisms and reporting new correlations in multi-modal data with high statistical power, owing this new possibility to the large number of available participants' data. Indeed, it is now possible to aggregate data from different sites and attain a very large number of subjects to build normative data sets from typical controls, as well as to examine pathologies of the nervous systems in relation to new standardized normative scales. Such new characterizations of mental illnesses respond to a recent paradigm shift in psychiatry neuroscience whereby neurodevelopmental disorders are now conceptualized as precursors of mental disorders (e.g., schizophrenia and related mental illnesses) emerging later in life (Paus et al., 2008; Insel, 2009, 2010; Casey et al., 2014)

One such repository is the Autism Brain Imaging Data Exchange (ABIDE) encompassing (in 2017) imaging and demographics data, including 17 sites in ABIDE I (http://fcon_1000.projects.nitrc.org/indi/abide/abide_I.html) and 19 sites in ABIDE II (http://fcon_1000.projects.nitrc.org/indi/abide/abide_II.html) (Di Martino et al., 2014). Indeed, data from ABIDE has been used to examine several new and important questions in autism. Recent studies have examined specific sex-based differences (Alaerts et al., 2016), differentiations in structural organization of the motor systems in light of repetitive behaviors (Supekar and Menon, 2015), cortical volume and gyrification (Schaer et al., 2015), among other analyses and characterizations of morphological parameters. Because of the large sample size, the new results have unprecedented statistical power (Torres and Denisova, 2016). Further, open access to these data has opened new avenues for replication and critical assessment regarding the reliability of clinical tests reported in the demographic data. Among such tests are the ADOS-2 and ADOS-G scores, IQ and medication status (Torres and Denisova, 2016; Torres et al., 2017).

One concern about image analyses has been the presence of motion artifacts distorting the images (Appendix Figure A1). Despite instructions to the participants to remain as still as possible, the human body is in constant motion (heartbeat, respiration, involuntary movements, etc.) Some excess motion may distort the image frame. For this reason, the head motion is tracked throughout the scanning session. Head motion can be extracted from the images time course using conventional methods (Friston et al., 1995; Worsley and Friston, 1995) and open-access software available to researchers (Cox, 1996). The head motion parameters are commonly used to determine the magnitude of the motion and set the threshold to eliminate frames contaminated by motion artifacts, a process coined in some circles “*scrubbing*.”

Scrubbing can create irregular gaps in the original time series of imaging data. Given individual differences in the amount of involuntary motions such as those of the head at rest, such gaps can vary by participant. As such, given a study with different demographics (e.g., autism and age-matched neurotypical controls in Appendix Figure A1) and owing to the excess motion in autism (Torres and Denisova, 2016; Torres et al., 2017), we would be comparing very disparate sizes of overall number of the clean image frames selected to be included in further analyses. In this way, one would be (unknowingly) skewing the statistical inference and further interpretation of the results. Moreover, because statistical inference may be affected by the non-uniform scrubbing across different sites, reproducibility of research may be compromised. Irregular gaps in the clean data could introduce different biases and give rise to very different outcomes even in cases when the two sites may have implemented an identical study, asked identical questions and recruited participants under identical inclusion/exclusion criteria.

Each person's cumulative involuntary head motion expends energy and in extreme cases (such as those in Appendix Figure A1) energy expenditure may incur in fatigue. We do not know if such energy expenditure would affect blood oxygenation and hemodynamic responses. Thus, we do not know what the cumulative consequences of such excess involuntary motions may be for energy expenditure, fatigue and the BOLD signal in general. At the very least, early in the pipeline of analyses, we can examine the stochastic properties of the original head motion time series data, before scrubbing takes place, and gain insights into the nature of the stochastic processes likely underlying the original time series.

The ABIDE data sets have not been scrubbed, so we have access to the original time series of the head motion data and can study the fluctuations in amplitude of the linear displacement and angular rotations of the head; i.e. as they were originally captured during the resting state of the fMRI sessions. These time series depend on the sampling resolution (SR) of the scanner, i.e. of the number of frames per unit time that the scanner captures, which are different in different sites of ABIDE. Since these differences in SR impact the variability of the speed-dependent data that is used to set thresholds for scrubbing, it is possible that different noise quality, denoting different types of underlying random processes (Stanley et al., 1999; Seely and Macklem, 2004; Perkiömäki et al., 2005) may be inherently present in the data (Appendix Figure A2). Characterizing the stochastic properties of the raw data is then important because the removal of motion artifacts depends on the threshold criteria derived from the head motion being tracked (Friston et al., 1996). In turn, as illustrated in Appendix Figure A2, different random processes underlying time series data may lead to diverse cumulative effects and give rise to inherent biases in thresholding the data to be eliminated from a given set.

One way to characterize the stochastic features inherent to the raw data is by empirically estimating the probability distributions underlying a parameter commonly used in the literature of motor control to investigate the nature of the variability in biophysical data. Such data include motion biorhythms extracted from signals harnessed from heart rate (Peng et al., 1995b) including local-scale of shorter time series (Castiglioni et al., 2007), gait (Hausdorff et al., 1997; Kaipust et al., 2013) including short time series (Qiu et al., 2016; Terrier, 2016), finger tapping (Botcharova et al., 2015), among others. The parameter of interest is the alpha exponent (explained in the methods) derived from Detrended Fluctuation Analyses (DFA) (Peng et al., 1995b), a popular method to examine stochastic processes and gaining insights on the self-affinity / stationarity (or lack thereof) of biophysical time series data (Stanley et al., 1999).

It is possible that the noise quality emerging from these analyses point to different types of random processes characterizing events captured by these time series under disparate sampling rates. For example, by capturing (or omitting) small rotations or displacements of the head with different frequencies per unit time (Appendix Figure A3), we may introduce different biases in the early scrubbing stage of the data processing pipeline. This stage cleans the images from motor artifacts. As such, to further proceed with data cleansing and statistical inference, one would need to take into consideration the different sampling resolutions of scanners in different ABIDE sites. ABIDE I and II comprise 26 sites with sampling resolution above 1 Hz; in contrast to 2 sites below 1 Hz. It suffices for us to examine two extreme cases to learn if variations in sampling resolution give rise to different ranges of noise. Having this knowledge could help researchers further design appropriate analyses for statistical inference, better standardize their methods and more generally increase the rates of research outcome reproducibility across labs.

The variable degree of skewness we have previously found in the empirical distributions of linear and angular speed peaks derived from these ABIDE data (Torres et al., 2017) sets motivated us to further explore the possibility that different random processes may underlie the time series data collected under different SR. As such, here we try to elucidate the quality of the noise inherently present in the variability of the speed-dependent raw data comprising all the original frames of the studies of ABIDE (i.e., without scrubbing the images).

Given prior results from other fields concerning differences in signals representing different kinds of random processes (Hausdorff et al., 1995; Havlin et al., 1995; Peng et al., 1995b), we here hypothesize that the differences in sampling rates of the scanners will affect the nature of the noise in the data. By noise, we specifically mean the *noise to signal ratio*, whereby to obtain the ratio, we empirically estimate the Probability Density Function (PDF), the mean and the variance characterizing the parameter of interest (the alpha index). This contrasts with assuming a given theoretical distribution (e.g., the Gaussian) or a given feature of the random process (e.g., stationarity). As a possible corollary of this proposition, we posit that within sites of similar SR, we may be able to use the noise range (empirically obtained from the raw data) to further characterize and differentiate neurodevelopmental disorders in the broad spectrum of autism relative to normative ranges. We report our results on combining and analyzing 2,154 participants from 28 sites of ABIDE I and II. Further, we provide evidence that different disorders can be well characterized by different noise qualities relative to age-matched typical controls.

## Methods

### Demographics of ABIDE I and II

All datasets included in this study are from the Autism Brain Imaging Data Exchange (ABIDE) databases: ABIDE I (http://fcon_1000.projects.nitrc.org/indi/abide/abide_I.html) and ABIDE II (http://fcon_1000.projects.nitrc.org/indi/abide/abide_II.html). The work obeys Frontiers guideline on the use of human subject's data. To that end, citing from ABIDE “In accordance with HIPAA guidelines and 1000 Functional Connectomes Project / INDI protocols, all datasets have been anonymized, with no protected health information included.”

**Figure 1:**
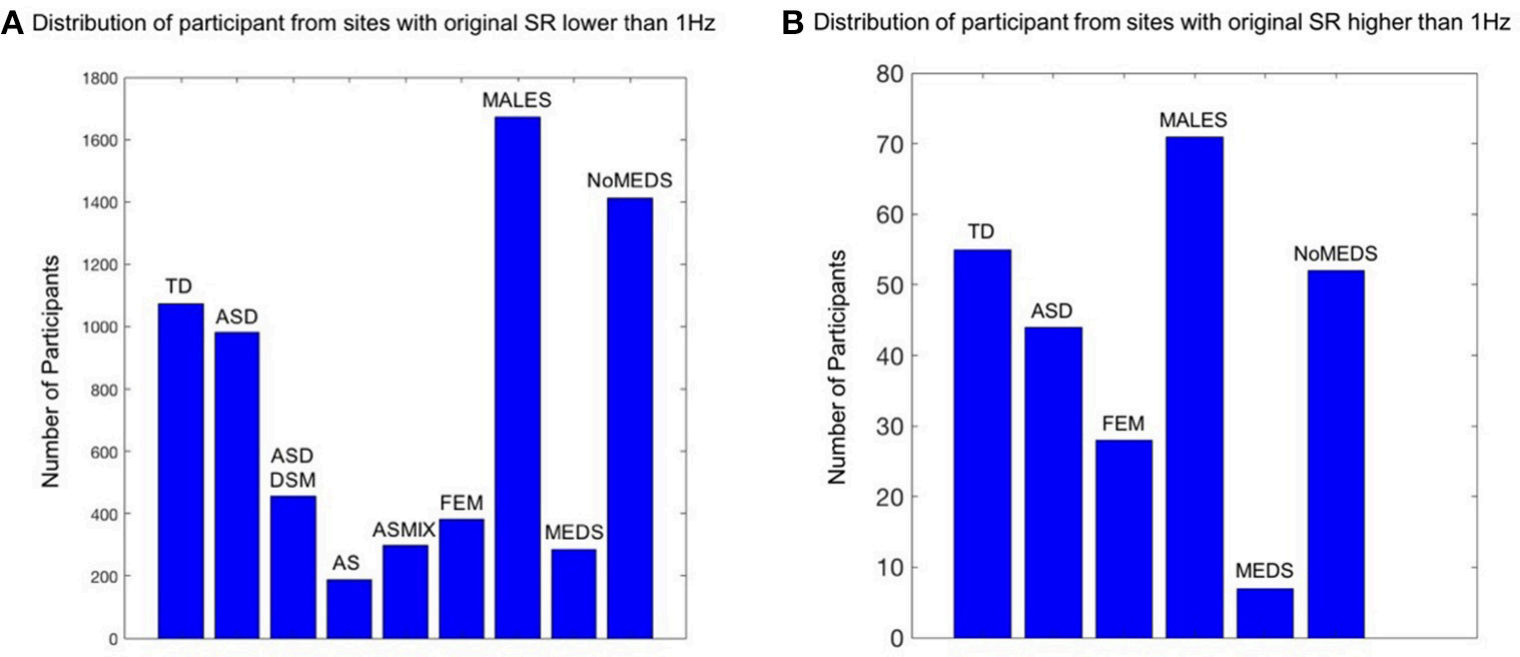
Inclusion / Exclusion criteria for the ABIDE I and II data sets used in this study. Inclusion / Exclusion criteria for the ABIDE I and II data sets used in this study. **(A)** Participants from the sites from ABIDE I and ABIDE II which collected the fMRI data using a sample resolution lower than 1 Hz. TD refers to typically developing participants (SR0 *n* = 1074; SR1 *n* = 55); ASD refers to Autism Spectrum Disorders according to the column 1 DSM of demographic records across ABIDE I and II (SR0 *n* = 982; SR1 *n* = 44). ASD_DSM_ indicates those participants from the column 2 of demographic records with a DSM-IV-TR ASD diagnosis (SR0 *n* = 456); AS refers to the individuals from DSM-IV-TR with Asperger's diagnosis of column 2 of demographics records (SR0 *n* = 189); ASMIX includes all AS, (Pervasive Developmental Disorder Not Otherwise Specified (PDDNOS), Pervasive Developmental Disorder (PDD) from column 2 DSM-IV (no ASD from DSM IV) (SR0 *n* = 298). FEM refers to all the female participants of demographic records across ABIDE I and II, despite the diagnosis they have (SR0 *n* = 384; SR1 *n* = 28); MALES are all male participants of demographics records, despite the diagnosis they have (SR0 *n* = 1673; SR1 *n* = 71); NoMEDS refers to all with a diagnosis of ASD (column 1 and 2 of the demographics records), all with a diagnosis of AS or PDDNOS or PDD who were not on medication (i.e., from all sites that reported medications) (SR0 *n* = 1414; SR1 *n* = 52). MEDS refers to all participants with any diagnosis but not on medication (SR0 *n* = 285; SR1 *n* = 7). **(B)** The same as **(A)** for participants from the sites which collected the fMRI data using a sample resolution higher than 1 Hz.

The main breakdown of demographics used in this study is summarized in Figure 1. The study includes four main comparisons:

1. Sampling rate (SR) lower than 1 Hz *vs*. SR higher than 1 Hz. are shown in the Table S1 which provides information regarding the reported scanner SR for each site.
2. *Autism Spectrum Disorder (ASD), Asperger's Syndrome (AS), Typical Development (TD)*, using estimation of noise signatures extracted from head excursion of individuals with a formal DSM-ASD, a DSM-IV-TR (American Psychiatric Association, 1994) diagnosis of AS and TD controls.
3. *Medication vs. no Medication*, including individuals with any diagnosis who reported medication use *vs*. those who reported no-medication use (we question if the noise of their involuntary head motion is affected by the medication status.) Table S2 lists the sites that contain medication-intake information.
4. *Females vs. Males*, using the above-mentioned metrics and selected across ABIDE based on the inclusion/exclusion criteria defined below.

### Inclusion/exclusion criteria

This study includes all sites publicly available through ABIDE I and ABIDE II (as of November 2017). Because datasets were independently collected at each side, varying types of phenotypic information was provided. For example, DSM-IV-TR diagnosis and medication intake were reported at some sites, but not at others. For this reason, the number of participants within each comparison group may be different from the reported amount (see Table S3 for reported quantities). We denote SR <1 Hz (SR0) and SR>1 Hz (SR1).

### Bootstrapping method

Given the inconsistent group sizes extracted from the ABIDE datasets (see Appendix Figure A5) we used bootstrapping to ensure uniform group numbers for *pairwise statistical comparisons* across diagnoses and demographics.

### Data processing

#### Motion extraction

Head motion patterns were extracted from imaging data during resting state (rs) fMRI experiments. Motion extraction was performed using the Analysis of Functional NeuroImages (AFNI) software packages (Cox, 1996). Single-subject processing scripts were generated using the afni_proc.py interface^1^. Skull stripping was performed on anatomical data and functional EPI data were co-registered to anatomical images. The median was used as the EPI base in alignment. Motion parameters, 3 translational (x, y, and z) and 3 rotational (pitch-about the x axis, roll-about the y axis, and yaw- about the z axis), from EPI time-series registration was saved (step 1 of the Figure S1).

#### Head excursion analyses

To ensure uniformity across all data sets, we resampled and truncated the time series data thus creating data sets of equal numbers of points and equal spacing between points (Figure S1). Appendix Figure A3 shows visible differences in speed data sampled from SR0 and SR1 groups, thus suggesting the need to assess noise quality in the data gathered with different frequency.

To obtain the head excursions we sum over the speed profiles thus yielding the path length of the linear displacements as well as the full excursion of angular displacements. This gives us a sense for the net amount of physical motion a person had. In both cases we used the same number of points for each participant.

##### Speed profile

We computed the rate of change of linear displacement and angular rotation using the Euclidean norm to compute the magnitude of each 3-dimensional velocity vector displacement (Δ*x*, Δ*y*, Δ*z*) at each positional point of application (x, y, z) from frame to frame, for 370 frames (the same was done with the orientation trajectory). The scalar magnitude of the linear speed *s* was defined as for a common unit time:

(1) $$\begin{array}{r} s=\sqrt{{\left(\Delta x\right)}^{2}+{\left(\Delta y\right)}^{2}+{\left(\Delta z\right)}^{2}} \end{array}$$

In order to preserve the original temporal dynamics of the first rate of change data while smoothing the sharp transitions from frame to frame, we adopt the method from (Wu et al., 2014). This method filters the position data using a triangular window:

(2) $$\begin{array}{r} v^{\prime }\left(i\right)=\frac{\overset{d}{\underset{k=-d}{\sum }}\left(v\left(k+i\right)\cdot \left(d+1-|k|\right)\right)}{\overset{d}{\underset{k=-d}{\sum }}\left(d+1-|k|\right)} \end{array}$$

for velocity *v* of frame *i, k* summation index from –*d* to *d* and testing various values of *d* e.g., up to *6*, to build a symmetrically weighted sum around the center point, frame by frame (sample outcome is shown in step 2 of the Figure S1).

##### Uniformly resampled data sets

We resample all data to ensure equally spaced points for comparison across subjects and groups (outcome is shown in step 3 of the Figure S1. To that end, we use MATLAB (version R2014a, The MathWorks, Inc., Natick, MA) function *resample* which applies an antialiasing FIR low-pass filter to the time series and compensates for the delay introduced by the filter. This function resamples the input sequence, the raw head motion in our case, at P/Q times the original sample rate (see Table S1 of the SM for more information about the resampling factors used [P and Q]).

##### Uniform data length

We truncated the uniformly resampled data to ensure the same length for all the time series. The shortest time series had 370 data point, thus all the data samples were shortened by this length (sample outcome shown in step 4 of the Figure S1).

#### Noise signature estimation

**Figure 2:**
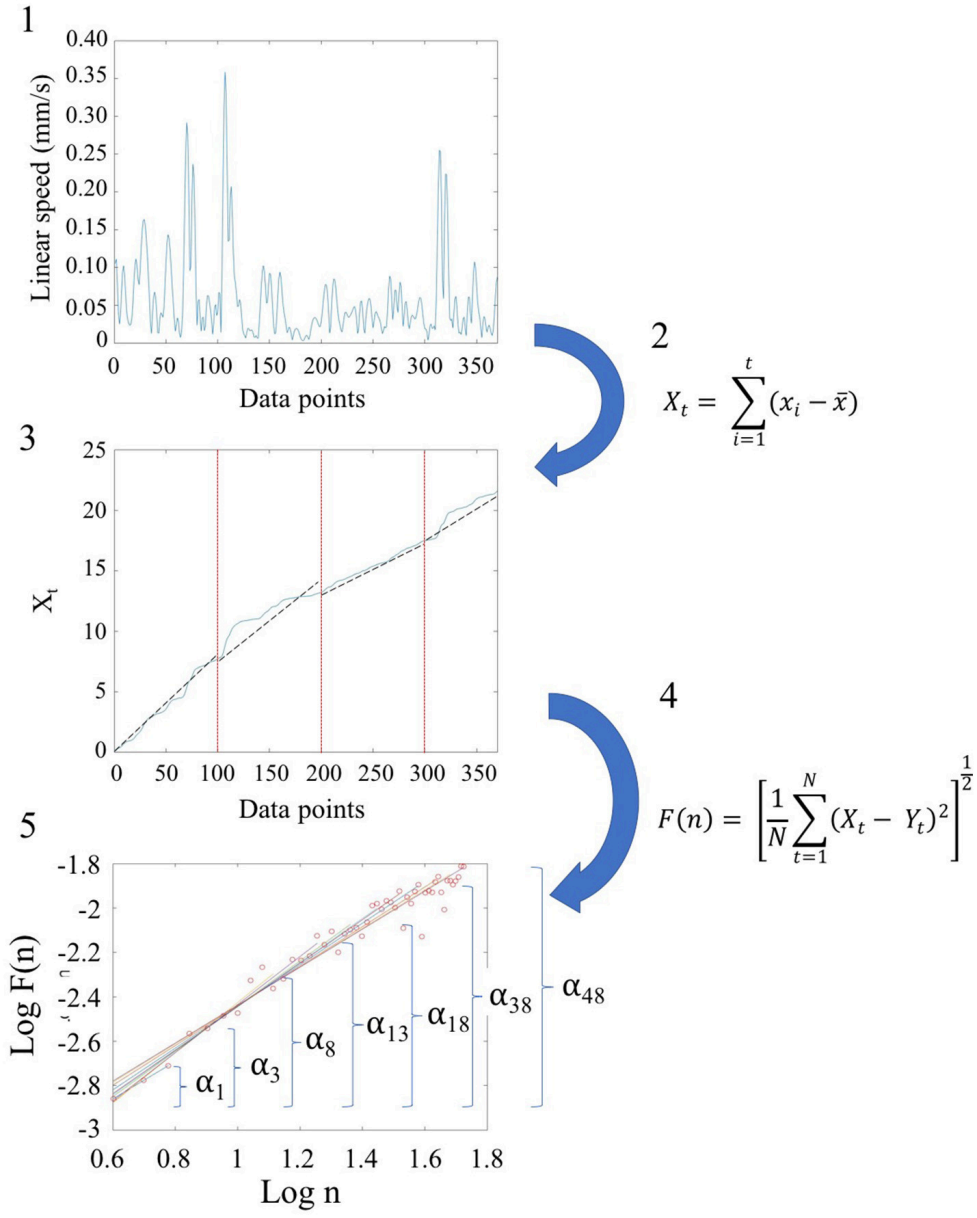
Schematic steps to perform the Detrended Fluctuation Analysis (DFA) using the linear and angular speed profiles of the involuntary hear excursions. (Step 1) Sample raw linear speed data extracted from linear positional displacements of the head along the x-, y-, and z-axis. The magnitude of the rate of displacements frame by frame (the linear speed temporal profile) is obtained and the profile resampled at 2 Hz. The data is truncated to 370 points for all participants to ensure equal number of points (see Figure S1); (Step 2) Given the time series of length *N* = 370, (the minimum number of points across the data set) we obtain the integration or summation within 100-point window for each $X_{t}=\overset{t}{\underset{i=1}{\sum }}\left(x_{i}-\langle x\rangle \right)$ where t denotes the size of the window, *x*_*i*_ is each point in the series within the window and 〈*x*〉 is the overall mean across the entire time series with linear speed (empirical range bounded between 4.28e-05 and 10.14 mm/s over the entire data set.) The *X*_*t*_ is the cumulative sum or profile and the summation converts from a bounded time series to unbounded process. (Step 3) The cumulative profile *X*_*t*_ is divided into non-overlapping time windows of equal length *n* (range 4 ≤ *n* ≤ *N*/10) where *N* is the total number of points in the signal, which in our case is *N* = 370 (Chen et al., 2002). In each interval, a local least-squares straight-line fit (which is the local trend) is obtained using minimization of the least squares errors in each window. The resulting piecewise sequence of straight line fits is denoted *Y*_*t*_, then we calculate the root-mean-square deviation from the trend, i.e., the fluctuation: $F\left(n\right)=\sqrt{\frac{1}{N}\overset{N}{\underset{t=1}{\sum }}{\left(X_{t}-Y_{t}\right)}^{2}}$. (Step 4) The above process of detrending and obtaining the fluctuation metric is repeated over a range of different window sizes and a log-log map of *n* vs. F(*n*) obtained. This map provides a relationship between *F(n)*, the average fluctuation as a function of box size, and the box size *n*. As explained in (Peng et al., 1995a), the straight line of this log-log relation indicates statistical self-affinity expressed by the scaling exponent alpha, *F*(*n*) ∝ *n*^α^. The exponent alpha (a generalization of the Hurst exponent Hurst, 1951, is a measure of long time memory in a time series) is the slope of the straight line fit to the log(*n*) vs. log*(F(n))* relation using least squares. (Step 5) To obtain a series of alpha values for each participant, we windowed the data starting with 3 points, then 4 points, then 5 points, etc. to the maximum number of points (370) we had.

We used Detrended Fluctuation Analysis (DFA) (Peng et al., 1995b) to quantify the possible presence of long-range power-law correlations in the time series signals, estimating the scaling index, α, describing the quality of noise that has been physiological characterized in the literature (see Appendix Figure A2). The steps of DFA are listed and illustrated in Figure 2. These graphs are implemented in MATLAB (version R2014a, The MathWorks, Inc., Natick, MA). In addition to MATLAB, we reproduced the outcomes using Python. Python code to compute DFA is provided in the following link: https://gist.github.com/JVero/9bb4921eeaefba8f0edff41cb584b460. Further, the Supplementary Material Figures from Python Code shows the graphs we generated in Python.

##### Statistical analyses

In the present work, we assess alpha scaling index derived from the scan-by-scan speed-dependent variations in the linear displacement and in angular rotations of the head during rs-fMRI sessions. We estimate their long-range power-law correlations in signals (noise characteristics, computed as explained above using DFA) and infer statistical features using empirical statistical estimation (see below).

To ascertain the net physical head motions across all participants, we obtain the path length of the linear and angular displacements (as explained above). The empirically estimated mean was obtained using the continuous Gamma family of probability distributions for every group (as in Torres et al., 2016; see Table 1 for information about the mean head excursion for the main groups).

**Table 1:** Physical values of linear and angular excursions of involuntary head motion detected during the resting state fMRI sessions for each group of all the sites in ABIDE I and II.

**Groups**	**Linear (mm/s)**	**Angular (deg/s)**
**ALL SITES**		
TD	0.0124	0.0118
ASD	0.0199	0.0177
ASD_DSM_	0.0221	0.0187
AS	0.0186	0.0159
ASMIX	0.0177	0.0147
MEDS	0.0242	0.0199
NoMEDS	0.0151	0.0140
FEMALES	0.0138	0.0128
MALES	0.0165	0.0150
MEDS FEM	0.0209	0.0192
MEDS MALES	0.0247	0.0200
NoMEDS FEM	0.0137	0.0125
NoMEDS MALES	0.0154	0.0144

*TD, Typical development; ASD, Autism spectrum; AS, Asperger Syndrome; ASMIX, combination of participants with a diagnosis of Asperger Syndrome or Pervasive Developmental Disorder not otherwise Specified; FEM, Females; MEDS, On Meds; NoMeds, Off Meds; DSM, Diagnostic and Statistical Manual of Mental Disorders*.

The raw linear and angular speed profiles (i.e., the time series) of each subject resampled to the same rate for all participants and truncated to the same number of points for all are input to the DFA to obtain distributions of α-values per individual. These were pooled across each of the subgroups generated by the *Bootstrapping method* explained above keeping in mind the SR0 and SR1 types. Thus, we obtained a distribution of α values for each group compared for each SR.

We first compare the SR0 and SR1 groups (see Supplementary Material for the statistical results of each group and comparisons, Tables S4, S5 for the linear and angular speed respectively). Then we compare the different groups according to diagnosis, sex or medication (see Supplementary Material for the statistical results of each group and comparisons, Tables S6–S9 for sites with SR0, and Table S10 for the sites with SR1).

We examine the frequency histograms of the α-values and use maximum likelihood estimation (MLE) to approximate the best fitting distribution encompassing all cases. To that end, we compare different families of probability distributions (e.g., the Gaussian, Normal, Lognormal, Exponential and Gamma) and choose the best fit in an MLE sense. Owing to our prior works using the ABIDE sets (Torres and Denisova, 2016; Torres et al., 2017) we determined that the Gamma had the best fit in an MLE sense. As such, we settled on the continuous Gamma family of probability distributions (Ross, 1983). We estimate the shape and the scale parameters and plot them on the Gamma parameter plane. The estimated parameters with their CI were plotted on a Gamma parameter plane, where the *x*-axis represents the shape parameter value and the *y*-axis represents the scale parameter value. The Gamma scale value conveys the *noise to signal ratio* (NSR) since the Gamma mean μ_Γ_ = *a* · *b* and the Gamma variance is $\sigma_{\Gamma }=a\cdot b^{2}$, thus the scale is:

(3) b=σΓμΓ=a⋅b2a⋅ b

In this sense, the Gamma parameter plane allows us to infer speed-dependent processes leading to higher noise levels *vs*. lower noise levels. Further, since higher shape values tend toward symmetric distributions and lower values tend to be skewed distributions, with the extreme Exponential distributions at *a* = 1, we can also track processes that tend to the Exponential (most random) *vs*. processes that tend toward the Gaussian distribution (more predictable).

Using the empirically estimated Gamma moments (mean, variance, skewness and kurtosis) which we then plot, for each pairwise-group comparison, as a four-dimensional parameter space using the x-axis as the mean, the y-axis as the variance, the z-axis as the skewness and the size of the marker as the kurtosis. We also plot the Gamma PDFs using the empirically estimated parameters.

## Results

### Different sampling rates give rise to different noise types representing different random processes

**Figure 3:**
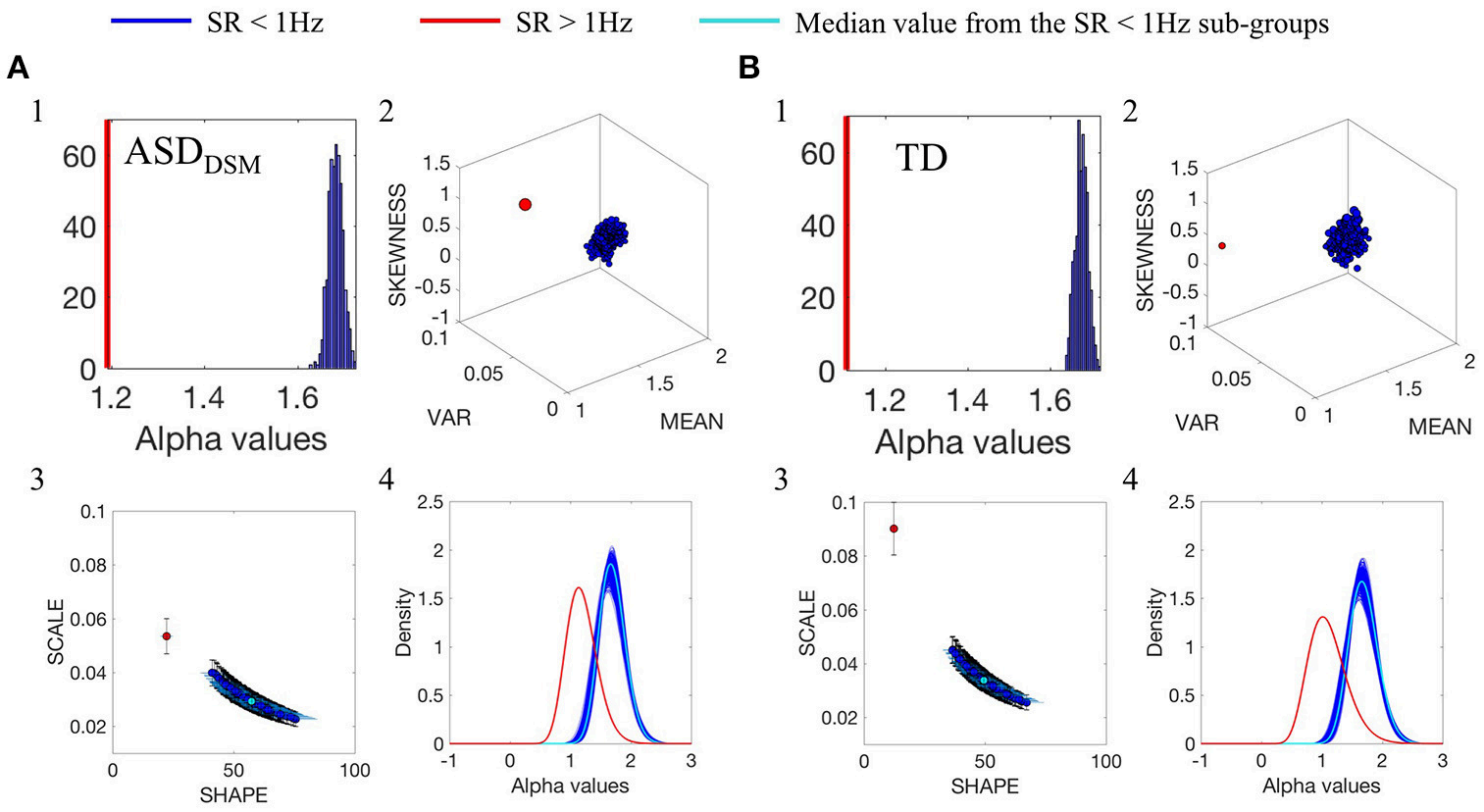
Comparison between the distributions of α values of the linear speed for the groups with different original SR. **(A1)** Frequency histogram of the mean α values from the 500-sub-groups extracted from ASD_DSM_ participants from SR0 group (large group) using bootstrapping, compared with the mean of 44 ASDDSM participant from SR1 group (small group, represented by the red vertical line). **(A2**) Empirically estimated Gamma moments (marker size is the kurtosis). Red dot is estimated from the ASD_DSM_-SR1smaller group. Blue cluster is the ASD_DSM_-SR0 large group with 500 sub-groups built using bootstrapping method described in Figure 3 while preserving the age binning composition of each sub-group to match that of the small group. **(A3)** Estimated points on the Gamma parameter plane with 95% confidence intervals. Red point is the ASD_DSM_-SR1 small group while blue dots are from the ASD_DSM_-SR0 500 subgroups from the large group. Cyan is the median value. **(A4)** PDFs obtained from the estimated shape and scale Gamma parameters. **(B)** TD group participants with similar format as **(A)**.

The comparison between data from the SR0 and SR1 sites yielded significantly different results when matching the ASD and the TD groups of each of the sites. The results can be appreciated in Figure 3A(1–4) for the ASD groups and in Figure 3B(1–4) for the TD groups. In each group, we compared the smaller age-matched SR1 group with each of the 500 SR0 age-matched sub-groups drawn at random with replacement from the larger SR0 group. The distribution of alpha values separated between the two ASD and the two TD groups, as did all estimated Gamma parameters, PDF's and moments. Along the Gamma parameter plane, the SR0 ASD participants had much lower noise and higher symmetry than those in the SR1 set of ASD participants. This was the case too for the TD controls. This higher dispersion could be explained by the excess variance that accompanied lower mean values in both the ASD and TD comparisons. This can be appreciated in Figure 3A(2) for the ASD cases and Figure 3B(2) for the TD cases. Differences in kurtosis (the size of the marker) are appreciable in the ASD group comparison whereby the SR1 yielded peakier distribution than the SR0, while in the TD comparison both SR1 and SR0 had comparable kurtosis.

Please refer to Figures S2, S3 and Tables S4, S5 and for additional comparisons between SR0 and SR1 groups.

### Noise values differ between TD and ASD_DSM_

**Figure 4:**
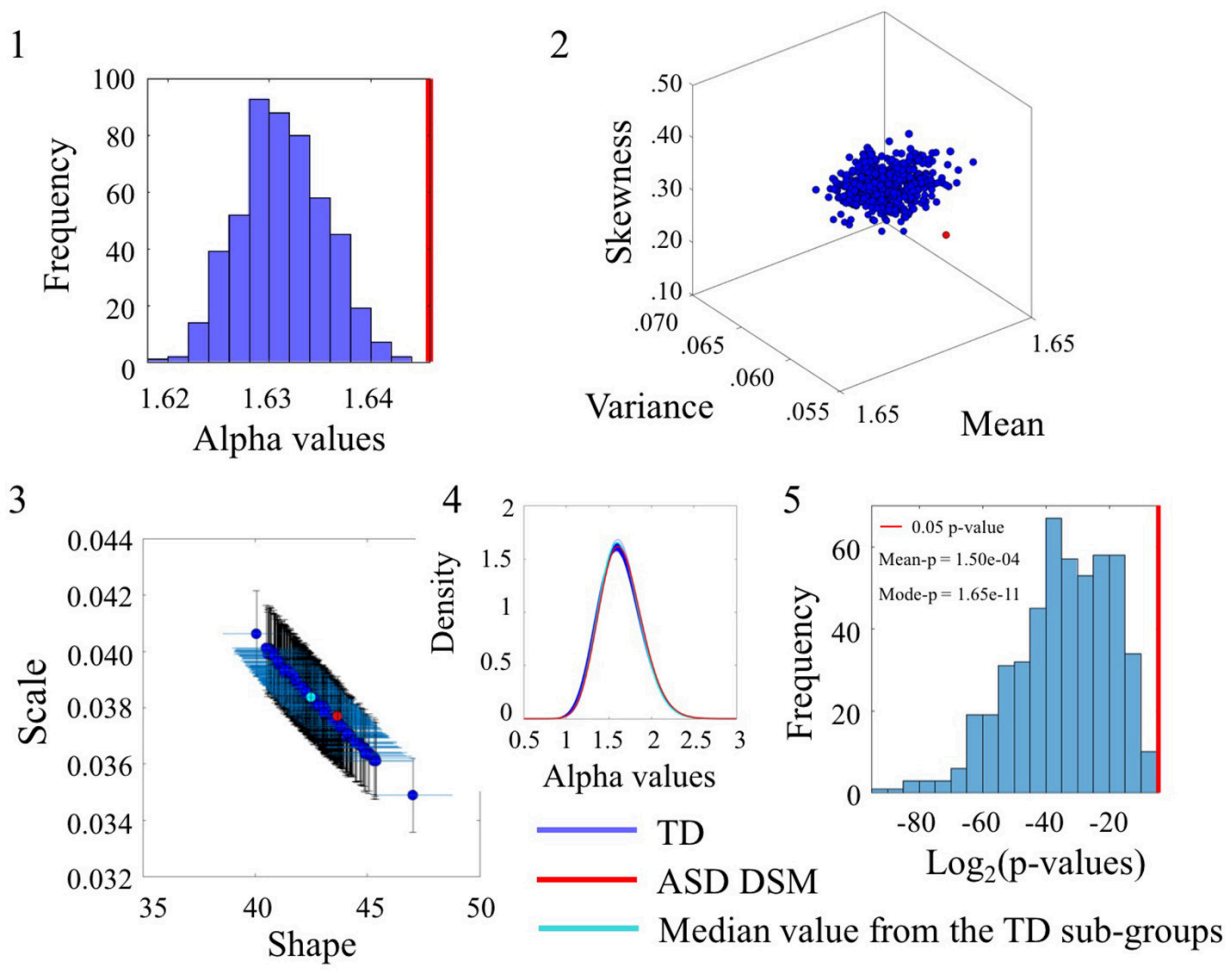
Comparison of the α values between TD and ASD_DSM_ groups for the Angular Speed (SR0 groups). **(1)** Distribution of the mean of the alpha values for the 500 sub-groups extracted from original TD group with similar size and age composition as the ASD_DSM_ group. The red vertical line represents the mean of the alpha values of the ASD_DSM_ group. **(2)** Estimated Gamma moments (red dot for the small group, blue cluster of 500 dots from the bootstrapping with 456 age-matched participants in each sub-group and the cyan dot for the median of those sub-groups). **(3)** Estimated shape and scale Gamma values with confidence intervals on the Gamma parameter plane. **(4)** Estimated PDFs. **(5)** Distribution of *p*-values (log2 scale for better visualization) with the red line as the reference 0.05 significance-level value. The mean and mode of the *p*-value distribution is also provided.

The comparisons of different diagnoses within the data sets from the SR0 sites yielded differences in the alpha parameters that were also quantifiable in the empirically estimated statistical signatures. Figure 4 focuses on the differences between the TD and ASD_DSM_ groups. Here the TD group is the larger of the two. As such, 500 points (blue dots in the Figures 4(2,3) are derived from the TD group and each case is compared with the point (red) generated by the age-match ASD_DSM_ group of 456 participants. The results shown in Figure 4 capture the statistically significant differences for the angular speed parameter (see the linear speed results in the Figure S4 and Table S6). Figure 4(1) shows the distribution of alpha values spanning from 1.61 to 1.65 for the TD groups vs. 1.66 for the ASD-DSM participants. Table S3 reports the number of participants included in each comparison.

The Figure 4(2) shows the estimated Gamma moments for the alpha parameter showing the cluster of TD participants in contrast to the ASD_DSM_ values. The ASD_DSM_ distribution (red dot) had comparable mean to the TD centroid but on average lower variance and skewness than the centroid. The overlapping of the ASD_DSM_ with a subset of the TD controls can be appreciated in Figure 4(3) where the estimated Gamma shape and scale parameters overlap between ASD_DSM_ and a subset of the TD controls. The estimated PDFs displayed in Figure 4(4) also confirm the overlapping in variability. The statistically significant differences in the ranges of alpha values between the ASD_DSM_ and the TD subgroups can be appreciated in the distribution of *p-values* displayed in Figure 4(5). The red line indicates 0.05 significance level. The subsets of TD overlapping with the ASD_DSM_ can be seen at the rightmost tail of the distribution. These are the TD subgroups for which the two-sample Kolmogorov-Smirnov test using the empirical data yielded *p* ≥ 0.05 when comparing the alpha values to those of the age-matched ASD_DSM_ set.

### Noise values differ between TD, AS, and ASD groups

**Figure 5:**
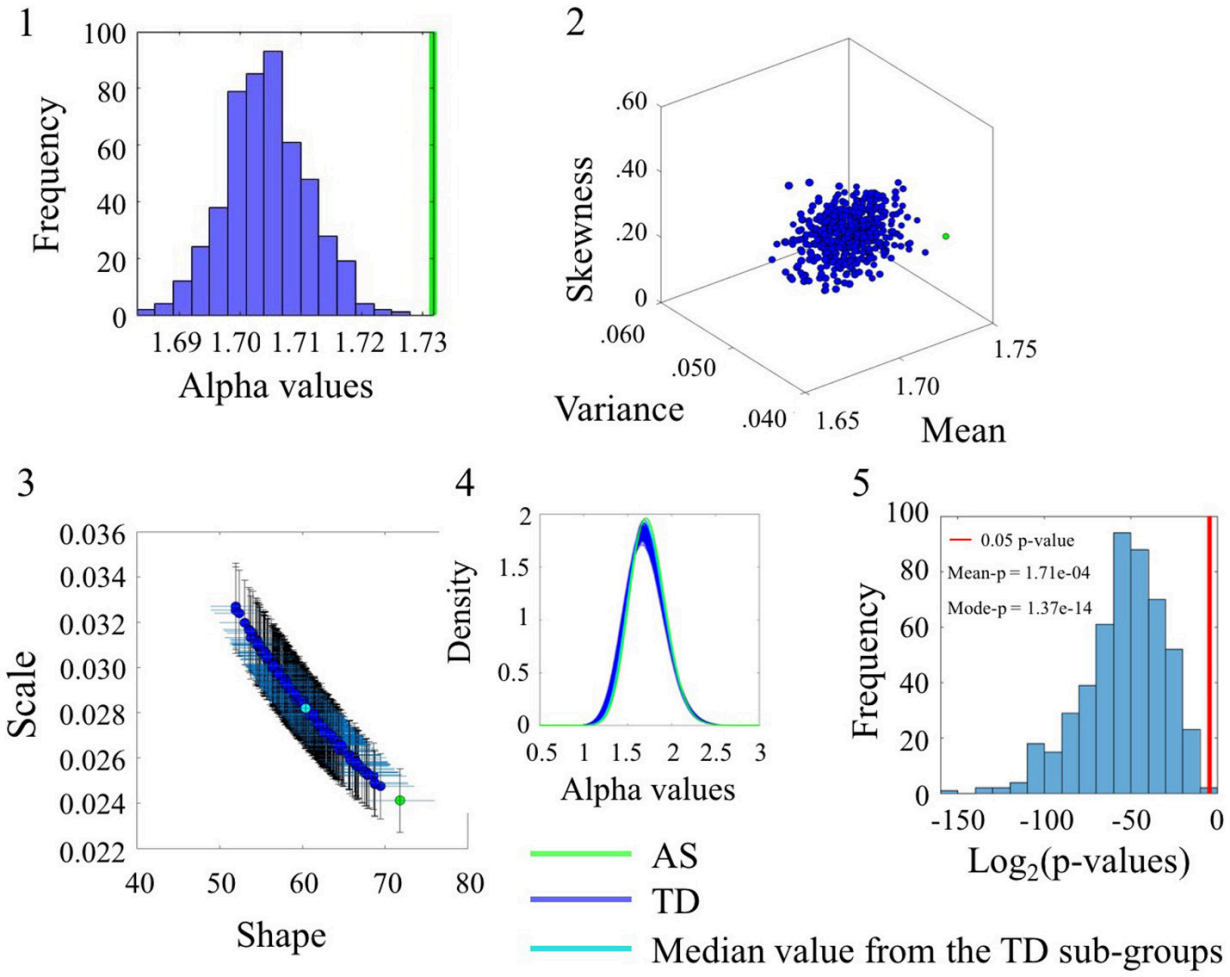
Comparison between TD and AS groups for the Linear Speed (SR0 groups). Figure format similar to Figure 4.

The comparison between the AS and TD participants in the SR0 sites also yielded significant statistical differences. These are captured in Figure 5 for the linear speed using the same format as in Figure 4. (Please see Figure S5 and Table S6 to examine the angular speed results). Notably the range of alpha values is different than those of the age-matched TD group built to accommodate the age composition of the AS group. Here the AS alpha is at 1.73 in contrast to the ASD participants of Figure 4 with lower alpha of 1.66. In all estimated parameters, the AS values showed a separation from the TD and it was significant at the 0.05 level for most TD-subgroups according to the Figure 5(5) distribution of *p*-values.

**Figure 6:**
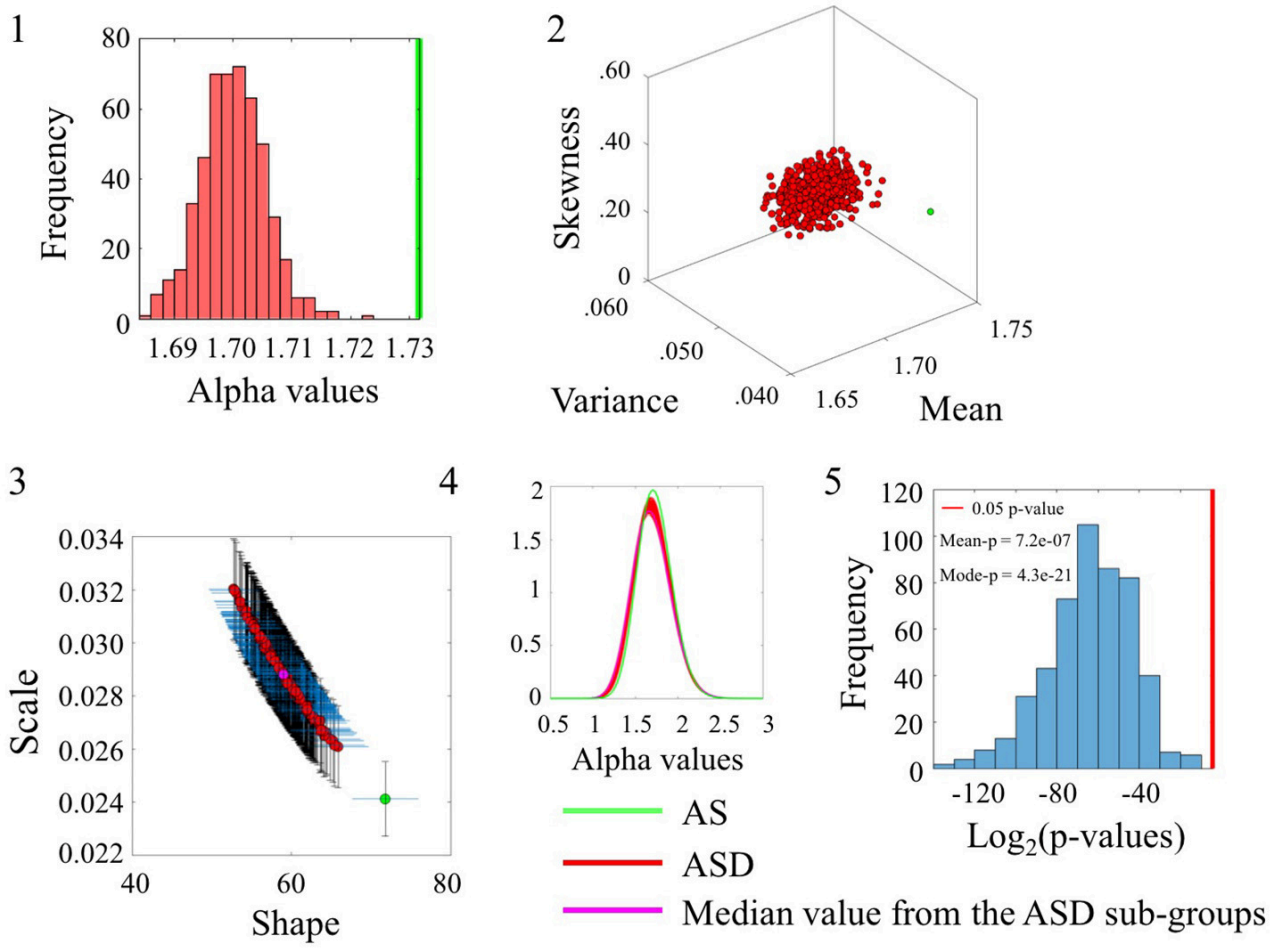
Comparison between ASD_DSM_ and AS groups for the Linear Speed (SR0 groups). Figure format is similar to Figures 4, 5.

Comparison between the ASD and AS groups whereby the ASD is the larger group confirmed the significance in statistical differences for the alpha range and the inherent variability of the underlying linear speed parameter across these sets from the SR0 sites. These differences can be appreciated in Figure 6 using the same format as in the previous comparisons. The distribution of *p-values* in Figure 6(5) shows the separation of the AS group from the ASD cohort for each one of the 500 ASD-subgroups the bootstrapping yielded. (Please see Figure S6 and Table S6 to examine the angular speed results).

### Some noise values overlap between males and females

**Figure 7:**
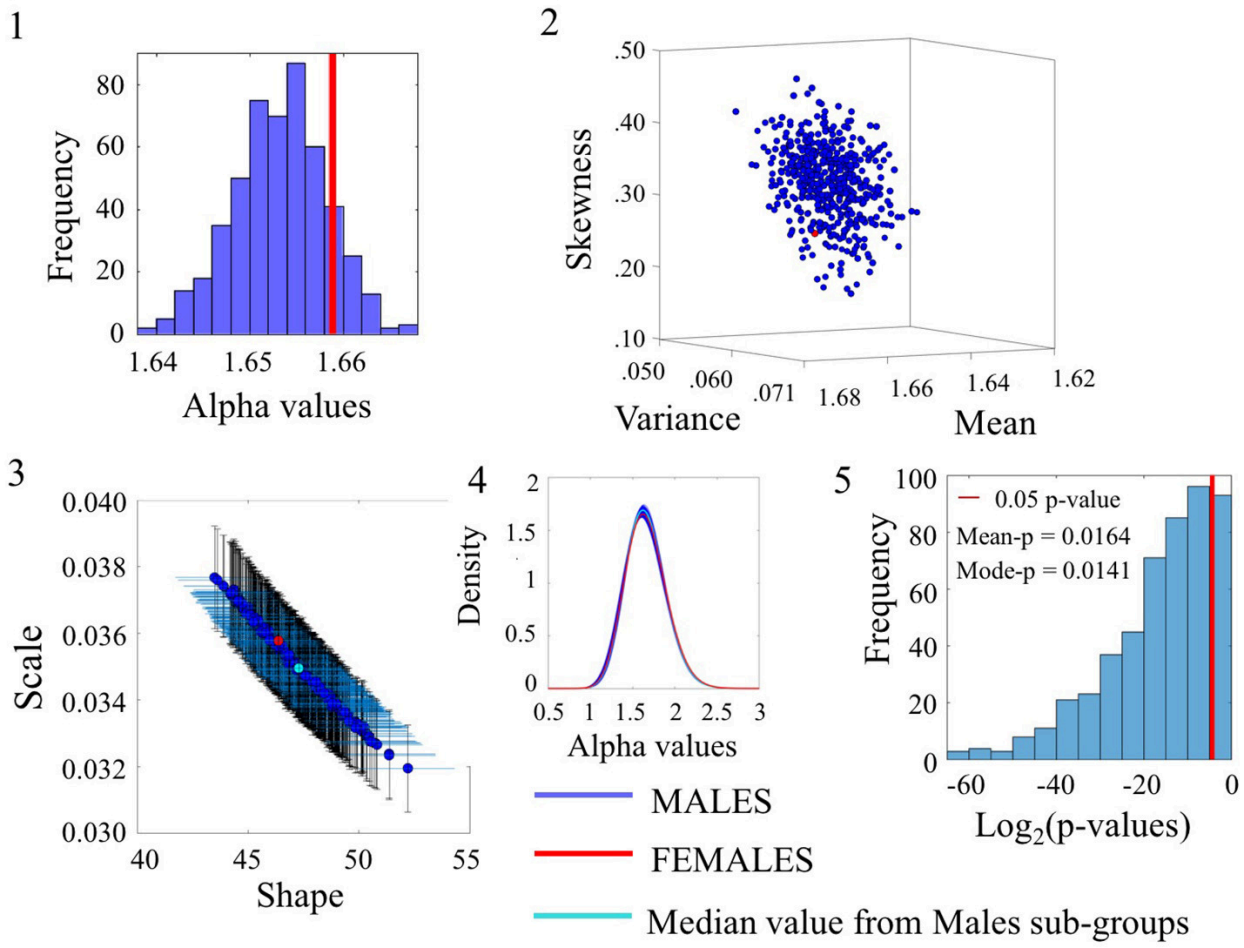
Comparison between Female and Male groups for the Angular Speed (SR0 groups). Figure format similar to Figures 4–6.

Examination of the smaller group of females in relation to the lager group of males for the angular speed case yielded some degree of overlapping whereby the alpha values of the females fell within the range of the ASD alpha values in Figure 7(1). The overlapping in *p-values* can be appreciated in Figure 7(5) for a subset of the comparisons (90/500). Please refer to Figure S7 and Table S6 for comparison of linear speed.

### Some noise values overlap between reported medication and no-medication

**Figure 8:**
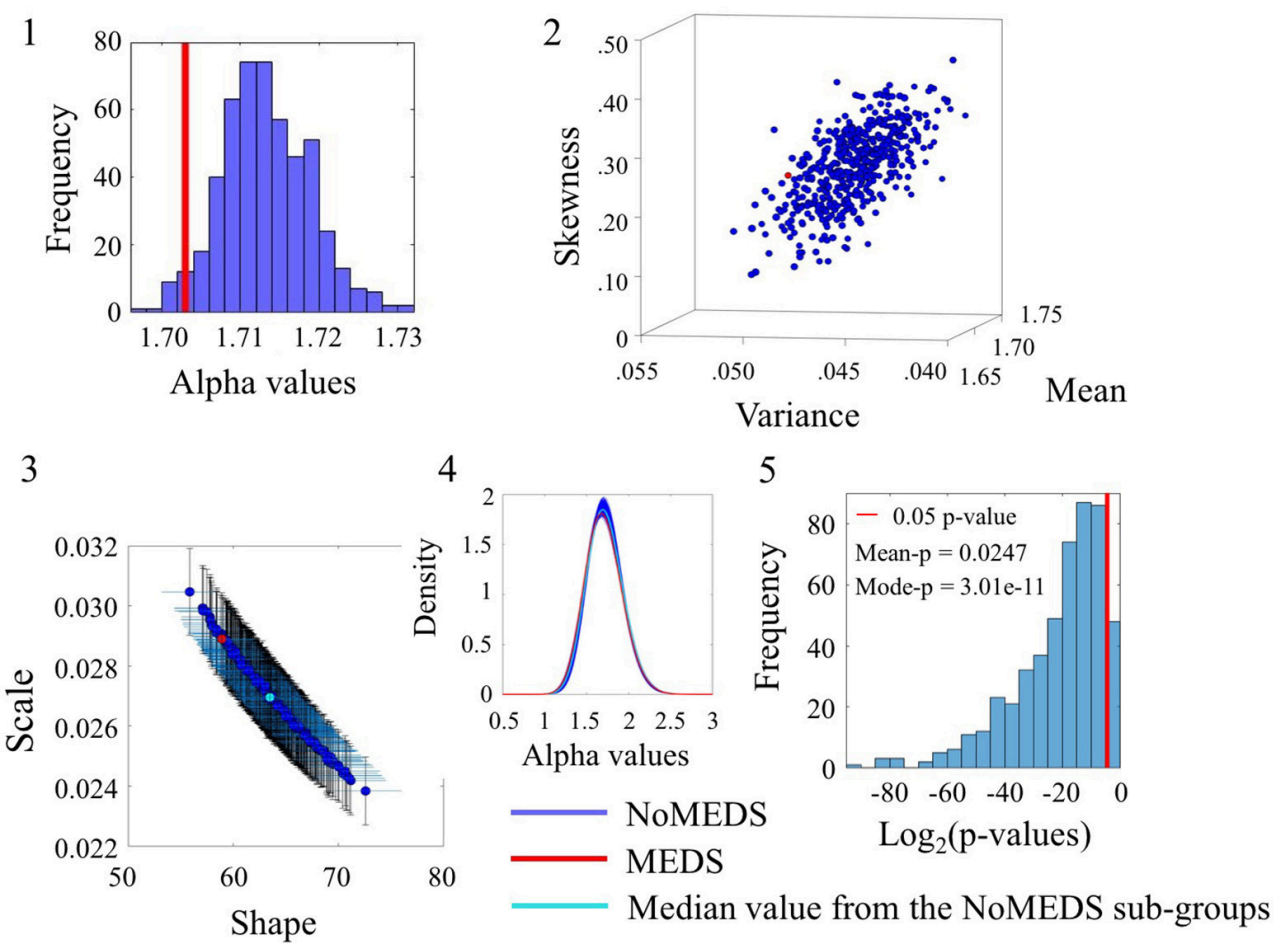
Comparison between Meds and NoMeds for the Linear Speed (SR0 groups). Figure format similar to Figures 4–7.

The comparison of participants who reported medication intake (MEDS-smaller group) vs. those who did not report medication intake (NoMEDS-larger group) also yielded some overlap in the comparison of alpha values derived from the time series of the linear speed parameter. This can be appreciated in Figure 8(1) where most NoMEDS subgroups had lower alpha values but a small subset of 50/500 had larger or equal alpha values than the MEDS group. Further in Figure 8(5) the *p*-values for the 50/100 groups show values over the 0.05 level of significance. Other comparisons from alpha values derived from the linear speed time series can be found in the Figure S8 shows the results for linear speed. Tables S6–S10, provide exhaustive and systematic examinations of the Gamma moments obtained from the data sets reported here. Table S11 discloses the Gamma moments obtained from the data sets before applying the *Bootstrapping method*.

For more information about the comparisons between the different analyzed groups, refer Figures S9–S16 for Distribution of the α values; Figures S17–S24 for Distribution of the *p*-values; Figures S25–S32 for estimated Shape and Scale Gamma values; Figures S33–S40 for PDFs; and Figures S41–S48 for estimated Gamma moments (mean, variance and skewness).

**Figure 9:**
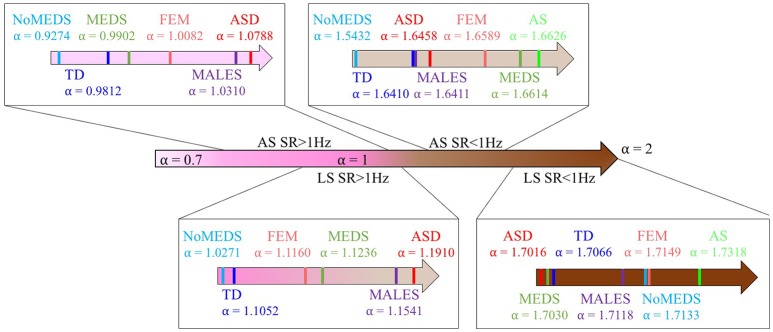
Summary of the mean of alpha values from each group from all sites from ABIDE I and ABIDE II and their relationship with the different types of noise in Appendix Figure A2.

A summary of alpha ranges and noise types referring the reader to the classes of random processes cited in Appendix Figure A2 can be appreciated in Figure 9. Here we show the full range of comparisons and the localization of each group along the alpha scale for each of the SR0 and SR1 sites in ABIDE I and II.

## Discussion

This paper addressed the question of whether the disparity in sampling resolution across different sites in the ABIDE repository would affect the quality of the noise-to-signal ratios empirically derived from the fluctuations in the amplitude of head motion speed. The main motivation for this question is the common use of speed amplitude as criterion to threshold the scrubbing of the imaging slides early in the pipeline of fMRI analyses. The disparate SR resulting in statistically different alphas strongly suggests adhering to SR that are comparable when pooling data and thresholding motor artifacts before further statistical inferential analyses.

To address this question, we used DFA to estimate the scaling (alpha) exponent values for the time series of linear and angular speed peaks defining the envelope (amplitude) of this signal. These were obtained from the head motions extractable from the rs-fMRI data using traditional methods from the imaging community. More specifically, such methods are commonly employed to detect excess in head motion and remove motion artifacts from the set of images acquired in the imaging session. This pre-processing stage eliminates “contaminated” frames and selects the remaining frames for statistical inference in subsequent image analyses (e.g., functional connectivity, structure and morphology, among others). The methods presented in the paper also address the disparity in clinical group sizes within diagnoses of the ABIDE demographics, thus providing a way to enable appropriate statistical comparison of groups while using similar size and age composition according to the various DSM and phenotypical criteria listed in the demographics data of ABIDE.

Given the wealth of information of the ABIDE repository, many more comparisons and other parameters could have been explored. Here we focused primarily on ASD (DSM-IV and non-DSM-IV), AS, TD, FEMALES, MALES and MEDS, No MEDS reports. These groups served to illustrate that:

1. The sampling resolution of the scanner ***does*** affect the type of random process underlying the time series of imaging data and the parameters one can derive from it (in this example we used head motion in the form of displacements and rotations).
2. Given comparable sampling resolution, the noise quality inherent in the time series of the head motion parameters and their overall stochastic signatures may serve to further characterize different diagnoses and sex- or medication-related data.

The statistical estimation procedures using comparable group size and age composition uncovered a gradient of alpha values toward the ranges of fractional Brownian motion (fBm) for the sites with sampling resolution below 1 Hz (denoted SR0) (Figure 9). Most values fell above 1.5 alpha-ranges indicating persistent fBm (Appendix Figure A2). This contrasted with lower ranges of alpha values tending instead in the opposite direction, toward the pink noise range for sites with sampling resolution above 1 Hz (denoted SR1) including 0.5 < α ≤ 1 in the range of persistent fractional Gaussian noise (fGn) (Appendix Figure A2). Within each range, the alpha values were distinct for each of the groups of interest with systematically large separation between TD and ASD; but also, separation between the ASD and AS groups (whenever data for comparison were available). Comparisons between MEDS and No MEDS groups also yielded differences in both types of sampling resolution, thus suggesting that future studies using repositories that report medication intake may benefit from such analyses to determine possible effects of medication on the person's biorhythms (see prior results here, Torres and Denisova, 2016). Further, males and females yielded differences and some degree of overlapping that may be refined using other methods for each of the diagnosis under consideration. Indeed, recent work using these ABIDE sets provided evidence to that end between the females with ASD and females with AS (Torres et al., 2017) that emerged using the micro-movements (fluctuations in peak speed amplitude) data type instead of the raw speed data used here. That work provides scaling and standardization methods that further address the problem of different anatomical sizes in the participants of these databases and the effects such allometric issues have on speed-dependent waveforms. However, discussion of such issues is beyond the scope of this paper. Here, our goal is far more modest than providing a standardized waveform addressing such age-dependent allometric effects. We merely aimed at questioning if different SR could lead to differences in variability with statistically significant distinctions in noise quality.

It is important to note that the biorhythms we extracted from head motion data obtainable from the rs-fMRI time series are merely one out of many possible read-outs from the nervous systems across the body (i.e., the peripheral output that includes motion as a form of kinesthetic reafference, such as breathing and heart rhythms, bodily kinematics and electromyography, among others). Other biorhythms are also reported in various data repositories. As such, the methods presented here may be of use when pooling data harnessed under different sampling resolutions. They include for example electroencephalography (EEG) and other morphological and structural parameters in cross-sectional and longitudinal data, expressible as time series of fluctuations. For a summary on various open access data repositories see (Eickhoff et al., 2016).

Given that analyses involving imaging undergoes a pre-processing stage whereby frames are eliminated based on a threshold derived from head motions, and head motion artifacts are characterized using fluctuations in the amplitude of the linear displacement and/or angular rotations of the head, frame by frame, it may be important for researchers of that community to not assume a theoretical type of random process or follow a “one size fits all” approach. Instead, the results from our analyses suggest that ***empirical estimation*** of different random processes likely underlying the time series under consideration may be more appropriate (as we underscore in Appendix Figure A4). In turn, such empirical estimation (rather than a priori assumption of a theoretical distribution family and/or random process type) particularly when done in a personalized manner, may help other steps preceding the selection of frames to eliminate before other analyses for statistical inference.

In summary, we present evidence that when using large data repositories and pooling data from different fMRI sites, we should be mindful of the underlying instrumentation used to gather the data in the first place. We should also consider the sample sizes and age-compositions of the various groups, and build methods amenable to design standardized scales that we can then map to physical phenomena. In this way, we can initiate the path toward the design of new phenotypic characterizations of the human spectrum to properly associate phenotypic data gathered with subjective and objective means with existing genotypic data, now shared in multiple open-access repositories (Eickhoff et al., 2016).

Acquiring more rigorous scientific practices in the fields that study disorders of the nervous systems may help us reproduce results in open access settings. In turn, this may stimulate the exchange of information worldwide across labs and accelerate the design of target treatments in the future. This work is merely an example (out of many possible methods) of beginning the steps to design research programs within the fields of Psychiatric and Psychology that follow empirical estimation procedures of the scientific method.

## Author contributions

CC: Wrote the methods, analyzed all data and produced the Supplementary Materials; SM: Extracted all head motion data from ABIDE I and ABIDE II, processed all the demographics for exclusion-inclusion criteria and implemented randomizer for bootstrapping; JV: Developed the Python code for DFA; ET: Designed study, created/implemented analyses and wrote paper; CC, SM, JV, and ET: Edited paper. All authors read and approved the last version of the MS.

### Conflict of interest statement

The authors declare that the research was conducted in the absence of any commercial or financial relationships that could be construed as a potential conflict of interest.

## Footnotes

^1^https://afni.nimh.nih.gov/pub/dist/doc/program_help/afni_proc.py.html
