# Supplementary material for: Characterization of Noise Signatures of Involuntary Head Motion in the Autism Brain Imaging Data Exchange Repository
Source: Front Integr Neurosci. 2018 Mar 5;12:7. doi: 10.3389/fnint.2018.00007 (PMC5844956; doi:10.3389/fnint.2018.00007)
Supplement: Supplementary file 1 [file DataSheet1.PDF]

Supplementary Material: Characterization of noise signatures of involuntary head motion in the Autism Brain Imaging Data Exchange repository by Carla Caballero, Sejal Mistry, Joe Vero and Elizabeth B Torres.

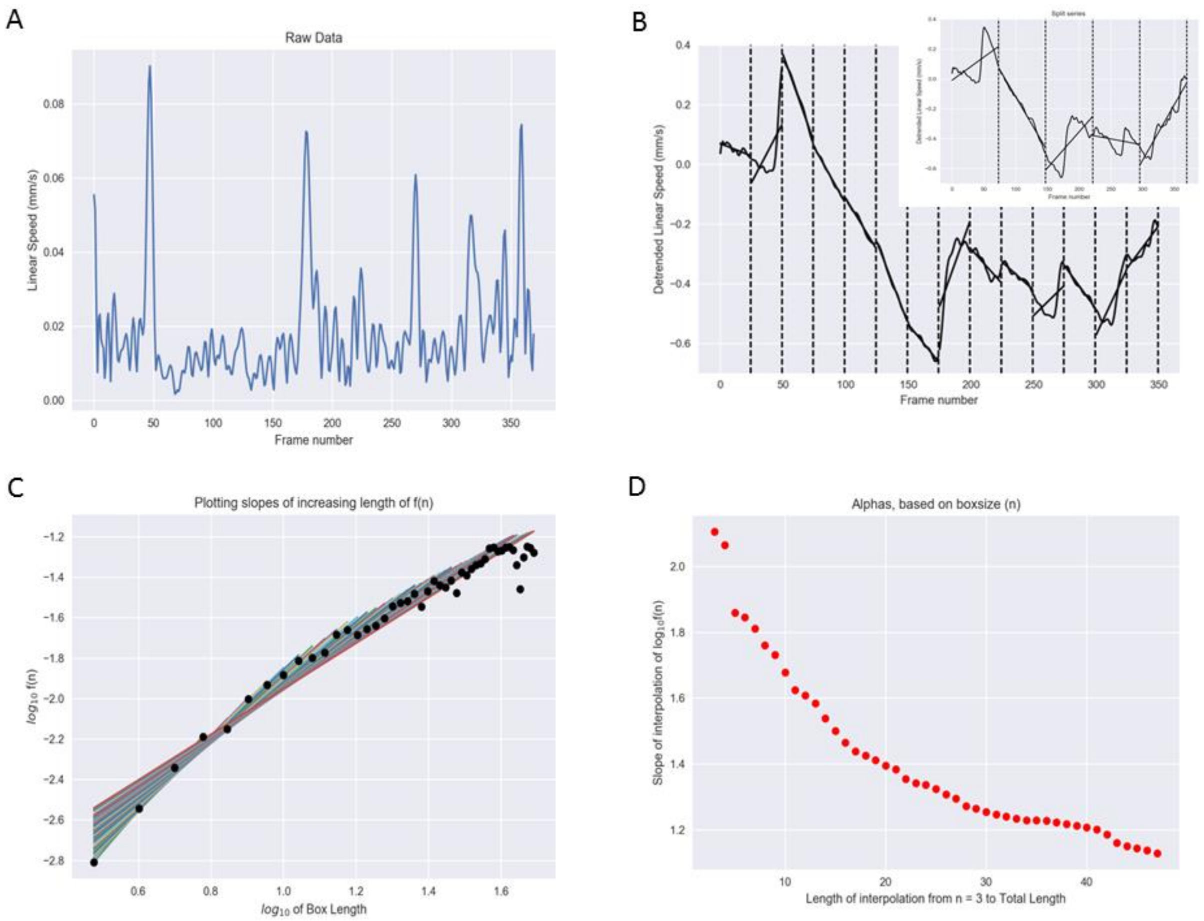

Figures from Python Code.
Raw data input to DFA followed by detrended linear speed using windows of 100 points give rise to the alpha series obtainable from increasing number of points in the series boxing by (3,4,5, …, until all the points are exhausted) and the series of alphas according to the box size.

Legend used along the whole Supplementary material:

TD = Typical development
ASD$_{DSM}$ = Autism spectrum according the Diagnostic and Statistical Manual of Mental Disorders.
AS = Asperger Syndrome
FEM or F = Females
MEDS = On Meds
SR0 = Sampling resolution lower than 1 Hz

ASD = Autism spectrum
ASMIX = combination of participants with a diagnosis of Asperger Syndrome or Pervasive Developmental Disorder not otherwise Specified
M = Males
NoMeds = Off Meds
SR1 = Sampling resolution higher than 1 Hz.

Tables

Table S1. Information regarding the original Sample Resolution (SR) of all the sites in ABIDE I and II and the resampling factors (P and Q) used in each site.

Abide	Site name	N	SR (Hz)	P	Q
**I**	CALTHECH	38	0.5	4	1
**I**	CMU	27	0.5	4	1
**I**	KKI	55	0.4	5	1
**I**	LEUVEN	64	0.6	30	9
**I**	MAX MUN	57	0.33	6	1
**I**	NYU	184	0.5	4	1
**I**	OSHU	28	0.4	5	1
**I**	OLIN	36	0.67	3	1
**I**	PITT	57	0.67	3	1
**I**	SBL	30	0.45	4000	909
**I**	SDSU	36	0.5	4	1
**I**	STANFORD	40	0.5	4	1
**I**	TRINITY	49	0.5	4	1
**I**	UCLA	109	0.33	6	1
**I**	UM	145	0.5	4	1
**I**	USM	101	0.5	4	1
**I**	YALE	56	0.5	4	1
**II**	BNI	58	0.33	6	1
**II**	EMC	54	0.5	4	1
**II**	ETH	37	0.5	4	1
**II**	GU	106	0.5	4	1
**II**	IP	56	0.37	500	93
**II**	IU	40	1.23	200	123
**II**	KKI	211	0.4	5	1
**II**	KUL	28	0.4	5	1
**II**	NYU	105	0.5	4	1
**II**	OHSU	93	0.4	5	1
**II**	ONRC	59	2.11	100	105
**II**	SDSU	58	0.5	4	1
**II**	TCD	42	0.5	4	1
**II**	UCD	32	0.5	4	1
**II**	UCLA	32	0.33	6	1
**II**	USM	33	0.5	4	1

Table S2. Information regarding the sites in ABIDE I and II that contained information in relation to medication intake.

Abide	Site name	N	Columns
**I**	CMU	27	AN-AO
**I**	KKI	55	AN-AO
**I**	LEUVEN 1	29	AN-AO
**I**	MAX MUN	57	AN-AO
**I**	NYU	184	AN-AO
**I**	OLIN	36	AN-AO
**I**	PITT	57	AN-AO
**I**	SBL	30	AN-AO
**I**	SDSU	36	AN-AO
**I**	STANFORD	40	AN-AO
**I**	TRINITY	49	AN
**I**	UCLA 1	82	AN-AO
**I**	UCLA 2	27	AN-AO
**I**	UM 1	110	AN-AO
**I**	UM 2	35	AN-AO
**II**	BNI	58	BB-BC
**II**	EMC	54	BB-BC
**II**	GU	106	BB-BC
**II**	IP	56	BB-BC
**II**	KKI	211	BB-BC
**II**	KUL	28	BB-BC
**II**	NYU 1	78	BB-BC
**II**	NYU 2	27	BB-BC
**II**	OHSU	93	BB-BC
**II**	ONRC	59	BB-BC
**II**	SDSU	58	BB-BC
**II**	TCD	42	BB-BC
**II**	UCD	32	BB-BC
**II**	UCLA	32	BB-BC
**II**	USM	33	BB-BC

Table S3. Information about the number of participants (N) of each group according their original sample resolution, diagnosis, sex and meds status.

		N All sites	N SR0	SR1
**Diagnosis**	ASD	1026	981	44
	TD	1130	1074	55
	ASD_{DSM}	456	456	0
	AS	189	189	0
	ASMIX	298	298	0
**Sex**	FEMALES	412	383	28
	MALES	1744	1672	71
**Meds Status**	MEDS	291	284	7
	NoMEDS	1466	1413	52
**Diagnosis + Sex**	FEM ASD	138	130	8
	FEM TD	273	252	20
	FEM ASD_{DSM}	55	55	0
	FEM AS	29	29	0
	FEM ASMIX	40	40	0
	MALES ASD	888	851	36
	MALES TD	856	821	35
	MALES ASD_{DSM}	401	401	0
	MALES AS	160	160	0
	MALES ASMIX	258	258	0
**Sex + Meds Status**	FEM MEDS	44	42	2
	FEM NoMEDS	313	295	17
	MALES MEDS	248	242	5
	MALES NoMEDS	1153	1118	35
**Diagnosis + Meds Status**	ASD MEDS	269	261	7
	ASD NoMEDS	578	561	17
	ASD_{DSM} MEDS	128	128	0
	ASD_{DSM} NoMEDS	231	231	0
	AS MEDS	52	52	0
	AS NoMEDS	128	128	0
	ASMIX MEDS	72	72	0
	ASMIX NoMEDS	196	196	0
	TD MEDS	23	23	0
	TD NoMEDS	888	853	35
**Diagnosis + Sex + Meds Status**	FEM ASD MEDS	37	35	2
	FEM ASD NoMEDS	78	76	2
	FEM ASD_{DSM} MEDS	7	7	0
	FEM ASD_{DSM} NoMEDS	39	39	0
	FEM AS MEDS	16	16	0
	FEM AS NoMEDS	11	11	0
	FEM ASMIX MEDS	17	17	0
	FEM ASMIX NoMEDS	16	16	0
	FEM TD MEDS	7	7	0
	FEM TD NoMEDS	235	220	15
	MALES ASD MEDS	232	226	5
	MALES ASD NoMEDS	500	485	15
	MALES ASD_{DSM} MEDS	121	121	0
	MALES ASD_{DSM} NoMEDS	192	192	0
	MALES AS MEDS	36	36	0
	MALES AS NoMEDS	117	117	0
	MALES ASMIX MEDS	55	55	0
	MALES ASMIX NoMEDS	180	180	0
	MALES TD MEDS	16	16	0
	MALES TD NoMEDS	653	633	20

The groups with red font are those that we did not use to do any comparisons because they do not have enough number of participants (N < 20).

Table S4. Information regarding the statistics for the comparisons of the α values computed over the Linear speed data from the sites in ABIDE I and II that have original SR higher than 1 Hz versus those that have original SR lower than 1 Hz.

	**SR1**		**SR0**		**Mean p-value**	**Mode p-value**
ASD	Mean	Variance	Mean	Variance	< 0,001	< 0,001
	1.1910	0.0637	1.6816	2.30e-04		
TD	Mean	Variance	Mean	Variance	< 0,001	< 0,001
	1.1052	0.0996	1.6740	2.3644		
FEMALES	Mean	Variance	Mean	Variance	< 0,001	< 0,001
	1.1160	0.0567	1.6965	1.07e-04		
MALES	Mean	Variance	Mean	Variance	< 0,001	< 0,001
	1.1541	0.0995	1.6643	1.94e-04		
TD FEMALES	Mean	Variance	Mean	Variance	< 0,001	< 0,001
	1.0781	0.0586	1.6997	2.50e-04		
TD MALES	Mean	Variance	Mean	Variance	< 0,001	< 0,001
	1.1206	0.1240	1.6672	4.23e-04		
ASD MALES	Mean	Variance	Mean	Variance	< 0,001	< 0,001
	1.1866	0.0698	1.6765	2.93e-04		

Table S5. Information regarding the statistics for the comparisons of the α values computed over the Angular speed data from the sites in ABIDE I and II that have original SR1 versus those that have original SR0.

	**SR1**		**SR0**		**Mean p-value**	**Mode p-value**
ASD	Mean	Variance	Mean	Variance	< 0,001	< 0,001
	1.0788	0.0512	1.6084	2.58e-04		
TD	Mean	Variance	Mean	Variance	< 0,001	< 0,001
	0.9812	0.787	1.5895	2.87e-04		
FEMALES	Mean	Variance	Mean	Variance	< 0,001	< 0,001
	1.0082	0.0565	1.5779	1.30e-04		
MALES	Mean	Variance	Mean	Variance	< 0,001	< 0,001
	1.0310	0.0762	1.5877	2.54e-04		
TD FEMALES	Mean	Variance	Mean	Variance	< 0,001	< 0,001
	0.9674	0.0564	1.5710	2.31e-04		
TD MALES	Mean	Variance	Mean	Variance	< 0,001	< 0,001
	0.9891	0.0917	1.5910	4.91e-04		
ASD MALES	Mean	Variance	Mean	Variance	< 0,001	< 0,001
	1.0718	0.0539	1.6074	3.29		

Table S6. Information regarding the statistics for the comparisons of the α values computed over the linear and angular speed data for the main groups according to diagnosis, sex and meds status criteria from all the different groups from the sites in ABIDE I and II that have original SR0**.**

DIAGNOSIS COMPARISONS

	Linear speed		***Angular Speed***	
	Mean ± Variance	*Mean ± Variance*	Mean ± Variance	*Mean ± Variance*
ASDvs*TD*	1.7016±0.0119	*1.7066±0.0138*	1.6481±0.0173	*1.6410±0.0185*
ASD$_{DSM}$vs*TD*	1.6993±0.0495	*1.6990±1.26e-05*	1.6458±0.0620	*1.6313±1.74e-05*
ASvs*TD*	1.7318±0.0418	*1.7044±4.71e-05*	1.6626±0.0570	*1.6396±6.08e-05*
ASMIXvs*TD*	1.7170±0.0418	*1.7098±2.46e-05*	1.6494±0.0591	*1.6431±3.45e-05*
ASvs*ASD$_{DSM}$*	1.7318±0.0418	*1.7000±3.06e-05*	1.6626±0.0570	*1.6409±4.66e-05*
ASMIXvs*ASD$_{DSM}$*	1.7170±0.0419	*1.6998±1.65e-05*	1.6494±0.0591	*1.6429±2.24e-05*
SEX COMPARISON				
	Linear speed		***Angular Speed***	
	Mean ± Variance	*Mean ± Variance*	Mean ± Variance	*Mean ± Variance*
FEMALESvs*MALES*	1.7149±0.0454	*1.7108±1.94e-05*	1.6589±0.0593	*1.6532±2.48e-05*
MEDS STATUS COMPARISON				
	Linear speed		***Angular Speed***	
	Mean ± Variance	*Mean ± Variance*	Mean ± Variance	*Mean ± Variance*
MEDvs*NoMEDS*	1.7030±0.0492	*1.7133±3.11e-05*	1.6614±0.0573	*1.6567±3.90e-05*

The columns with *italic* font correspond to the second group in the comparison, also in with *italic* font.

Table S7. Information regarding the statistics for the comparisons of the α values computed over the linear and angular speed data for the main groups according to the combination of diagnosis and sex criteria from all the different groups from the sites in ABIDE I and II that have original SR0**.**

DIAGNOSIS + SEX COMPARISONS

		Linear speed		***Angular Speed***	
		Mean ± Variance	*Mean ± Variance*	Mean ± Variance	*Mean ± Variance*
**FEMALES**	ASDvs*TD*	1.7071±0.0473	*1.7248±2.22e-05*	1.6711±0.0586	*1.6487±3.09e-05*
	ASD$_{DSM}$vs*TD*	1.7031±0.0500	*1.7010±1.10e-05*	1.6572±0.0657	*1.6257±1.68e-04*
	ASvs*TD*	1.7586±0.0430	*1.7268±2.34e-04*	1.7301±0.0492	*1.6724±3.35e-04*
	ASMIXvs*TD*	1.7392±0.0435	*1.7223±1.30e-04*	1.7058±0.0554	*1.6683±1.98e-05*
	ASvs*ASD$_{DSM}$*	1.7486±0.0430	*1.6842±1.30e-04*	1.7301±0.0492	*1.6451±2.79e-04*
	ASMIXvs*ASD$_{DSM}$*	1.7392±0.0112	*1.7031±0.0140*	1.7058±0.0182	*1.6572±0.0182*
**MALES**	TDvs*ASD*	1.6773±0.0431	*1.6798±0.0272*	1.6374±0.0188	*1.6445±0.0172*
	ASD$_{DSM}$vs*TD*	1.6988±0.0494	*1.6989±1.22e-05*	1.6442±0.0622	*1.6321±1.62e-05*
	ASvs*TD*	1.7269±0.0414	*1.7000±5.40e-05*	1.6504±0.0573	*1.6367±7.30e-08*
	ASMIXvs*TD*	1.7136±0.0416	*1.7066±2.29e-05*	1.6406±0.0590	*1.6406±3.21e-05*
	ASvs*ASD$_{DSM}$*	1.7269±0.0414	*1.6937±4.17e-05*	1.6504±0.0573	*1.6376±6.28e-05*
	ASMIXvs*ASD$_{DSM}$*	1.7136±0.0416	*1.6987±1.92e-05*	1.6406±0.0590	*1.6404±3.18e-05*
**FEMALES vs *MALES***	ASD	1.7071±0.0473	*1.7034±6.48e-05*	1.6711±0.0586	*1.6497±9.76-05*
	ASD$_{DSM}$	1.7031±0.0500	*1.6988±1.54e-04*	1.6572±0.0605	*1.6489±2.20e-04*
	AS	1.7586±0.0430	*1.7522±1.27e-04*	1.7301±0.0492	*1.6777±2.43e-04*
	ASMIX	1.7392±0.0435	*1.7221±1.20e-04*	1.7058±0.0554	*1.6539±2.32e-04*
	TD	1.7189±0.0443	*1.7166±2.92e-05*	1.6527±0.0596	*1.6548±3.36e-05*

The columns with *italic* font correspond to the second group in the comparison, also in with *italic* font.

Table S8. Information regarding the statistics for the comparisons of the α values computed over the linear and angular speed data for the main groups according to the combination of diagnosis and meds status criteria from all the different groups from the sites in ABIDE I and II that have original SR0**.**

DIAGNOSIS + MEDS STATUS COMPARISONS

	Linear speed		***Angular Speed***	
	Mean ± Variance	Mean ± Variance	Mean ± Variance	Mean ± Variance
Fem MEDSvs*NoMEDS*	1.7243±0.0443	*1.7087±2.02e-04*	1.6900±0.0538	*1.6586±2.60e-04*
Males MEDSvs*NoMEDS*	1.6993±0.0499	*1.7120±1.6938*	1.6565±0.0578	*1.6550±4.21e-05*
MEDS Femvs*Males*	1.7243±0.0443	*1.7141±1.64e-04*	1.6900±0.0538	*1.6610±2.08e-04*
NoMEDS Femvs*Males*	1.7184±0.0463	*1.7188±2.56e-05*	1.6672±0.0587	*1.6629±3.46e-05*
ASD MEDSvs*NoMEDS*	1.7024±0.0497	*1.7109±1.69e-05*	1.6614±0.0582	*1.6595±1.43e-05*
ASD_{DSM} MEDSvs*NoMEDS*	1.7182±0.0497	*1.7038±4.69e-05*	1.6725±0.0583	*1.6611±6.03e-05*
AS MEDSvs*NoMEDS*	1.7257±0.0403	*1.7355±6.21e-05*	1.6813±0.0517	*1.6852±9.88e-05*
ASMIX MEDSvs*NoMEDS*	1.7134±0.0427	*1.7185±5.06e-05*	1.6667±0.0559	*1.6640±1.6405*
MEDS ASvsASD_{DSM}	1.7257±0.0403	*1.7304±7.28e-05*	1.6813±0.0517	*1.6814±1.09e-04*
MEDS ASMIXvsASD_{DSM}	1.7134±0.0427	*1.7321±4.78e-05*	1.6667±0.0559	*1.6784±9.06e05*
NoMEDS ASvsASD_{DSM}	1.7355±0.0428	*1.6912±7.39e-05*	1.6582±0.0593	*1.6406±8.92e-05*
NoMEDS ASMIXvsASD_{DMS}	1.7199±0.0430	*1.6969±3.12e-05*	1.6523±0.0581	*1.6494±4.34e-05*
NoMEDS ASDvs*TD*	1.7068±0.0460	*1.7108±5.98e-06*	1.6540±0.0588	*1.6508±8.32e-06*
NoMEDS ASD_{DSM}vs*TD*	1.7018±0.0486	*1.7068±3.51e-05*	1.6496±0.0632	*1.6461±4.20e-05*
NoMEDS ASvs*TD*	1.7355±0.0428	*1.7065±9.13e-05*	1.6582±0.0593	*1.6418±1.13e-04*
NoMEDS ASMIXvs*TD*	1.7199±0.0430	*1.7119±3.63e-05*	1.6523±0.0581	*1.6476±5.09e-05*

The columns with *italic* font correspond to the second group in the comparison, also in with *italic* font.

Table S9. Information regarding the statistics for the comparisons of the α values computed over the linear and angular speed data for the main groups according to the combination of diagnosis, sex and meds status criteria from all the different groups from the sites in ABIDE I and II that have original SR0.

DIAGNOSIS + SEX + MEDS STATUS COMPARISONS

	Linear speed		***Angular Speed***	
	Mean ± Variance	*Mean ± Variance*	Mean ± Variance	*Mean ± Variance*
F ASD MEDSvs*NoMEDS*	1.7344±0.0443	*1.6989±9.25e-05*	1.6978±0.0560	*1.6735±1.64e-04*
M ASD MEDSvs*NoMEDS*	1.6975±0.0504	*1.7111±2.00e-08*	1.6558±0.0583	*1.6554±2.44e-05*
M ASD_{DSM} MEDSvs*NoMEDS*	1.7167±0.0504	*1.7090±3.62e-05*	1.6690±0.0590	*1.6684±5.16e-05*
M AS MEDSvs*NoMEDS*	1.7207±0.0375	*1.7376±1.38e-04*	1.6630±0.0512	*1.6778±2.30e-04*
M ASMIX MEDSvs*NoMEDS*	1.7054±0.0415	*1.7183±9.18e-05*	1.6518±0.0557	*1.6549±1.45e-04*
ASD MEDS Femvs*Males*	1.7344±0.0443	*1.7083±2.21e-04*	1.6978±0.0560	*1.6582±2.99e-04*
ASD NoMEDS Femvs*Males*	1.7031±0.0496	*1.7078±1.08e-04*	1.6788±0.0583	*1.6549±1.29e-04*
ASD_{DSM} NoMEDS Femvs*Males*	1.7046±0.0512	*1.7019±1.97e-04*	1.6516±0.0658	*1.6641±2.58e-04*
F NoMEDS ASDvs*TD*	1.7031±0.0496	*1.7164±6.09e-05*	1.6788±0.0583	*1.6486±8.56e-05*
F NoMEDS ASD_{DSM}vs*TD*	1.7046±0.0512	*1.7160±1.85e-04*	1.6516±0.0658	*1.6465±2.61e-04*
M NoMEDS ASDvs*TD*	1.7073±0.0454	*1.7048±1.03e-05*	1.6501±0.0588	*1.6430±1.47e-05*
M NoMEDS ASD_{DSM}vs*TD*	1.7012±0.0480	*1.7020±3.11e-05*	1.6492±0.0626	*1.6389±4.17e-05*
M NoMEDS ASvs*TD*	1.7295±0.0430	*1.6962±8.43e-05*	1.6474±0.0597	*1.6319±1.12e-04*
M NoMEDS ASMIXvs*TD*	1.7166±0.0426	*1.7035±4.11e-05*	1.6429±0.0586	*1.6373±5.58e-05*
M MEDS ASvsASD_{DSM}	1.7207±0.0375	*1.7258±1.47e-04*	1.6630±0.0512	*1.6712±2.05e-04*
M MEDS ASMIXvsASD_{DSM}	1.7054±0.0415	*1.7269±8.44e-05*	1.6518±0.0557	*1.6790±1.8e-04*
M NoMEDS ASvsASD_{DSM}	1.7295±0.0430	*1.6875±4.84e-05*	1.6474±0.0597	*1.6375±6.87e-05*
M NoMEDS ASMIXvsASD_{DSM}	1.7166±0.0426	*1.6961±2.76e-05*	1.6429±0.0586	*1.6491±4.03e-05*

The columns with *italic* font correspond to the second group in the comparison, also in with *italic* font.

Table S10. Information regarding the statistics for all the comparisons of the α values computed over the linear and angular speed data from all the different groups from the sites in ABIDE I and II with original SR1.

LINEAR SPEED						
ASD vs TD	**Mean**	**Variance**	**Mean**	**Variance**	**p-value**	
	1.1910	0.337	1.1052	0.778	0.2763	
FEMALES vs MALES	Mean	Variance	Mean	Variance	Mean p-value	Mode p-value
	1.1160	0.0567	1.1860	8.46e-04	5.21e-06	3.48e-12
TD Fem vs Males	Mean	Variance	Mean	Variance	Mean p-value	Mode p-value
	1.0781	0.0586	1.1212	0.0015	0.0010	2.14e-05
Males ASD vs TD	Mean	Variance	Mean	Variance	p-value	
	1.1202	0.0991	1.1866	0.0393	0.5626	
ANGULAR SPEED						
ASD vs TD	**Mean**	**Variance**	**Mean**	**Variance**	**p-value**	
	1.0788	0.0306	0.9812	0.0623	0.1240	
FEMALES vs MALES	Mean	Variance	Mean	Variance	Mean p-value	Mode p-value
	1.0082	0.0565	1.0500	6.60e-04	0.0046	5.79e-09
TD Fem vs Males	Mean	Variance	Mean	Variance	Mean p-value	Mode p-value
	0.9674	0.0564	0.9972	0.0012	8.66e-04	2.34e-09
Males ASD vs TD	Mean	Variance	Mean	Variance	p-value	
	1.0718	0.0341	0.9891	0.0745	0.2251	

NOTE: The comparisons that don't have mean and mode of the p-values are which are done without using Bootstrapping method because the groups had similar sizes.

Table S11. Mean ± Variance of the alpha values distributions for each group before applying the *Bootstrapping method*, i.e. using the groups according to the main criteria and their combinations.

Groups	N SR < 1Hz		N SR > 1Hz	
	LS	AS	LS	AS
	Mean±Variance	Mean±Variance	Mean±Variance	Mean±Variance
TD	1.7066±0.0479	1.6410±0.0615	1.1052±0.0996	0.9812±0.0787
ASD	1.7016±0.0469	1.6481±0.0596	1.1910±0.0637	1.0788±0.0512
ASD_{DSM}	1.6993±0.0495	1.6458±0.0620	----	----
AS	1.7318±0.0418	1.6626±0.0570	----	----
ASMIX	1.7170±0.0419	1.6494±0.0591	----	----
MEDS	1.7030±0.0492	1.6614±0.0573	1.1236±0.0682	0.9902±0.0515
NoMEDS	1.7113±0.0473	1.5432±0.0602	1.0271±0.0711	0.9274±0.0604
FEMALES	1.7149±0.0454	1.6589±0.0593	1.1160±0.0567	1.0082±0.0565
MALES	1.7018±0.0479	1.6411±0.608	1.1541±0.0995	1.0310±0.0762
TD FEM	1.7189±0.0443	1.6527±0.0596	1.0781±0.0586	0.9674±0.0564
TD MALES	1.7029±0.0490	1.6374±0.0620	1.1240±0.1007	0.9891±0.0917
ASD FEM	1.7071±0.0473	1.6711±0.0586	1.2107±0.0346	1.1103±0.0372
ASD MALES	1.7007±0.0468	1.6445±0.0596	1.1866±0.0698	1.0718±0.0539
ASD_{DSM} FEM	1.7031±0.0500	1.6572±0.0605	----	----
ASD_{DSM} MALES	1.6977±0.0494	1.6442±0.0622	----	----
AS FEM	1.7586±0.0430	1.7301±0.0492	----	----
AS MALES	1.7269±0.0414	1.6504±0.0573	----	----
ASMIX FEM	1.7392±0.0435	1.7058±0.0554	----	----
ASMIX MALES	1.7136±0.0416	1.6406±0.0590	----	----
MEDS FEM	1.7243±0.0443	1.6900±0.0538	1.2439±0.0083	1.2223±0.0142
MEDS MALES	1.6993±0.0499	1.6565±0.0578	1.0755±0.0777	0.8974±0.0335
NoMEDS FEM	1.7184±0.0463	1.6672±0.0587	1.0403±0.0521	0.9372±0.0550
NoMEDS MALES	1.7094±0.0476	1.6495±0.0605	1.0208±0.0798	0.9226±0.0628
ASD MEDS	1.7024±0.0497	1.6614±0.0582	1.1236±0.0682	0.9902±0.0515
ASD NoMEDS	1.7068±0.0460	1.6540±0.0588	1.0829±0.0293	1.0176±0.0351
ASD_{DSM} MEDS	1.7182±0.0497	1.6725±0.0583	----	----
ASD_{DSM} NoMEDS	1.7018±0.0486	1.6496±0.0632	----	----
AS MEDS	1.7257±0.0403	1.6813±0.0517	----	----
AS NoMEDS	1.7355±0.0428	1.6582±0.0593	----	----
ASMIX MEDS	1.7134±0.0427	1.6667±0.0559	----	----
ASMIX NoMEDS	1.7199±0.0430	1.6523±0.0581	----	----
FEM ASD MEDS	1.7344±0.0443	1.6978±0.0560	1.2439±0.0083	1.2223±0.0142
FEM ASD NoMEDS	1.7031±0.0496	1.6788±0.0583	1.1263±0.0294	1.0222±0.0366
FEM ASD_{DSM} MEDS	1.7446±0.0368	1.7321±0.0400	----	----
FEM ASD_{DSM} NoMEDS	1.7046±0.0512	1.6516±0.0658	----	----
FEM AS MEDS	1.7370±0.0466	1.7225±0.0502	----	----
FEM AS NoMEDS	1.7988±0.0361	1.7724±0.0374	----	----
FEM ASMIX MEDS	1.7395±0.0455	1.7149±0.0530	----	----
FEM ASMIX NoMEDS	1.7579±0.0459	1.7581±0.0374	----	----
MALES ASD MEDS	1.6975±0.0504	1.6558±0.0583	1.0755±0.0777	0.8974±0.0335
MALES ASD NoMEDS	1.7073±0.0454	1.5401±0.0588	1.0772±0.0290	1.0170±0.0349
MALES ASD_{DSM} MEDS	1.7167±0.0405	1.6690±0.0590	----	----
MALES ASD_{DSM} NoMEDS	1.7012±0.0480	1.6492±0.0626	----	----
MALES AS MEDS	1.7207±0.0375	1.6630±0.0512	----	----
MALES AS NoMEDS	1.7295±0.0430	1.6474±0.0597	----	----
MALES ASMIX MEDS	1.7054±0.0415	1.6518±0.0557	----	----
MALES ASMIX NoMEDS	1.7166±0.0426	1.6429±0.0586	----	----

The groups with red font are those that we did not use to do any comparisons because they do not have enough number of participants (N < 20).

Figures

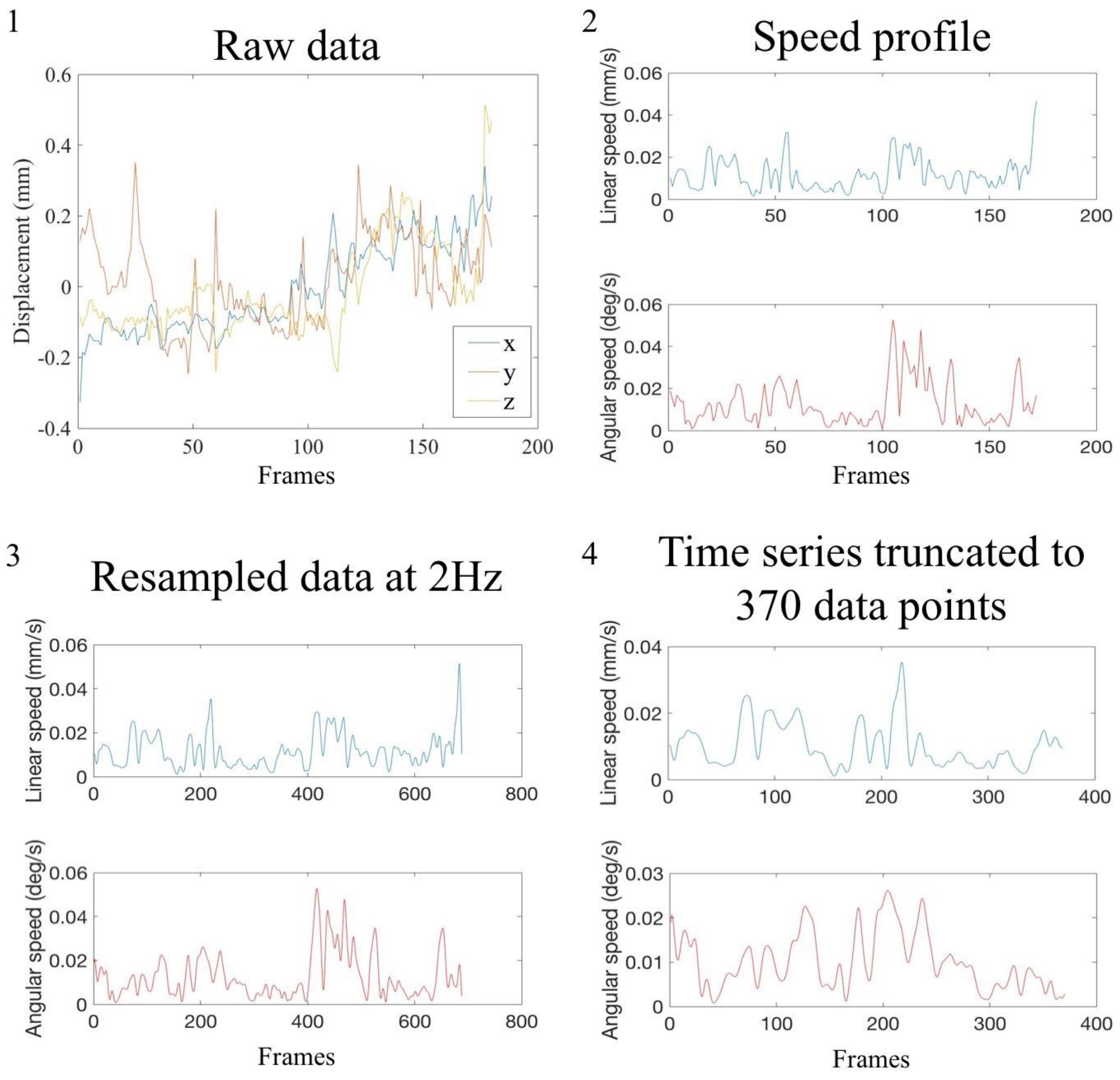

Figure S1: Processing of kinematic data. 1) Obtain raw positional data (the example shown is the linear displacement, but we extracted both linear displacements and angular rotations); 2) Velocity obtained from positional data is computed and the scalar value (speed) obtained, both linear and angular speed; 3) The raw data is resampled to ensure equally spaced points for comparison across subjects and groups. All the datasets were resampled at 2 Hz; 4) Resampled data in then truncated to ensure the same length for all the data. In this case, we shortened the time series by 370 data point because it was the shortest length.

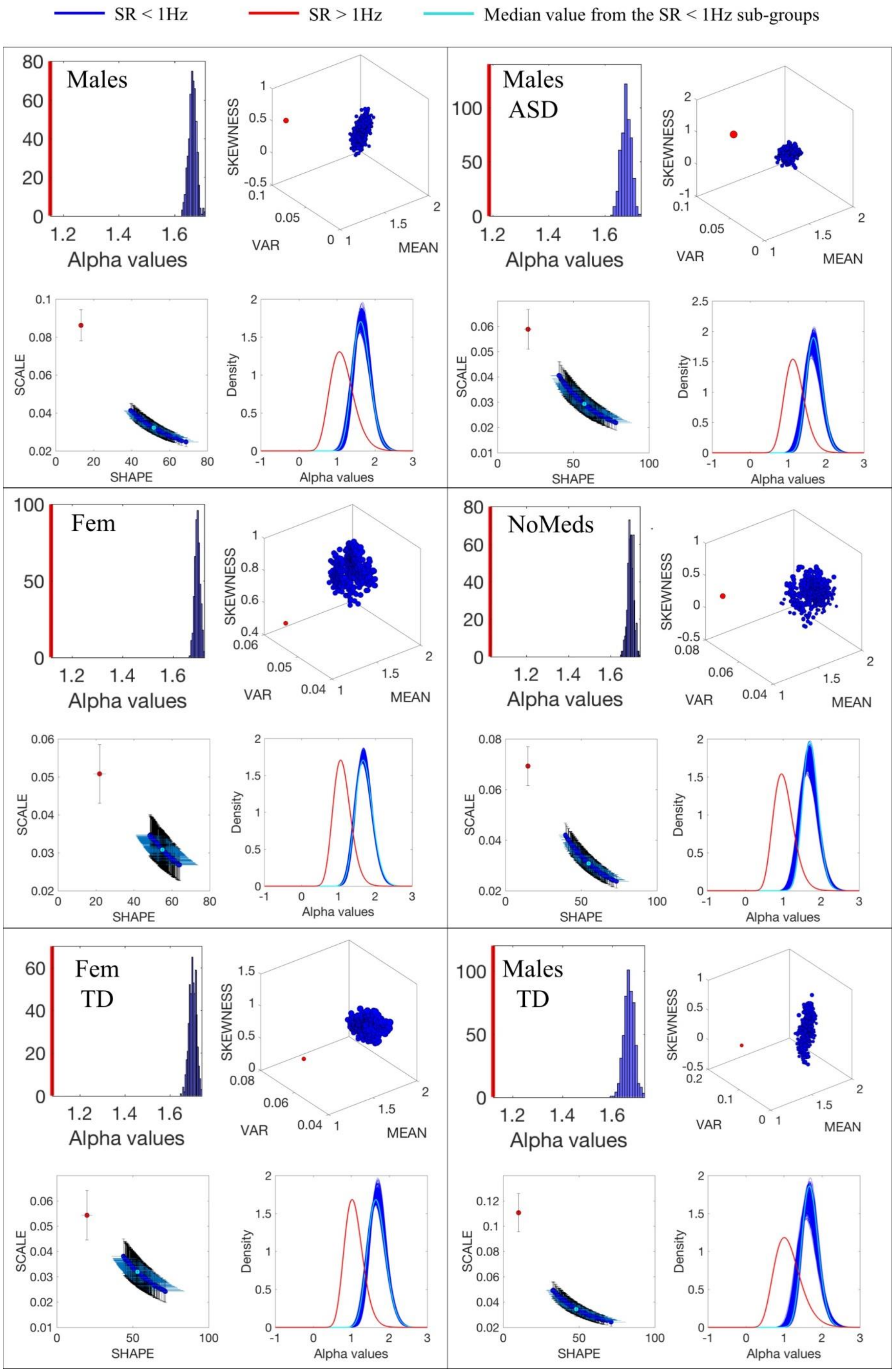

Figure S2. Comparison between the distributions of α values of the Linear Speed for the groups with different original SR. Figure format like Figure 6 from the main manuscript.

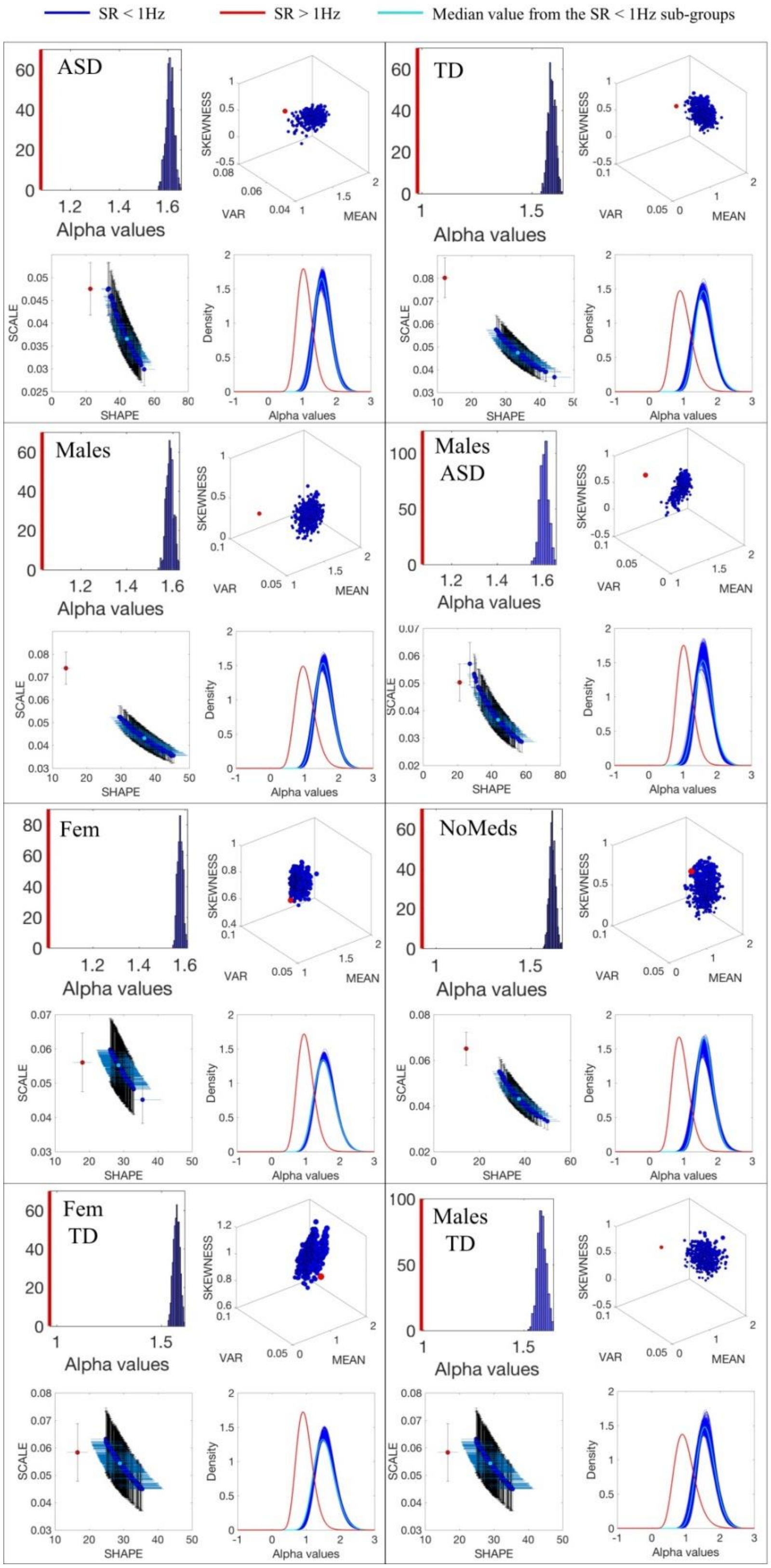

Figure S3. Comparison between the distributions of α values of the Angular Speed for the groups with different original SR. Figure format like Figure 6 from the main manuscript.

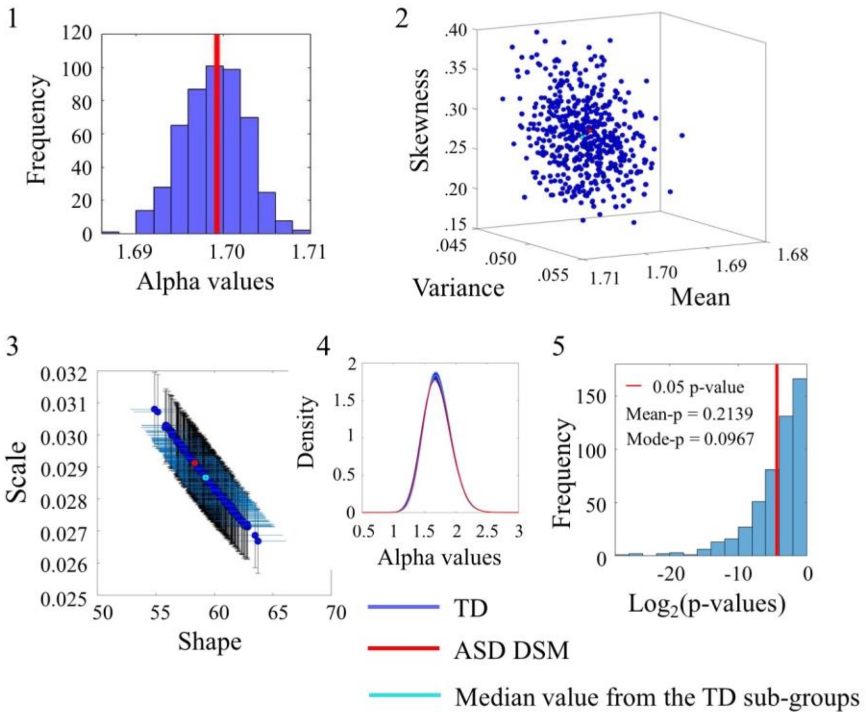

Figure S4. Comparison of the α values between **TD and ASD$_{\mathbf{DSM}}$** groups for the Linear Speed (SR0 groups). Figure format like Figures 7-11 from the main manuscript.

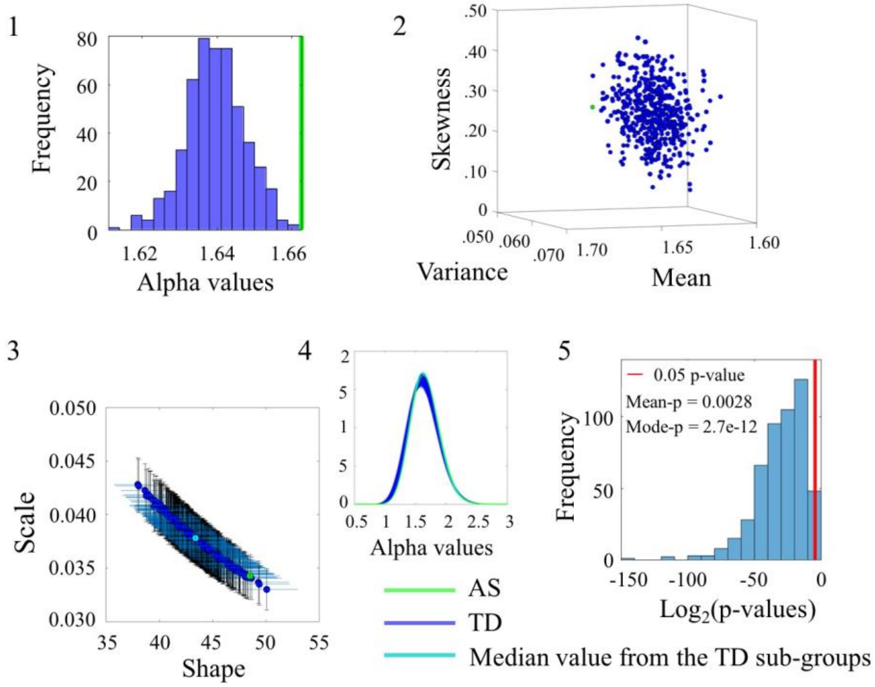

Figure S5. Comparison of the α values between TD and AS groups for the Angular Speed (SR0 groups). Figure format similar to Figures 7-11 from the main manuscript.

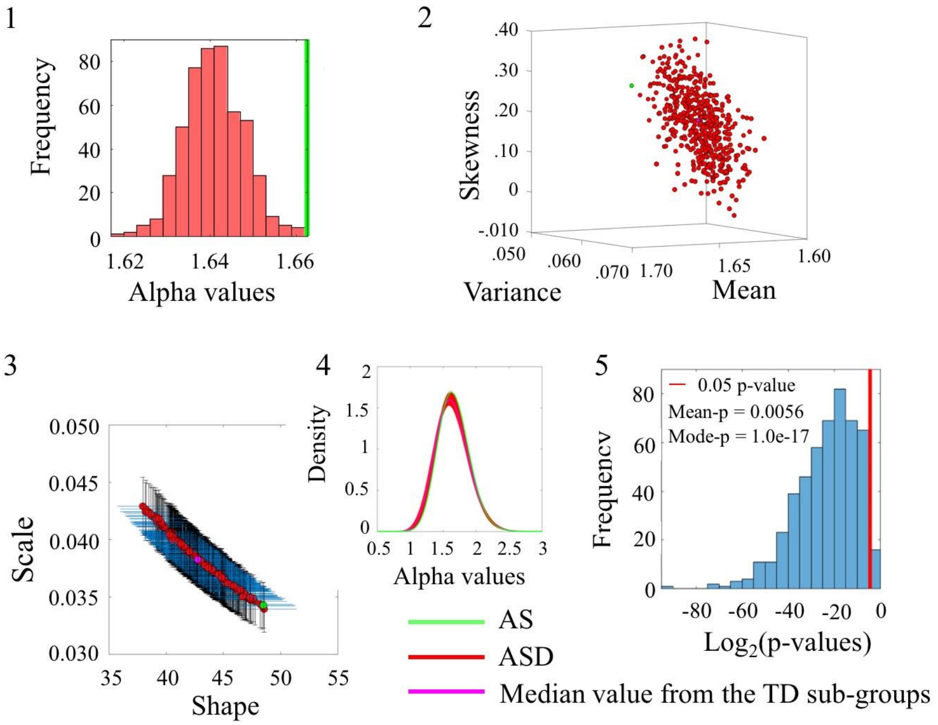

Figure S6. Comparison of the α values between ASD_{DSM} and AS groups for the Angular Speed (SR0 groups). Figure format similar to Figures 7-11 from the main manuscript.

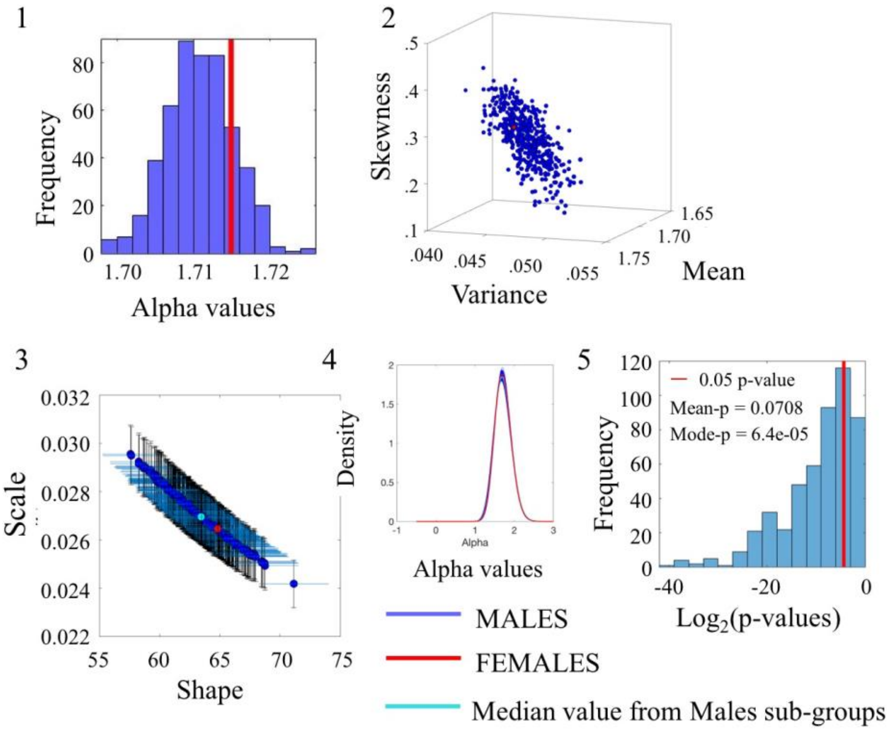

Figure S7. Comparison of the α values between Females and Males groups for the Linear Speed (SR0 groups). Figure format similar to Figures 7-11 from the main manuscript.

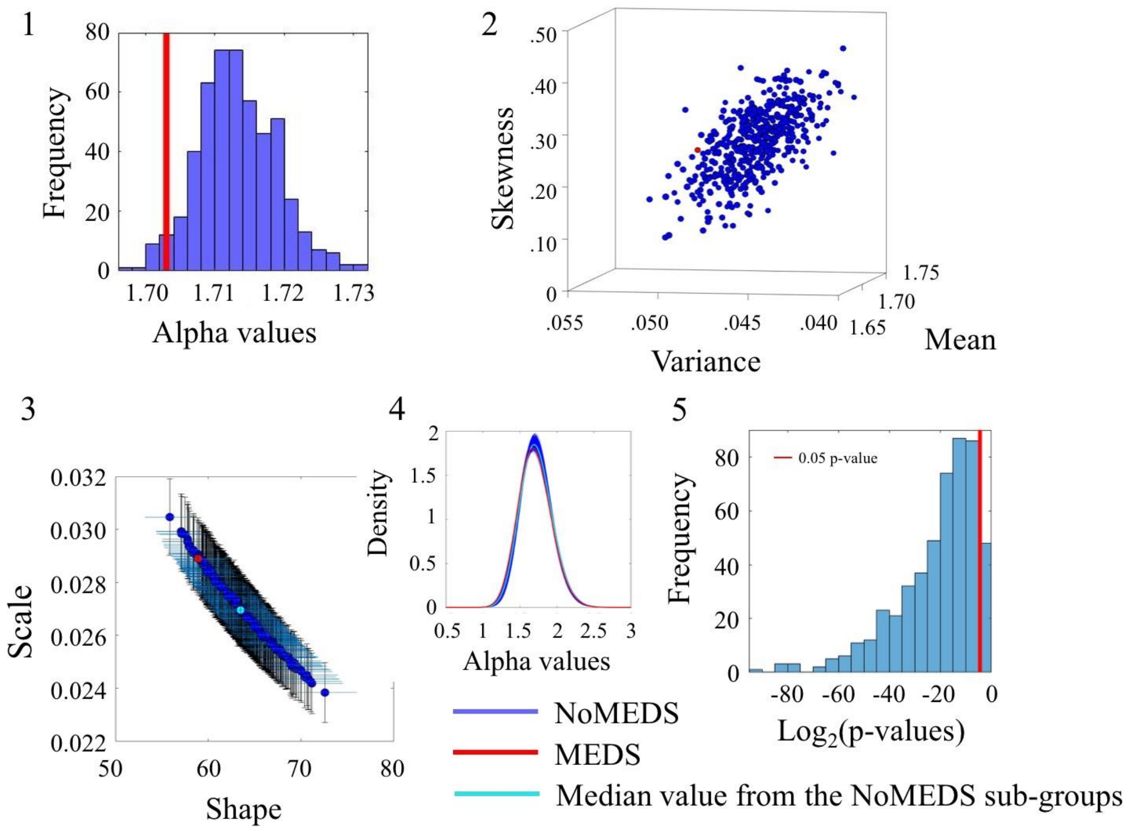

Figure S8. Comparison of the α values between Meds and NoMeds groups for the Linear Speed (SR0 groups). Figure format similar to Figures 7-11 from the main manuscript.

Figures S9-S16 display the distributions of the α values (mean) for the 500 sub-groups extracted from the original large groups for each comparison, with similar size and age composition as the corresponding small groups. The red vertical line represents the mean of the α values of the small group for each comparison.

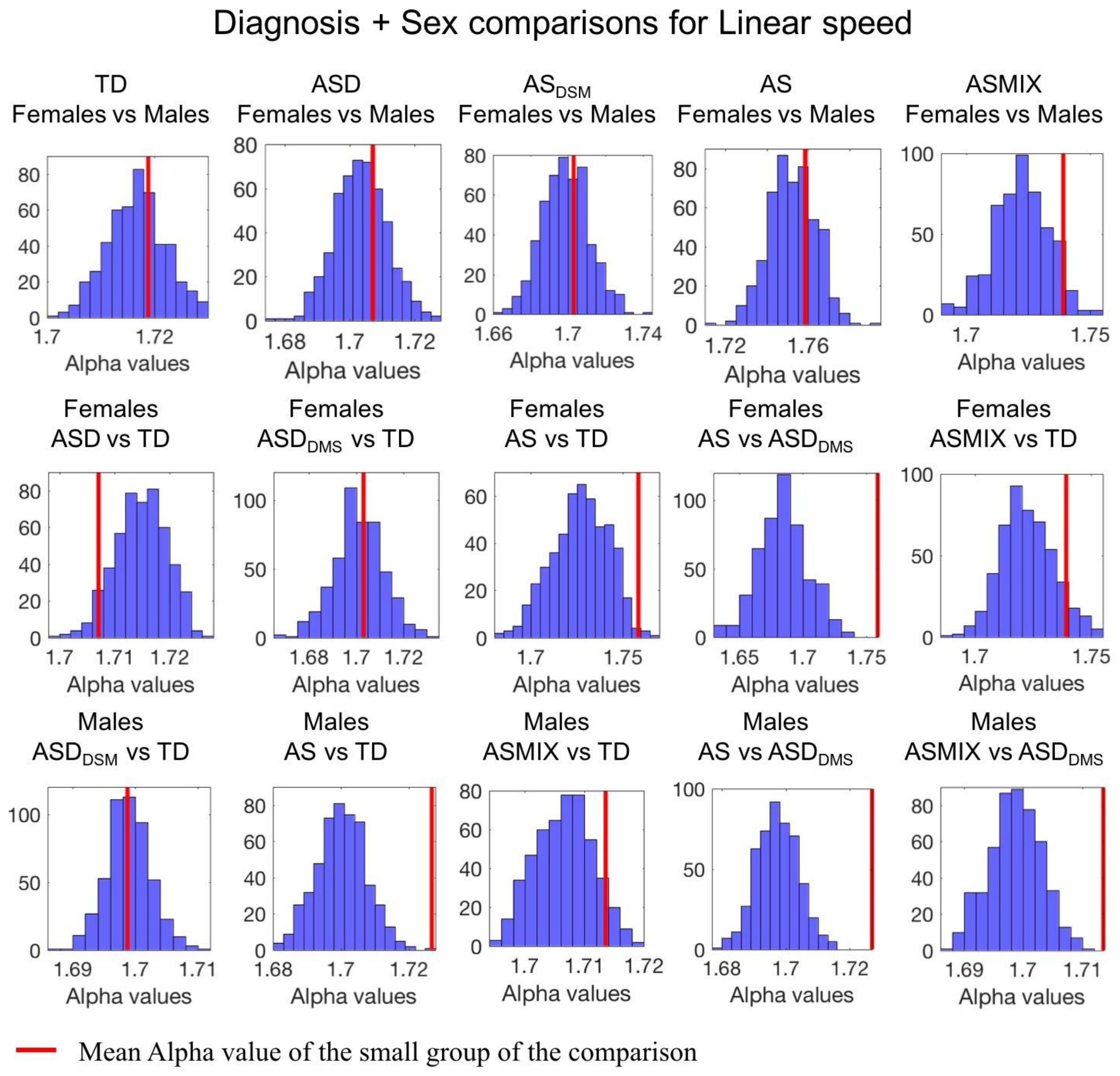

Figure S9.

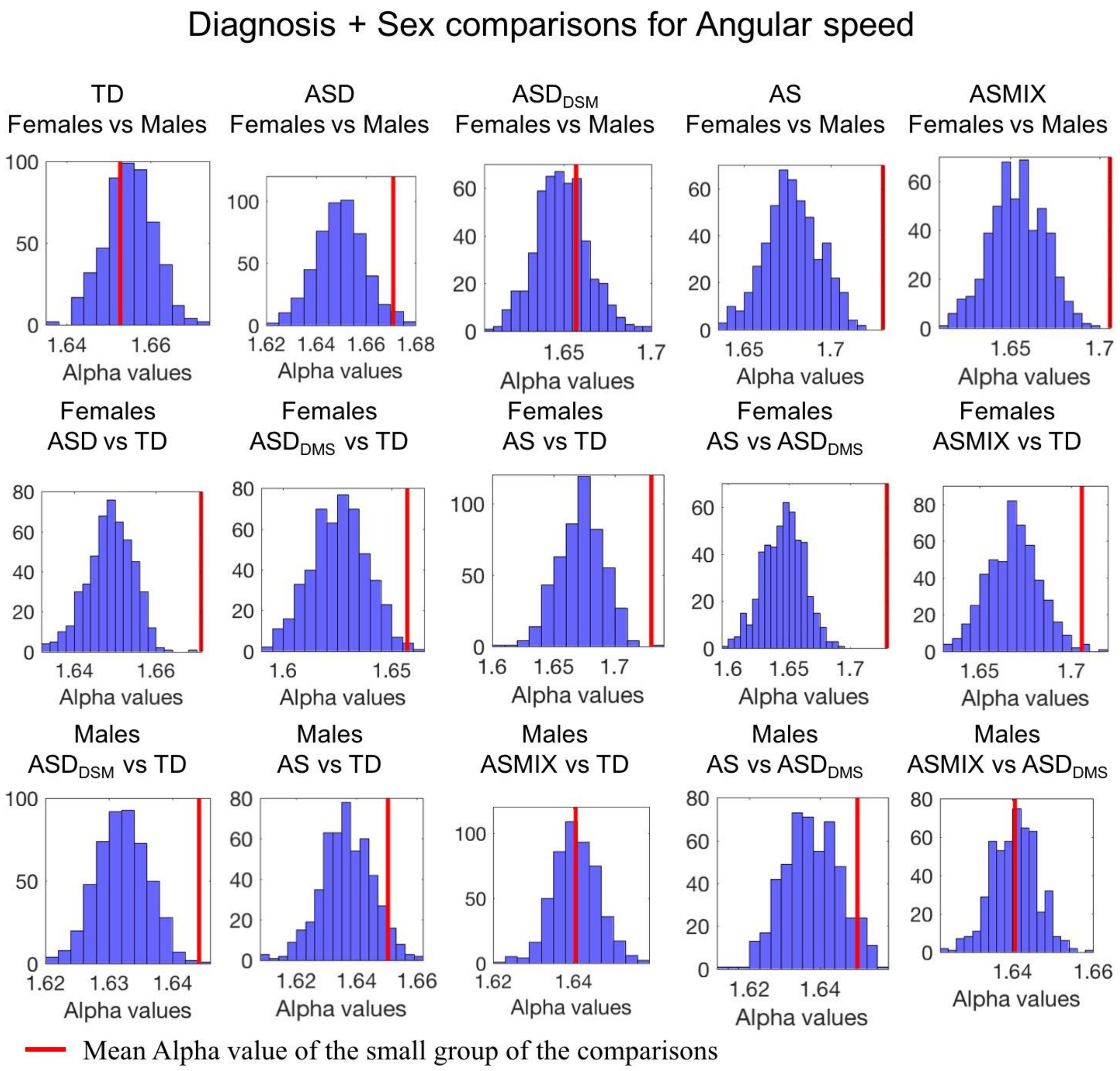
Diagnosis + Sex comparisons for Angular speed
TD Females vs Males
ASD Females vs Males
ASDDSM Females vs Males
AS Females vs Males
ASMIX Females vs Males
Females ASD vs TD
Females ASDDMS vs TD
Females AS vs TD
Females AS vs ASDDMS
Females ASMIX vs TD
Males ASDDSM vs TD
Males AS vs TD
Males ASMIX vs TD
Males AS vs ASDDMS
Males ASMIX vs ASDDMS
Alpha values
Mean Alpha value of the small group of the comparisons

Figure S10.

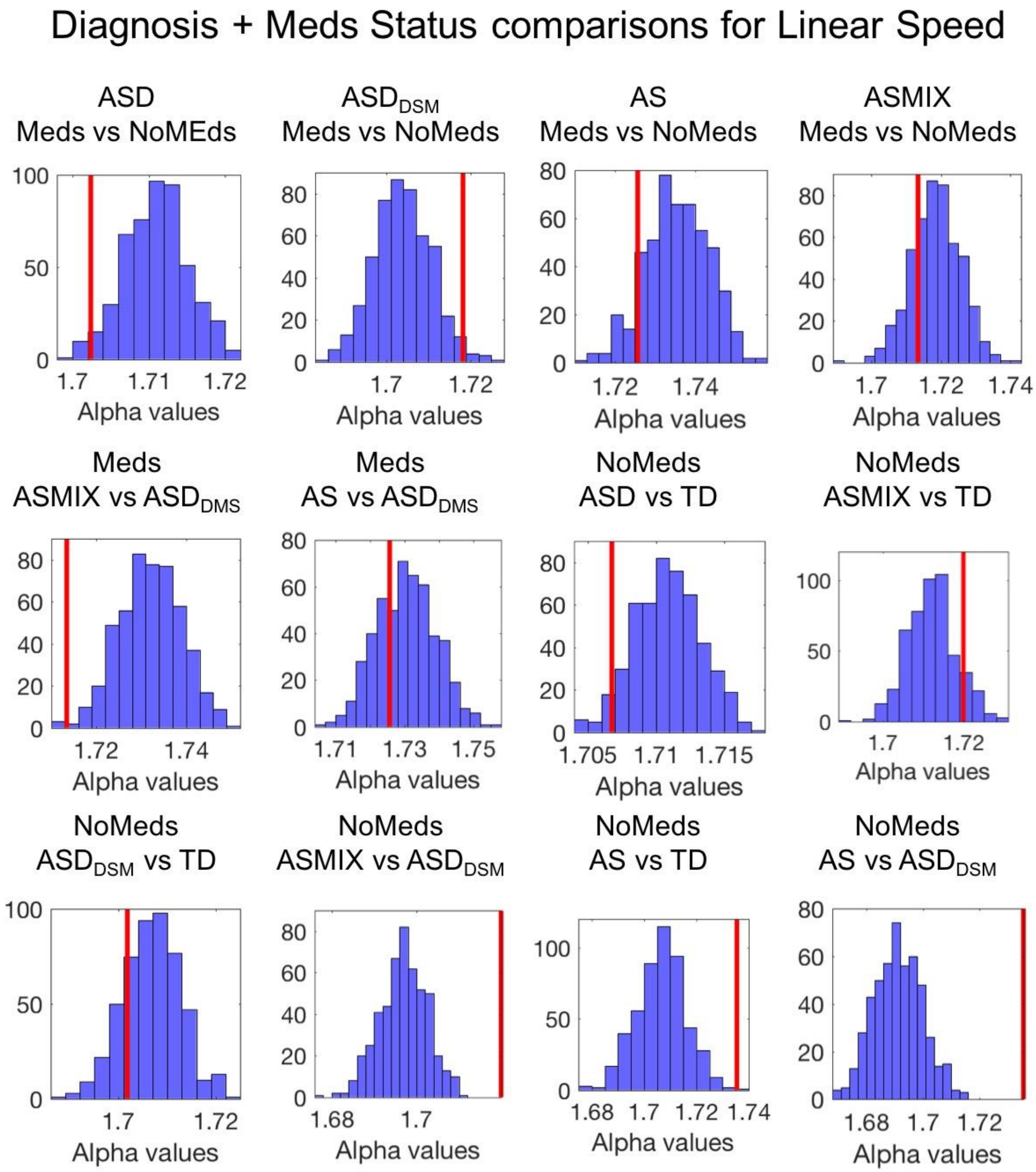

Mean Alpha value of the small group of the comparison

Figure S11.

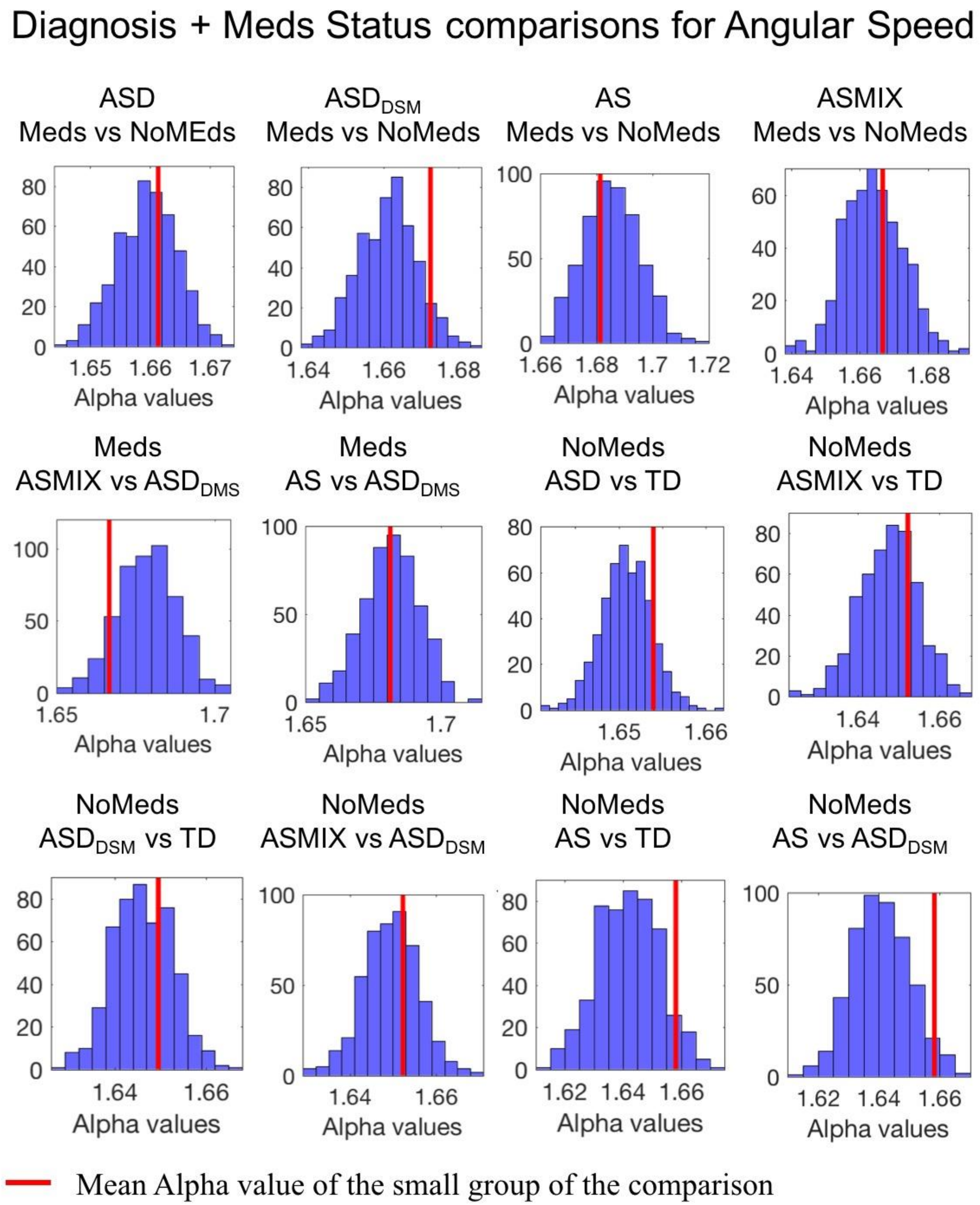
Diagnosis + Meds Status comparisons for Angular Speed
ASD
Meds vs NoMEds
ASDDSM
Meds vs NoMeds
AS
Meds vs NoMeds
ASMIX
Meds vs NoMeds
Meds
ASMIX vs ASDDMS
Meds
AS vs ASDDMS
NoMeds
ASD vs TD
NoMeds
ASMIX vs TD
NoMeds
ASDDSM vs TD
NoMeds
ASMIX vs ASDDSM
NoMeds
AS vs TD
NoMeds
AS vs ASDDSM
Alpha values
Mean Alpha value of the small group of the comparison

Figure S12.

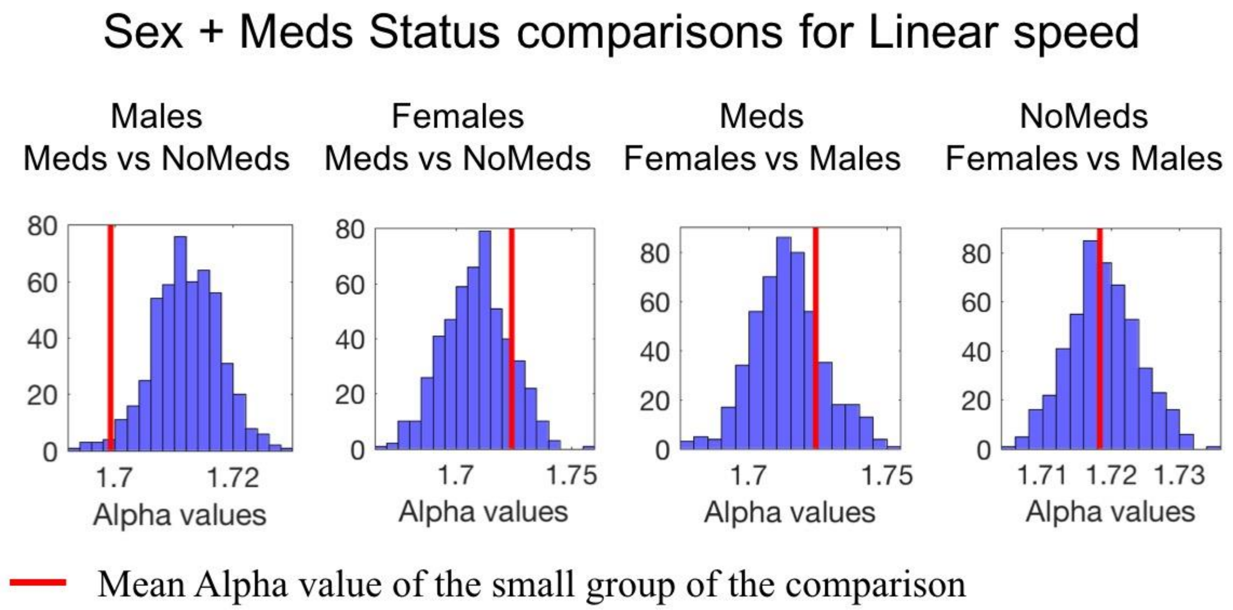

Figure s13.

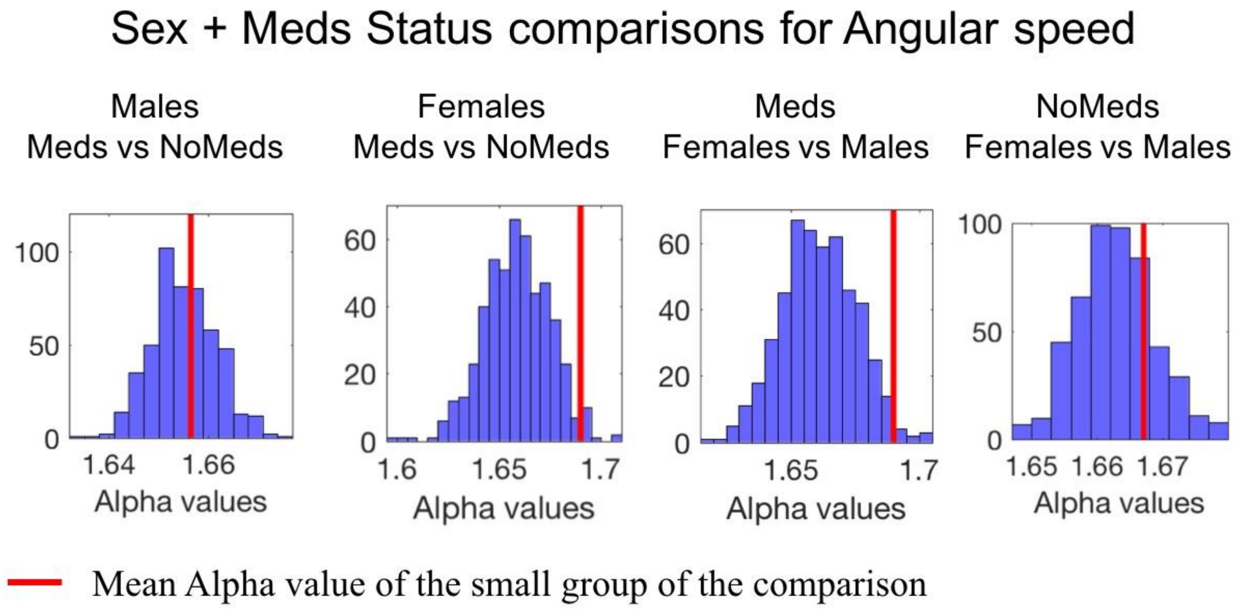

Figure S14.

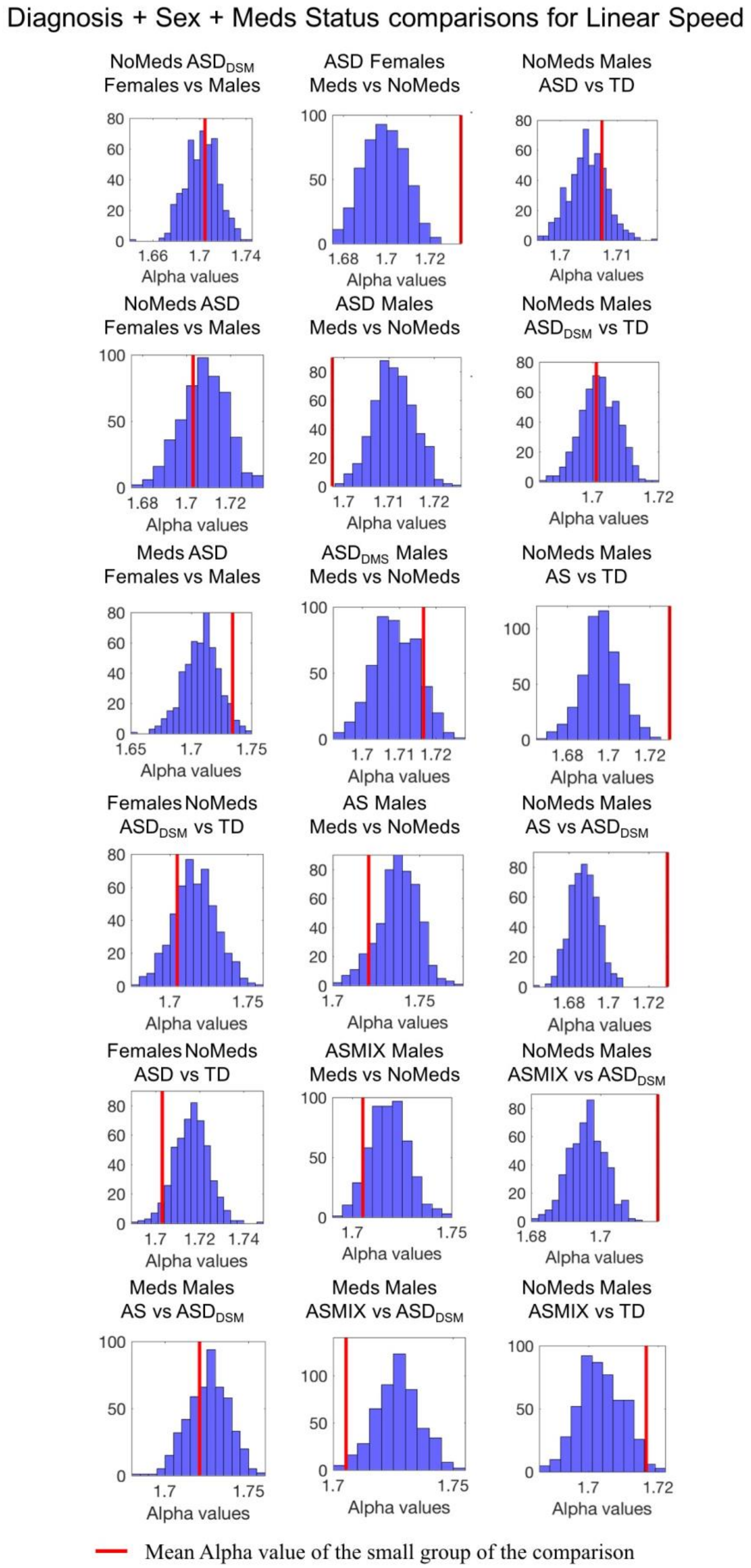
Diagnosis + Sex + Meds Status comparisons for Linear Speed
NoMeds ASD$_{DSM}$ Females vs Males
ASD Females Meds vs NoMeds
NoMeds Males ASD vs TD
NoMeds ASD Females vs Males
ASD Males Meds vs NoMeds
NoMeds Males ASD$_{DSM}$ vs TD
Meds ASD Females vs Males
ASD$_{DMS}$ Males Meds vs NoMeds
NoMeds Males AS vs TD
Females NoMeds ASD$_{DSM}$ vs TD
AS Males Meds vs NoMeds
NoMeds Males AS vs ASD$_{DSM}$
Females NoMeds ASD vs TD
ASMIX Males Meds vs NoMeds
NoMeds Males ASMIX vs ASD$_{DSM}$
Meds Males AS vs ASD$_{DSM}$
Meds Males ASMIX vs ASD$_{DSM}$
NoMeds Males ASMIX vs TD
Alpha values
Mean Alpha value of the small group of the comparison

Figure S15.

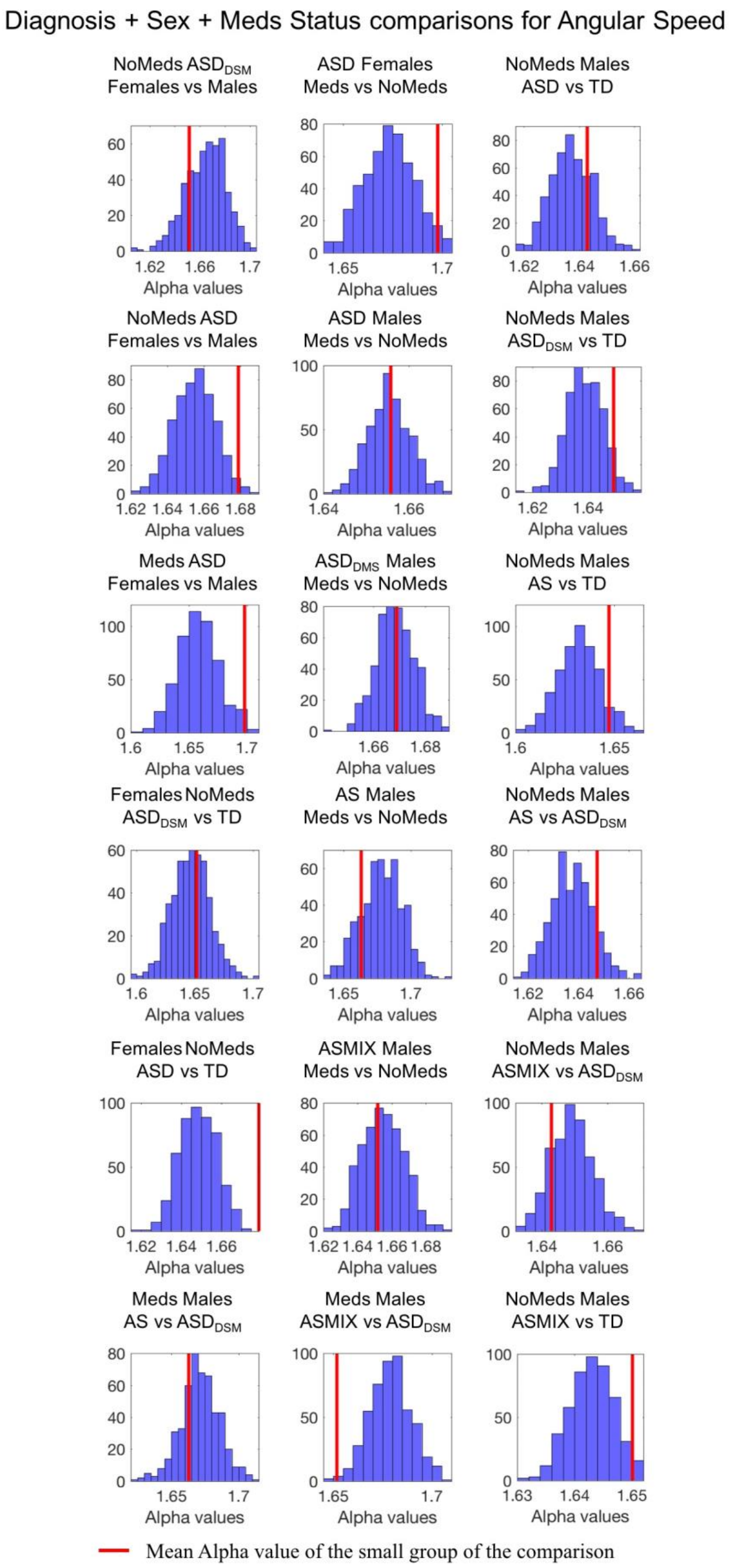
Diagnosis + Sex + Meds Status comparisons for Angular Speed
NoMeds ASD$_{DSM}$ Females vs Males
ASD Females Meds vs NoMeds
NoMeds Males ASD vs TD
NoMeds ASD Females vs Males
ASD Males Meds vs NoMeds
NoMeds Males ASD$_{DSM}$ vs TD
Meds ASD Females vs Males
ASD$_{DMS}$ Males Meds vs NoMeds
NoMeds Males AS vs TD
Females NoMeds ASD$_{DSM}$ vs TD
AS Males Meds vs NoMeds
NoMeds Males AS vs ASD$_{DSM}$
Females NoMeds ASD vs TD
ASMIX Males Meds vs NoMeds
NoMeds Males ASMIX vs ASD$_{DSM}$
Meds Males AS vs ASD$_{DSM}$
Meds Males ASMIX vs ASD$_{DSM}$
NoMeds Males ASMIX vs TD
Alpha values
Mean Alpha value of the small group of the comparison

Figure S16.

Figure S17-S24 show de distributions of p-values (log2 scale for better visualization) with the red line as the reference .05 significance-level value for the comparisons displayed in the previous figures (Figures 9-16). In each plot the mean and mode of the p-value distribution is provided (p1 and p1 respectively).

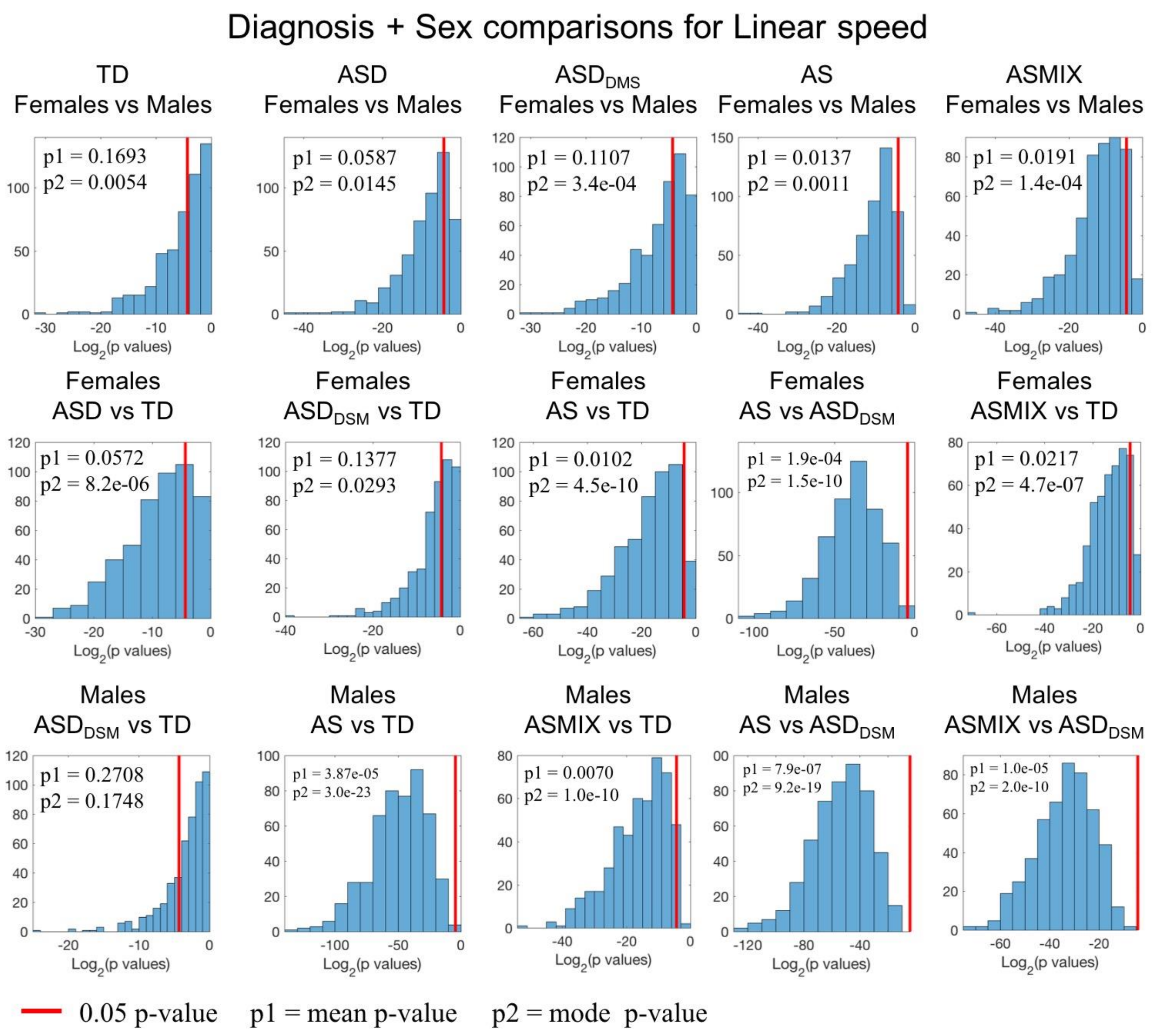

Figure S17. Related to the Figure 9.

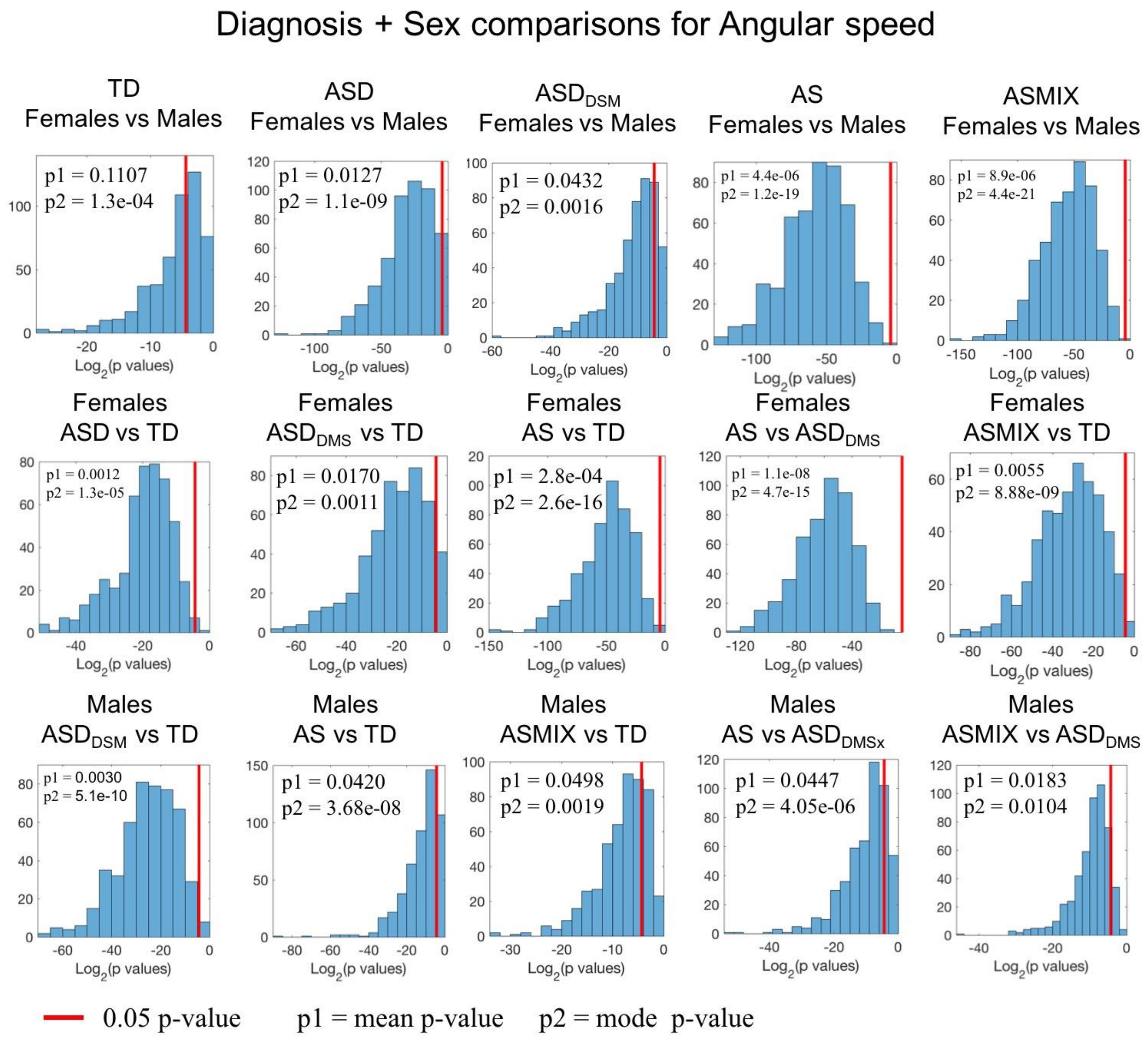

Figure S18. Related to the Figure 10.

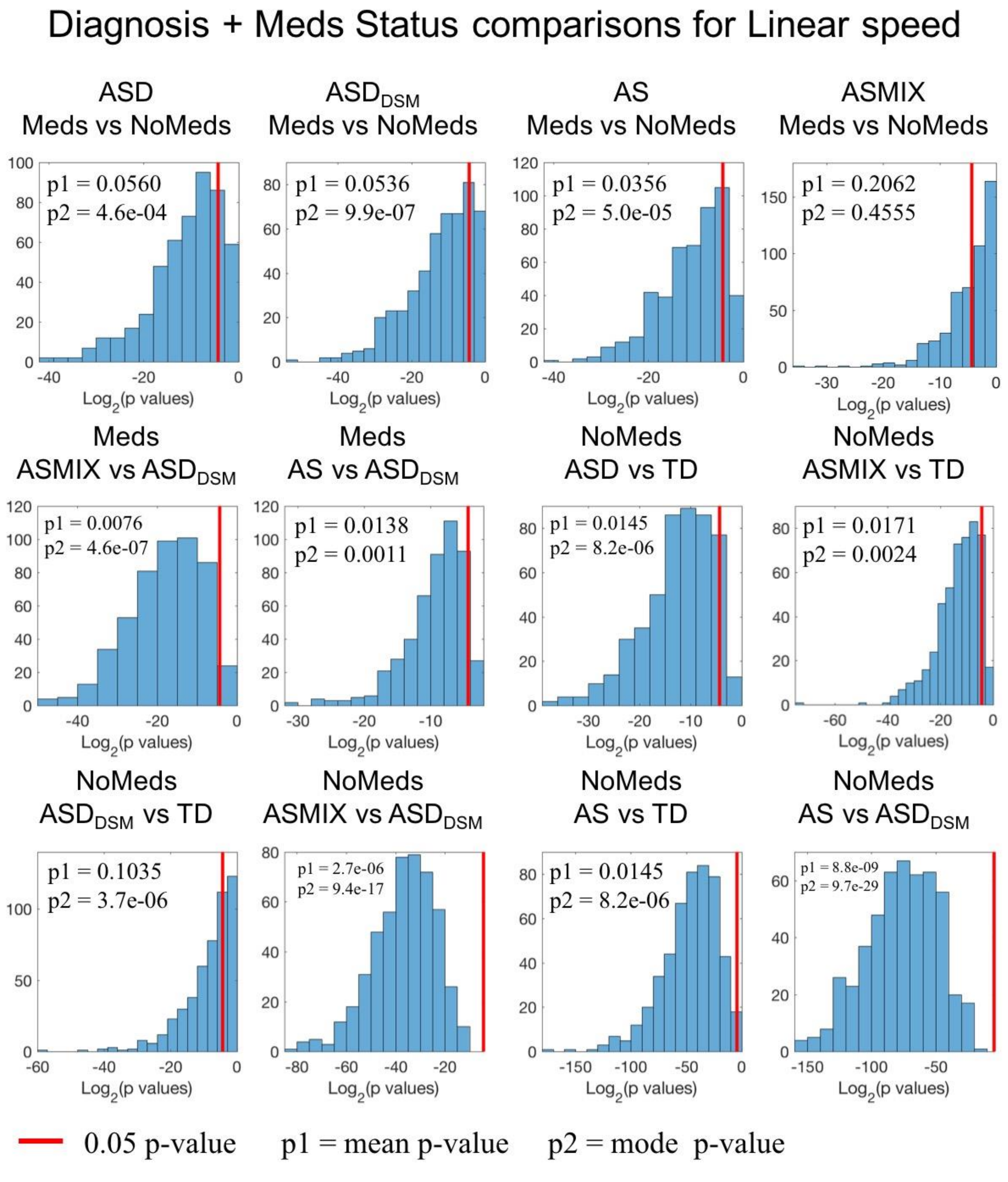

Figure S19. Related to the Figure 11.

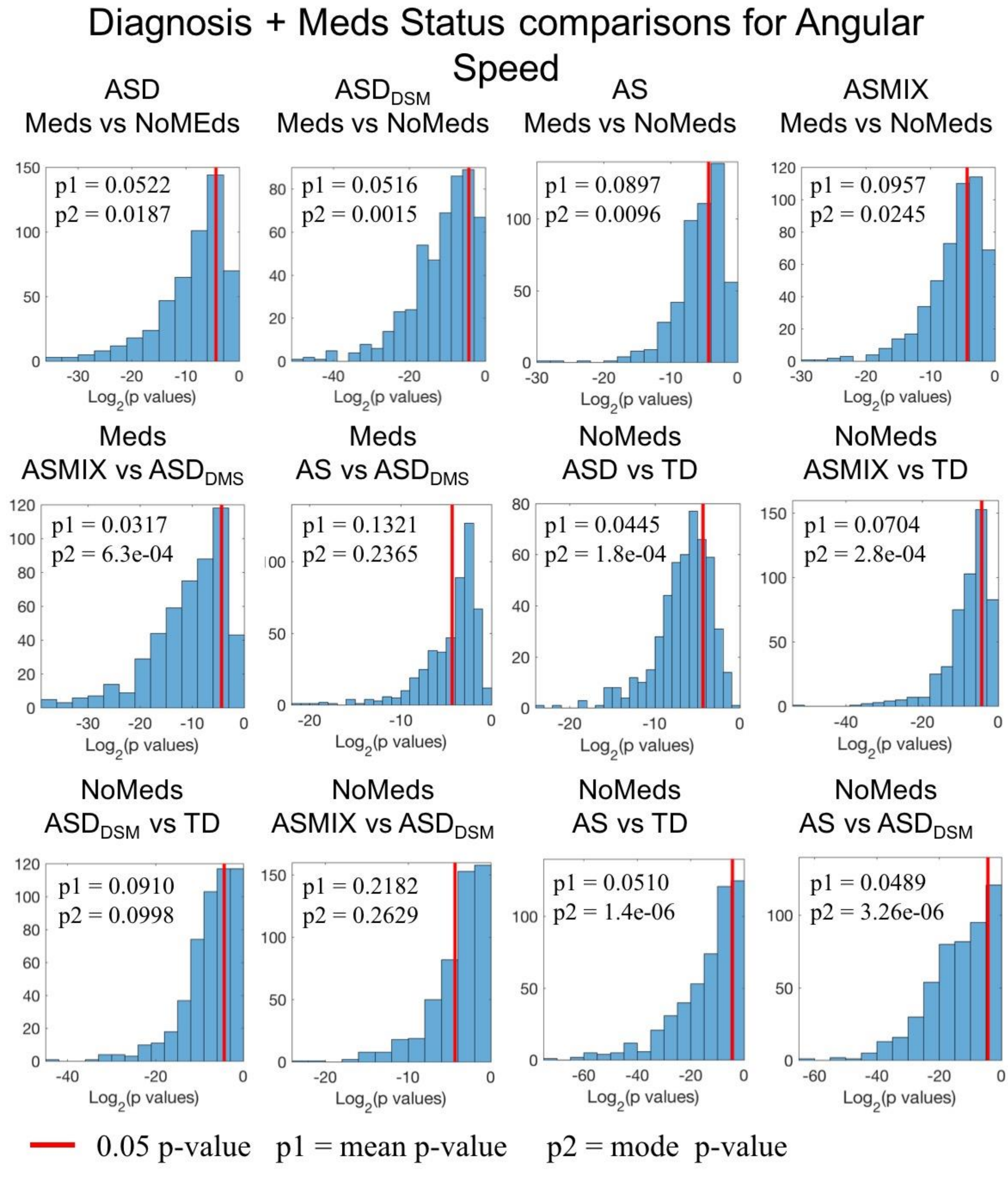

Figure S20. Related to the Figure 12.

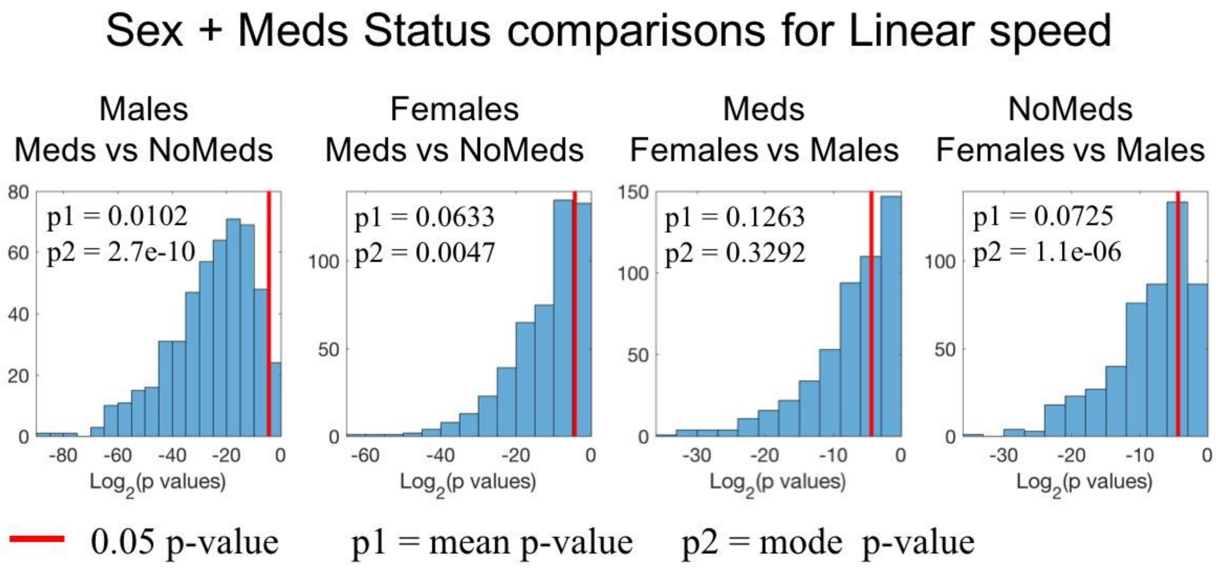

Figure S21. Related to the Figure 13.

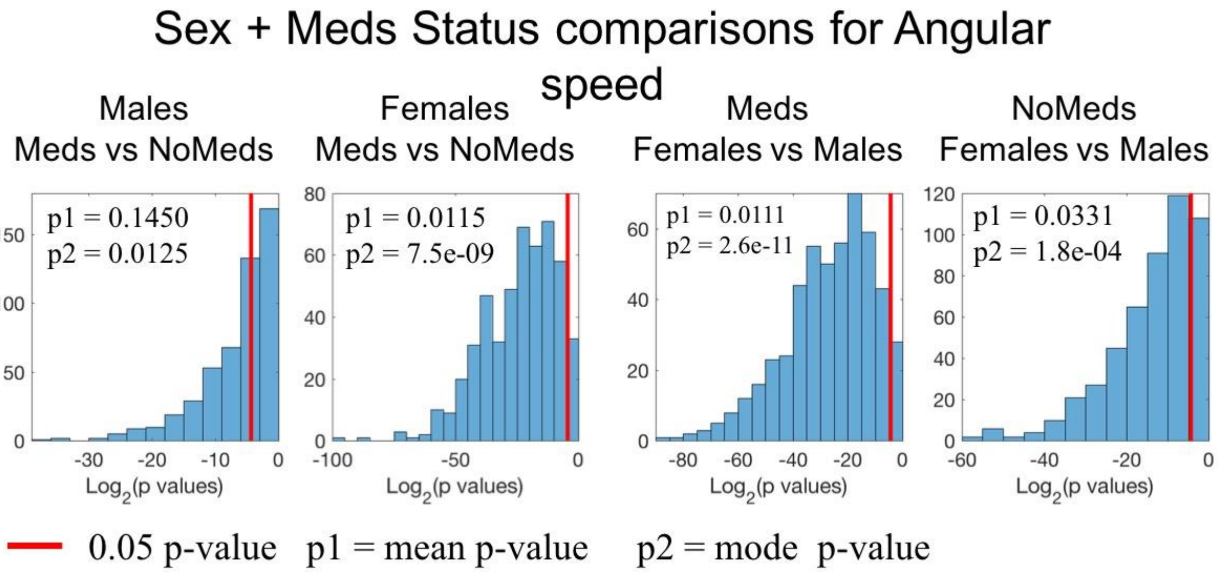

Figure S22. Related to the Figure 14.

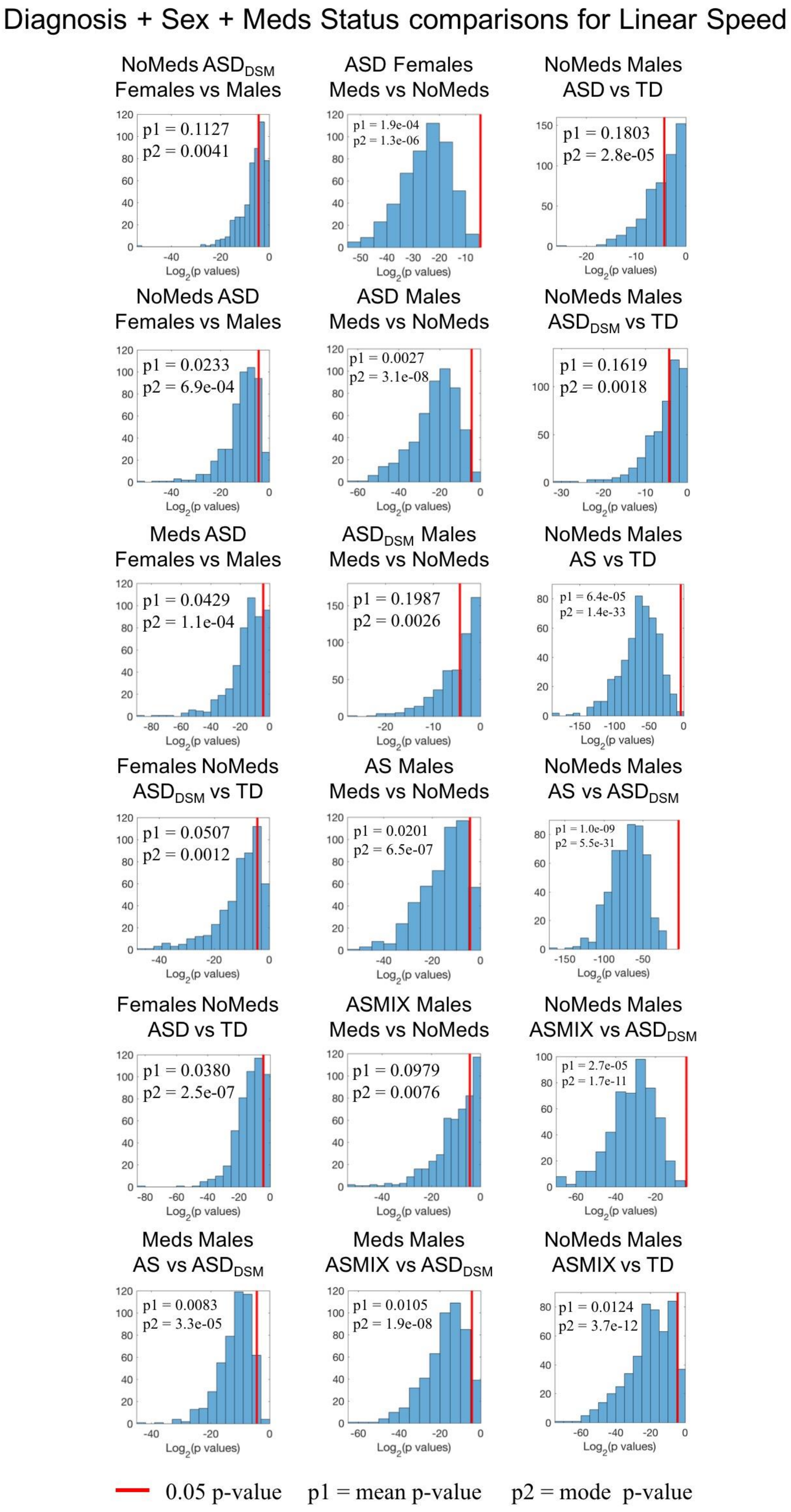

Figure S23. Related to the Figure 15.

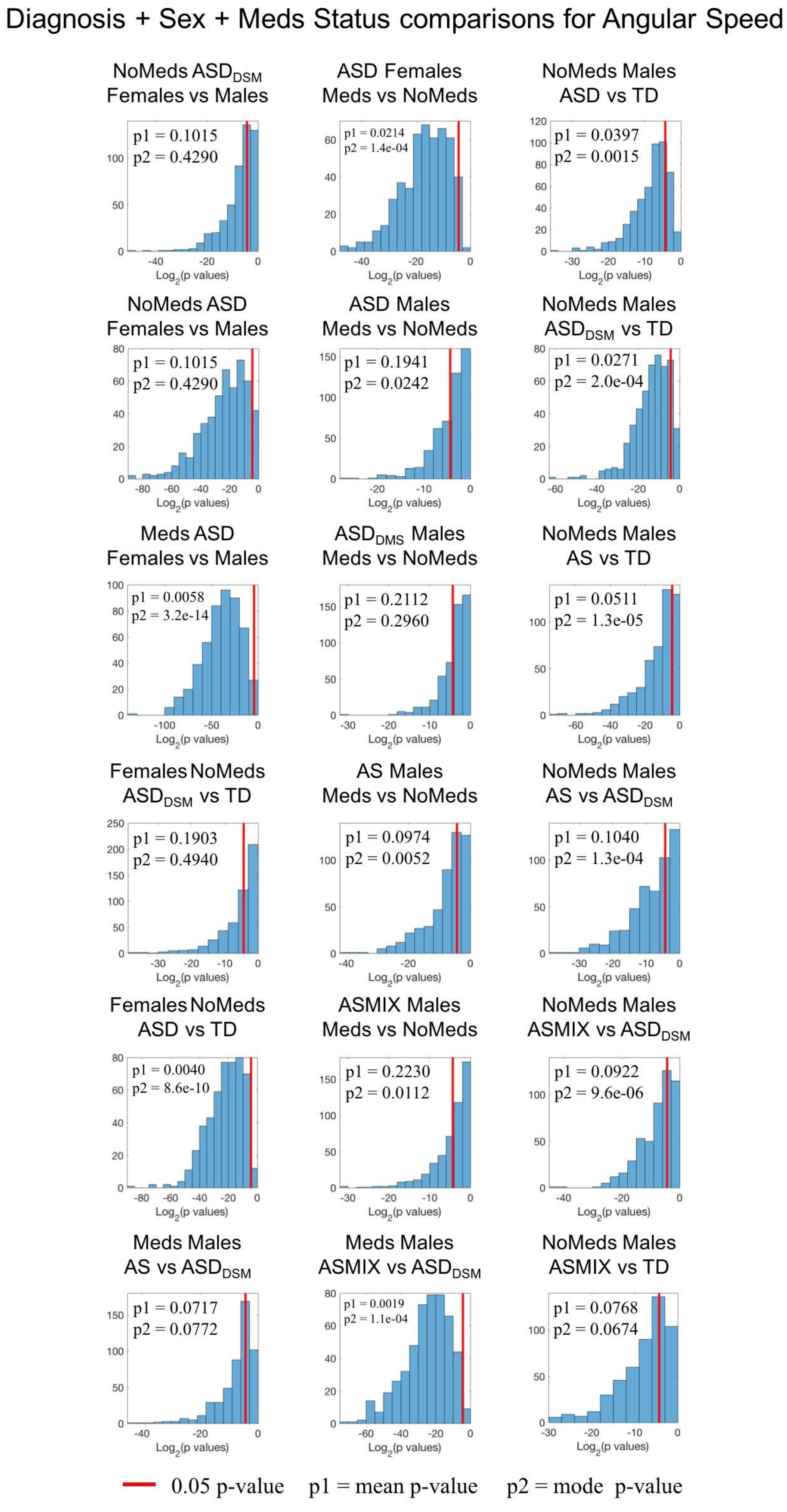

Figure S24. Related to the Figure 16.

Figure S25-S32 report the comparisons of the estimated shape and scale Gamma values with confidence intervals on the Gamma parameter plane extracted from the α values distributions for the different groups.

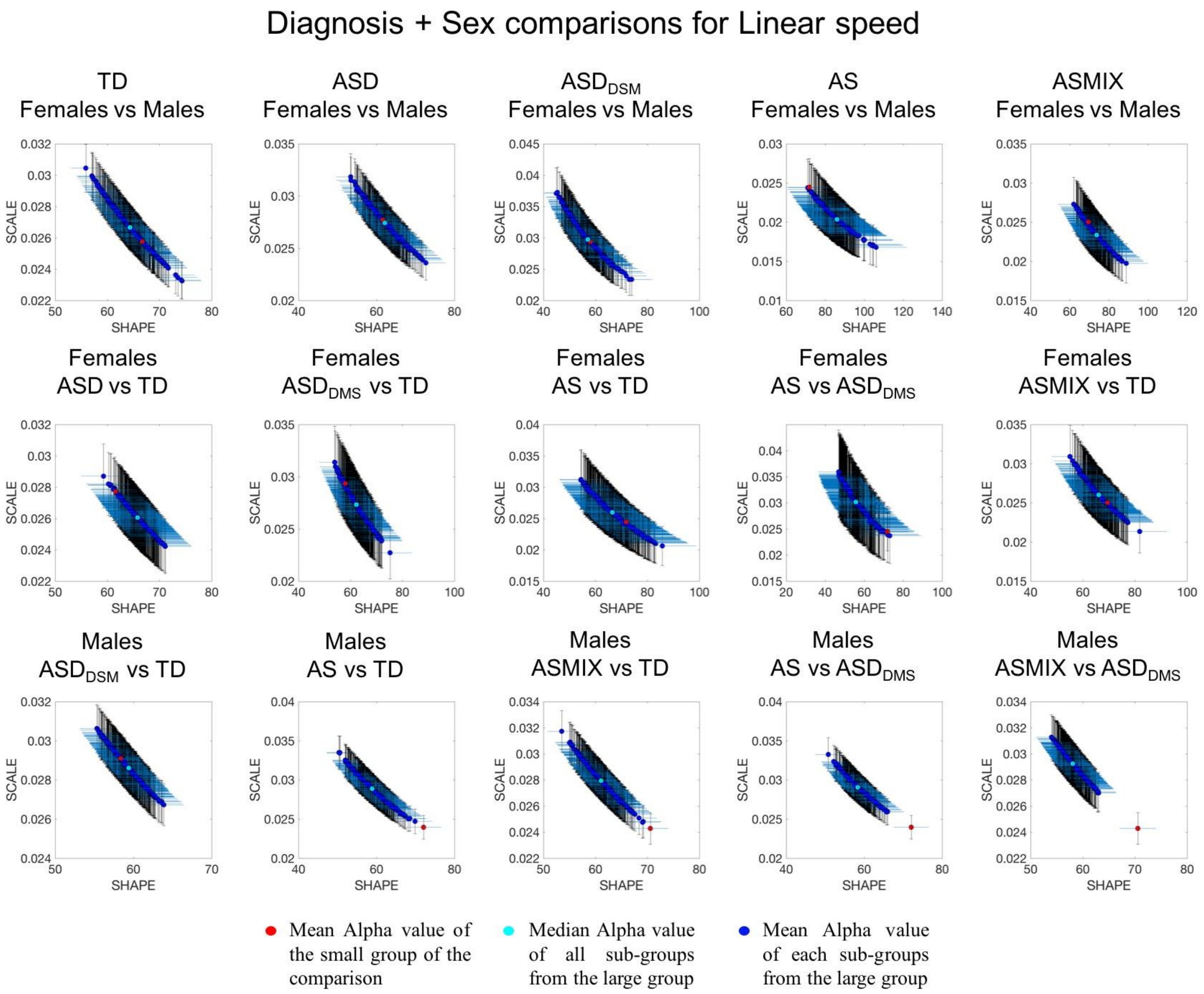

Figure S25.

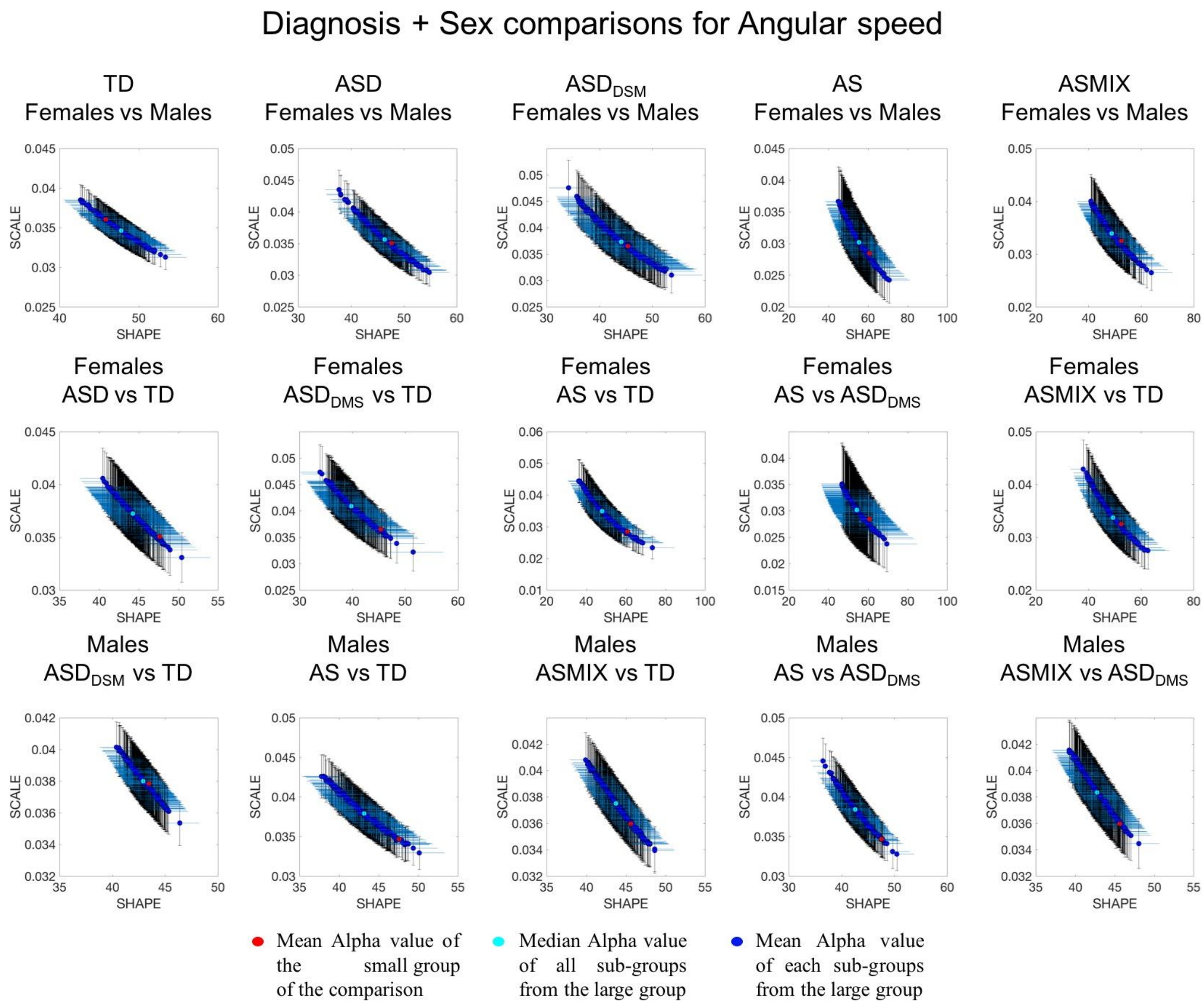
Diagnosis + Sex comparisons for Angular speed
TD Females vs Males
ASD Females vs Males
ASDDSM Females vs Males
AS Females vs Males
ASMIX Females vs Males
Females ASD vs TD
Females ASDDMS vs TD
Females AS vs TD
Females AS vs ASDDMS
Females ASMIX vs TD
Males ASDDSM vs TD
Males AS vs TD
Males ASMIX vs TD
Males AS vs ASDDMS
Males ASMIX vs ASDDMS
SCALE
SHAPE
Mean Alpha value of the small group of the comparison
Median Alpha value of all sub-groups from the large group
Mean Alpha value of each sub-groups from the large group

Figure S26.

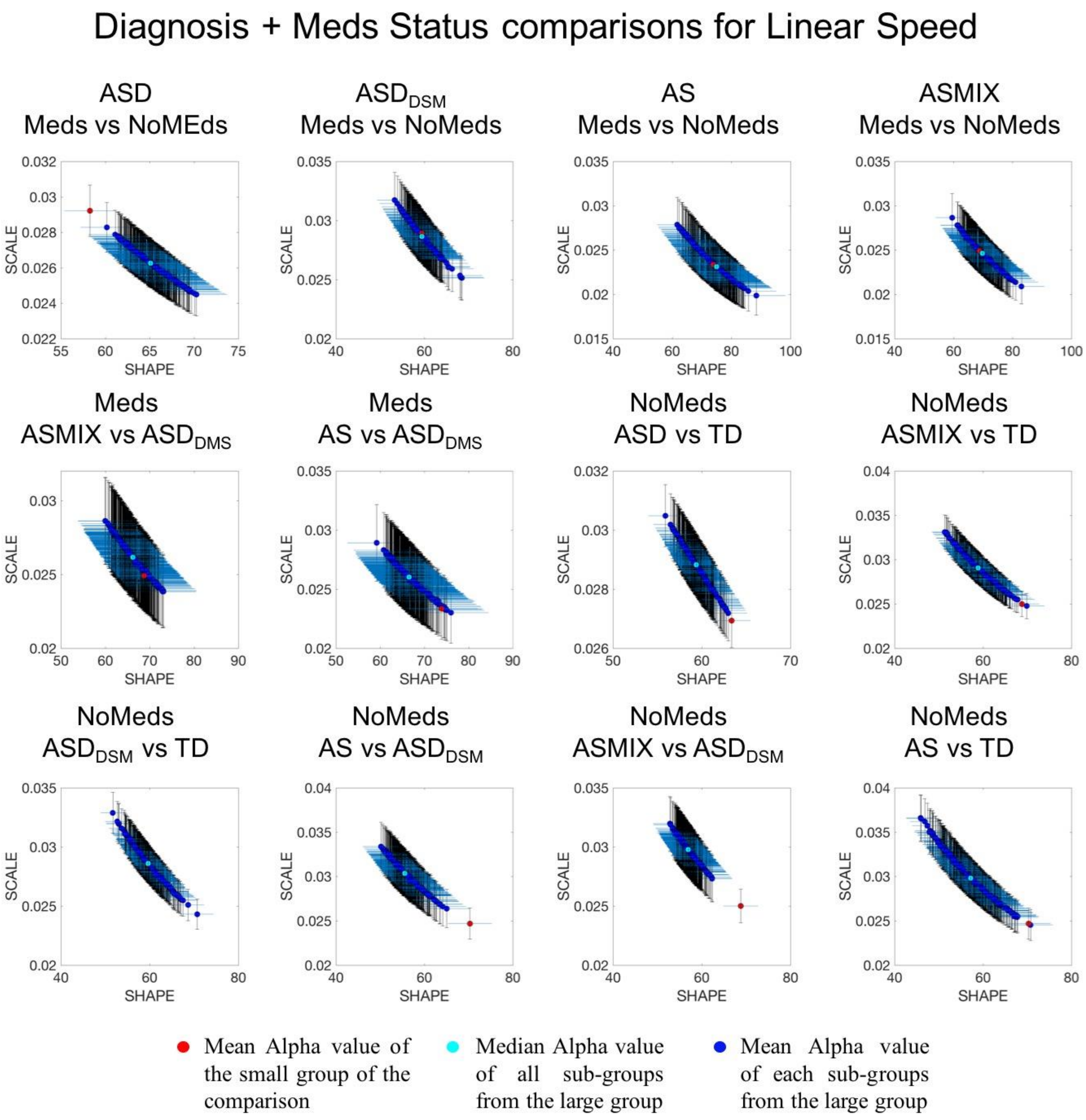
Diagnosis + Meds Status comparisons for Linear Speed
ASD Meds vs NoMEds
ASD$_{DSM}$ Meds vs NoMeds
AS Meds vs NoMeds
ASMIX Meds vs NoMeds
Meds ASMIX vs ASD$_{DMS}$
Meds AS vs ASD$_{DMS}$
NoMeds ASD vs TD
NoMeds ASMIX vs TD
NoMeds ASD$_{DSM}$ vs TD
NoMeds AS vs ASD$_{DSM}$
NoMeds ASMIX vs ASD$_{DSM}$
NoMeds AS vs TD
SCALE
SHAPE
Mean Alpha value of the small group of the comparison
Median Alpha value of all sub-groups from the large group
Mean Alpha value of each sub-groups from the large group

Figure S27.

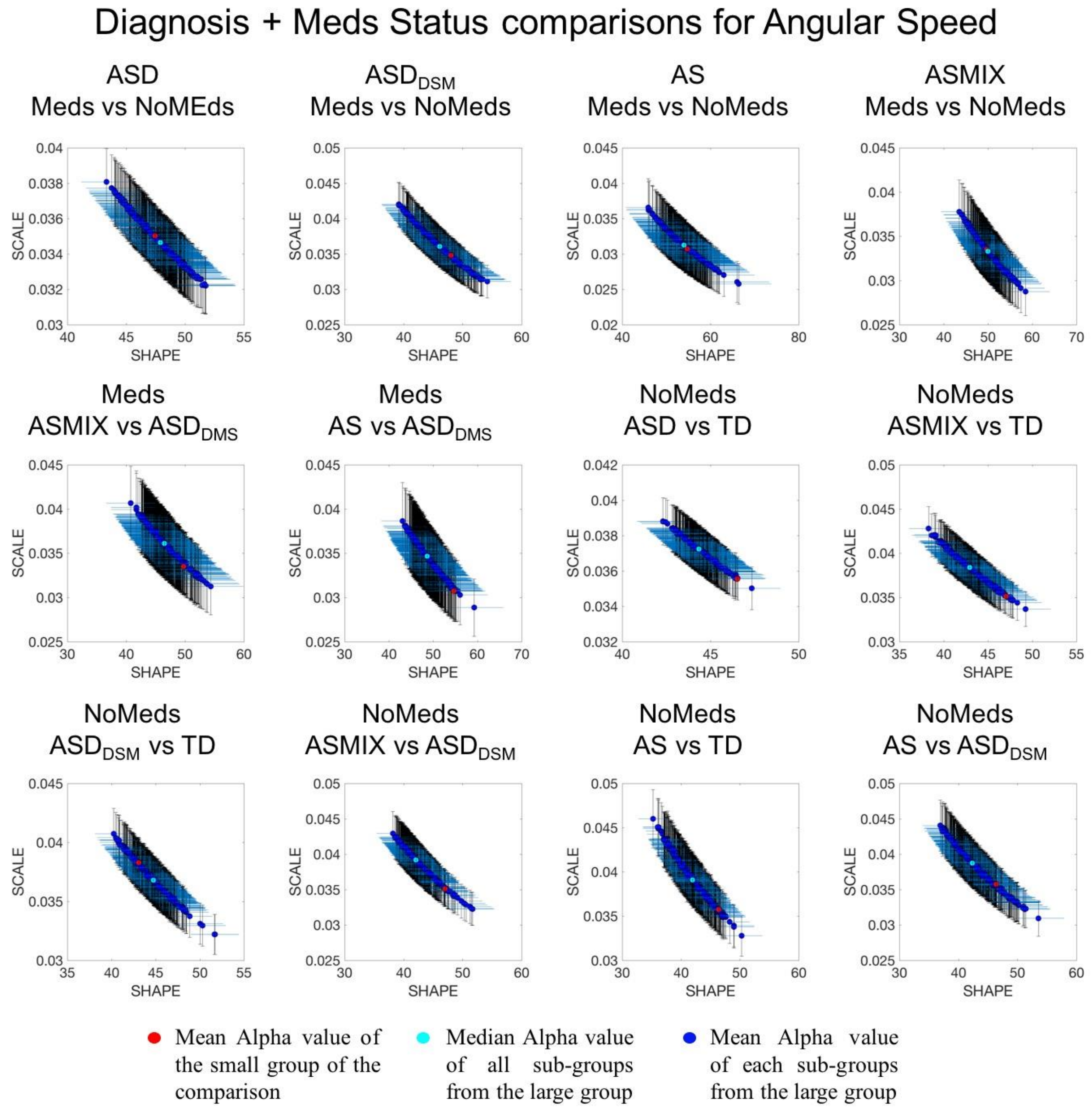
Diagnosis + Meds Status comparisons for Angular Speed
ASD Meds vs NoMEds
ASD_{DSM} Meds vs NoMeds
AS Meds vs NoMeds
ASMIX Meds vs NoMeds
Meds ASMIX vs ASD_{DMS}
Meds AS vs ASD_{DMS}
NoMeds ASD vs TD
NoMeds ASMIX vs TD
NoMeds ASD_{DSM} vs TD
NoMeds ASMIX vs ASD_{DSM}
NoMeds AS vs TD
NoMeds AS vs ASD_{DSM}
SHAPE
SCALE
Mean Alpha value of the small group of the comparison
Median Alpha value of all sub-groups from the large group
Mean Alpha value of each sub-groups from the large group

Figure S28.

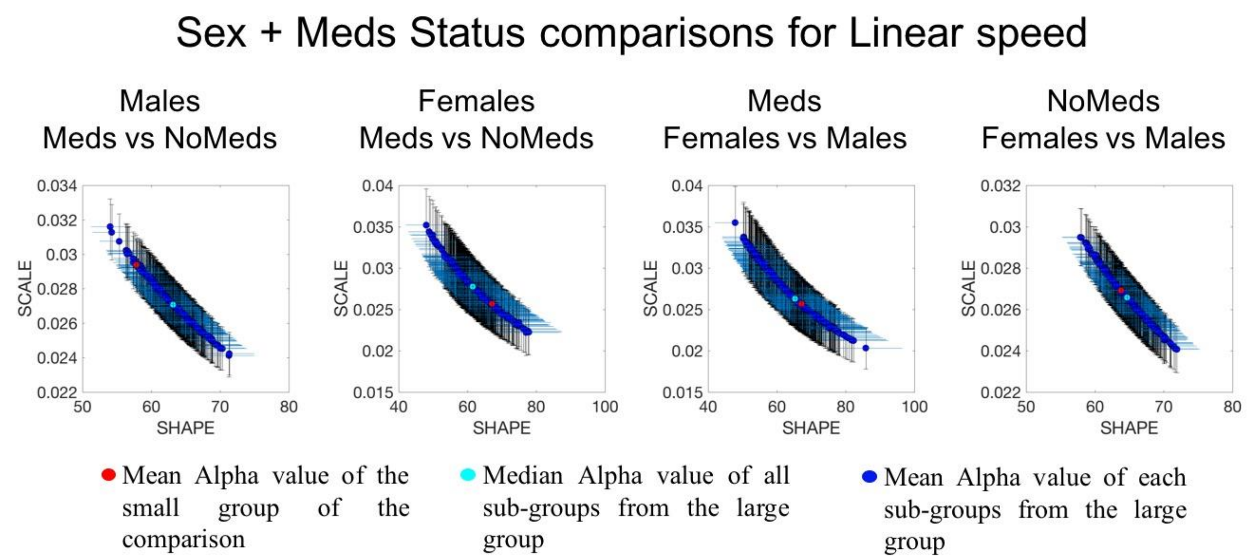

Figure S29.

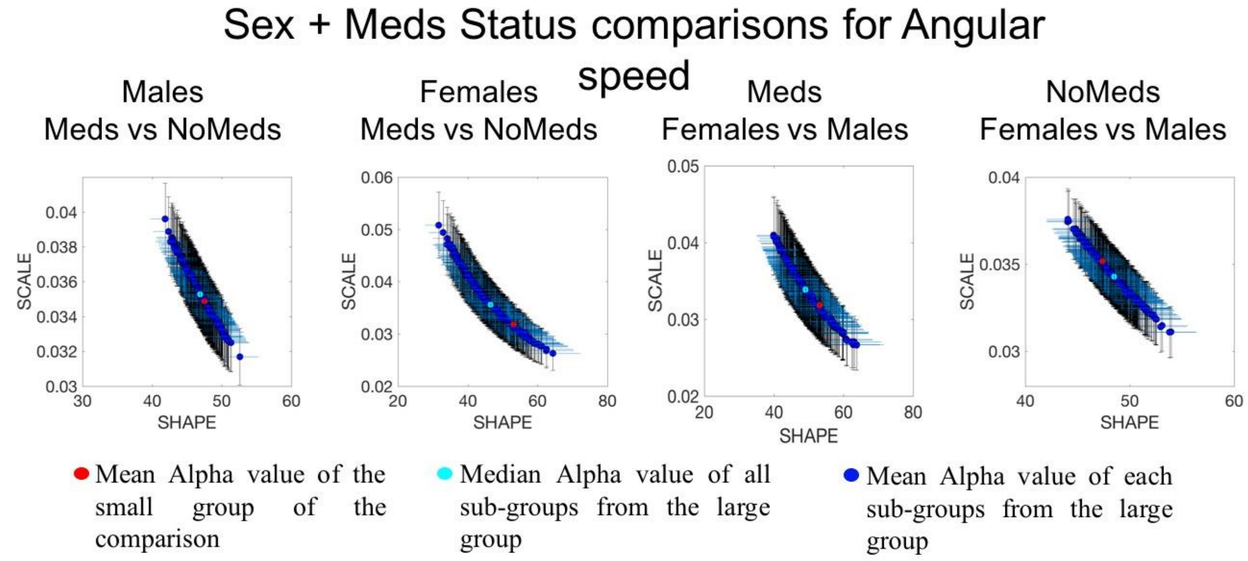

Figure S30.

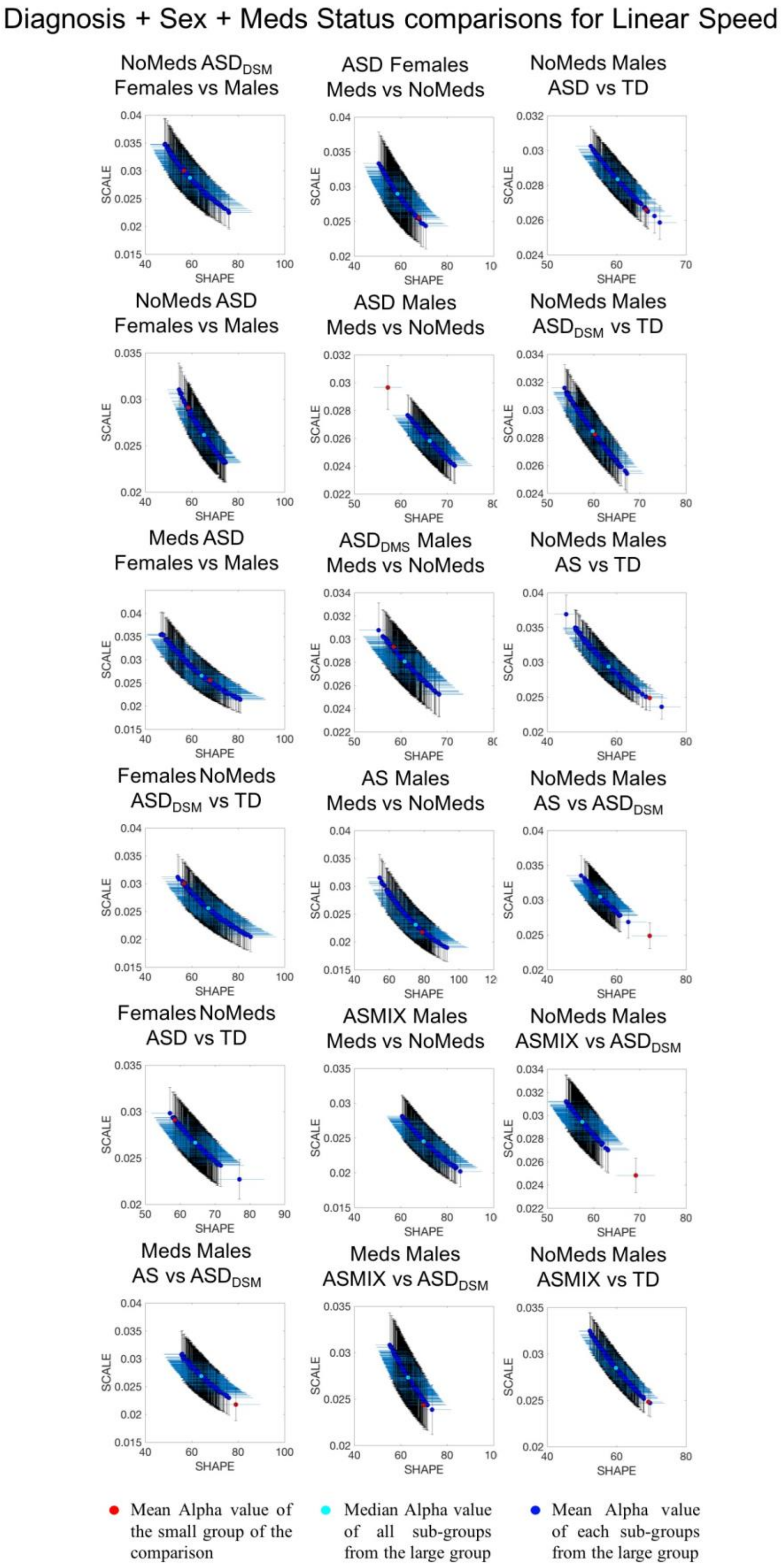
Diagnosis + Sex + Meds Status comparisons for Linear Speed
NoMeds ASD_{DSM} Females vs Males
ASD Females Meds vs NoMeds
NoMeds Males ASD vs TD
NoMeds ASD Females vs Males
ASD Males Meds vs NoMeds
NoMeds Males ASD_{DSM} vs TD
Meds ASD Females vs Males
ASD_{DMS} Males Meds vs NoMeds
NoMeds Males AS vs TD
Females NoMeds ASD_{DSM} vs TD
AS Males Meds vs NoMeds
NoMeds Males AS vs ASD_{DSM}
Females NoMeds ASD vs TD
ASMIX Males Meds vs NoMeds
NoMeds Males ASMIX vs ASD_{DSM}
Meds Males AS vs ASD_{DSM}
Meds Males ASMIX vs ASD_{DSM}
NoMeds Males ASMIX vs TD
SCALE
SHAPE
Mean Alpha value of the small group of the comparison
Median Alpha value of all sub-groups from the large group
Mean Alpha value of each sub-groups from the large group

Figure S31.

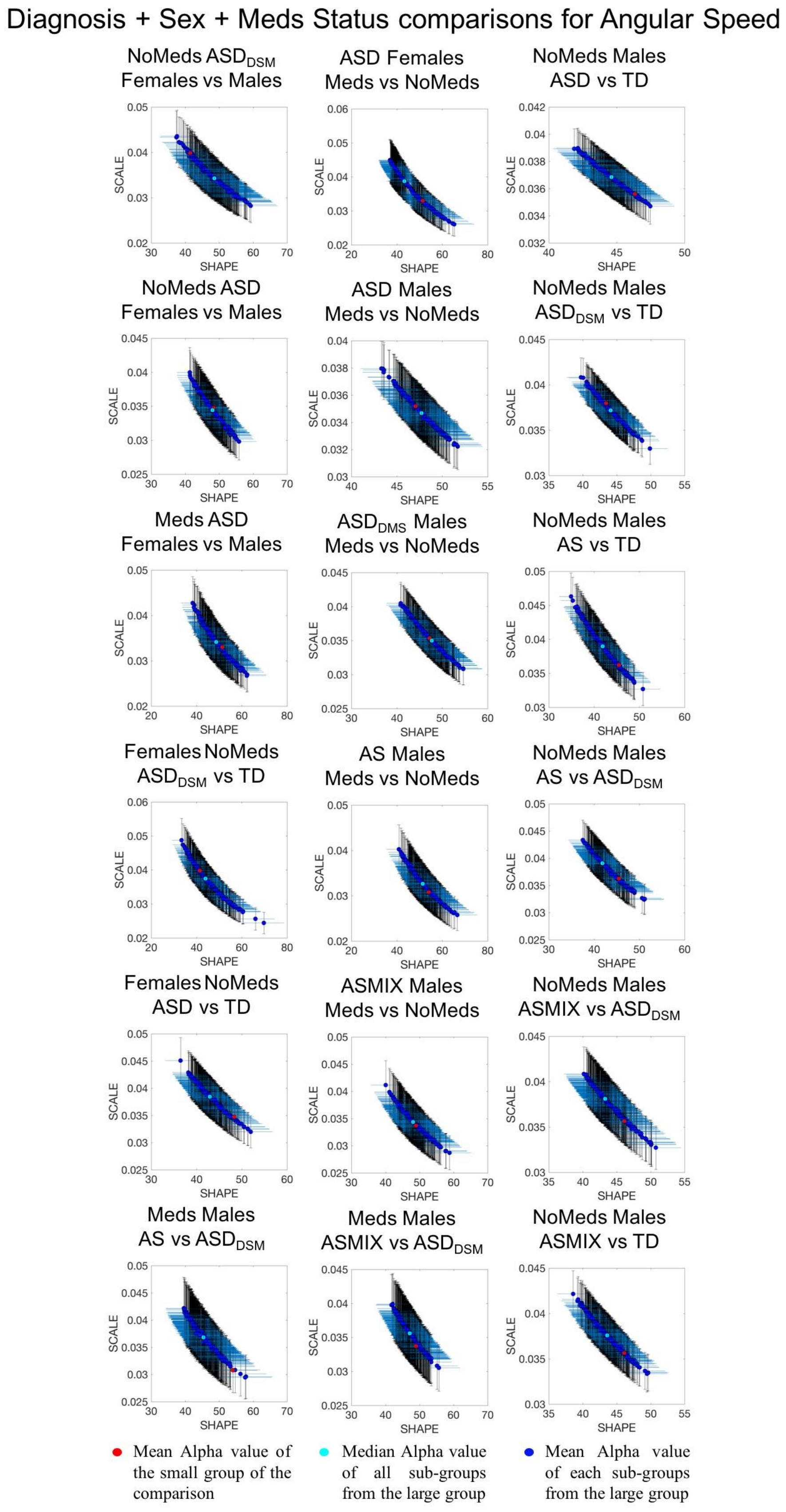
Diagnosis + Sex + Meds Status comparisons for Angular Speed
NoMeds ASD$_{DSM}$ Females vs Males
ASD Females Meds vs NoMeds
NoMeds Males ASD vs TD
NoMeds ASD Females vs Males
ASD Males Meds vs NoMeds
NoMeds Males ASD$_{DSM}$ vs TD
Meds ASD Females vs Males
ASD$_{DMS}$ Males Meds vs NoMeds
NoMeds Males AS vs TD
Females NoMeds ASD$_{DSM}$ vs TD
AS Males Meds vs NoMeds
NoMeds Males AS vs ASD$_{DSM}$
Females NoMeds ASD vs TD
ASMIX Males Meds vs NoMeds
NoMeds Males ASMIX vs ASD$_{DSM}$
Meds Males AS vs ASD$_{DSM}$
Meds Males ASMIX vs ASD$_{DSM}$
NoMeds Males ASMIX vs TD
SCALE
SHAPE
Mean Alpha value of the small group of the comparison
Median Alpha value of all sub-groups from the large group
Mean Alpha value of each sub-groups from the large group

Figure S32.

Figures S33-S40 show the comparisons of the PDFs empirically estimated over the α values distribution for the different groups.

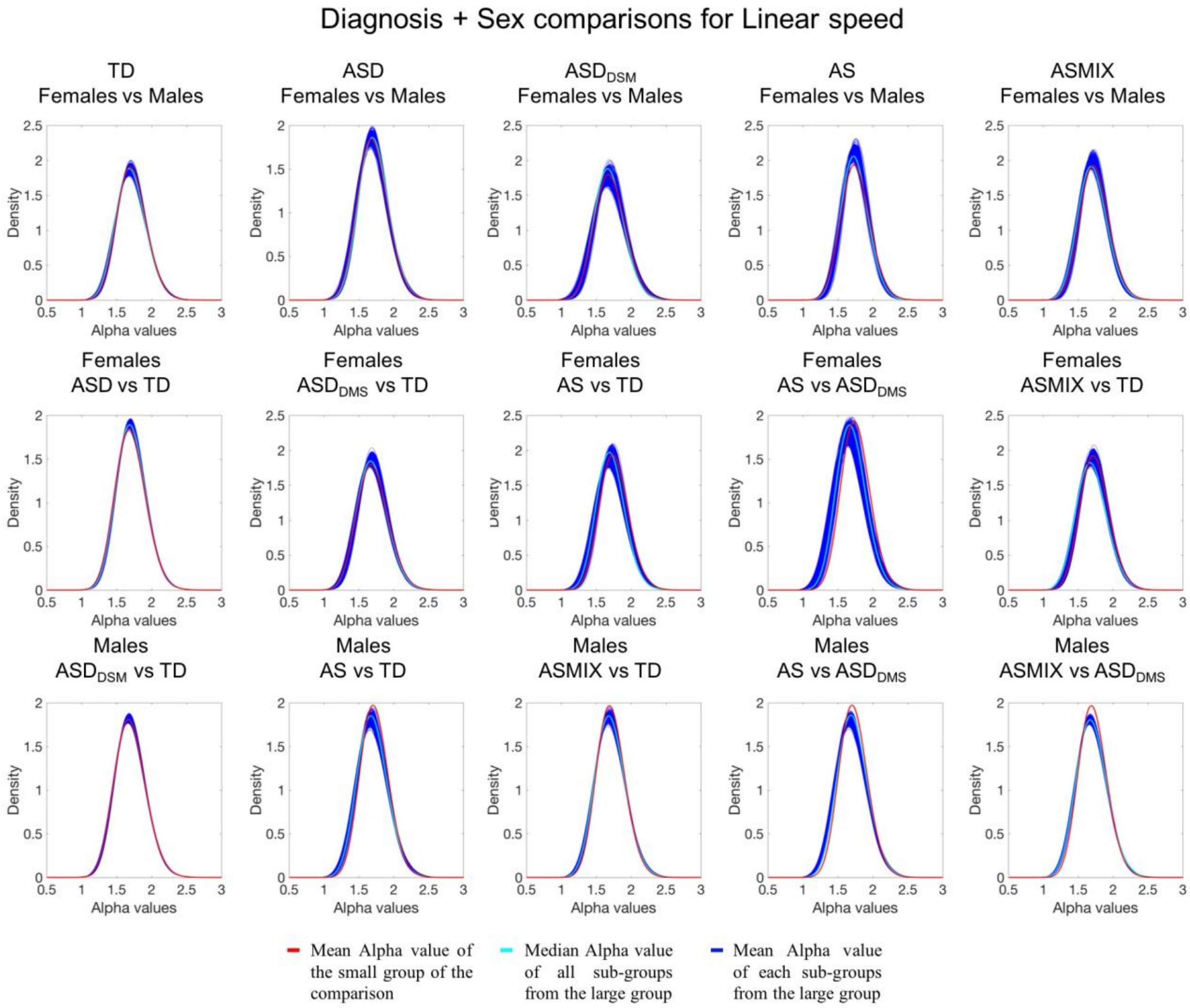

Figure S33.

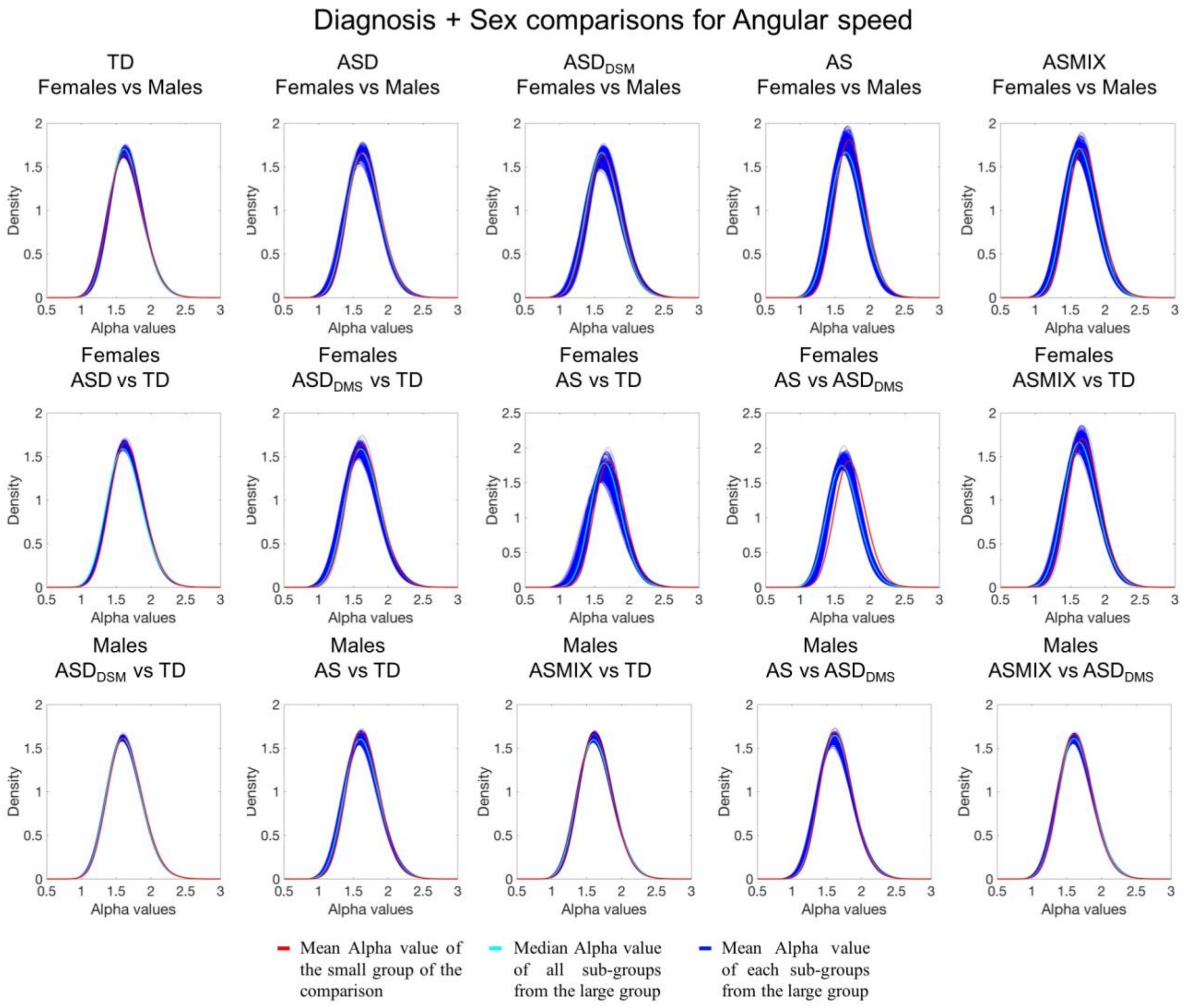

Diagnosis + Sex comparisons for Angular speed
TD Females vs Males
ASD Females vs Males
ASDDSM Females vs Males
AS Females vs Males
ASMIX Females vs Males
Females ASD vs TD
Females ASDDMS vs TD
Females AS vs TD
Females AS vs ASDDMS
Females ASMIX vs TD
Males ASDDSM vs TD
Males AS vs TD
Males ASMIX vs TD
Males AS vs ASDDMS
Males ASMIX vs ASDDMS
Density
Alpha values
Mean Alpha value of the small group of the comparison
Median Alpha value of all sub-groups from the large group
Mean Alpha value of each sub-groups from the large group

Figure S34.

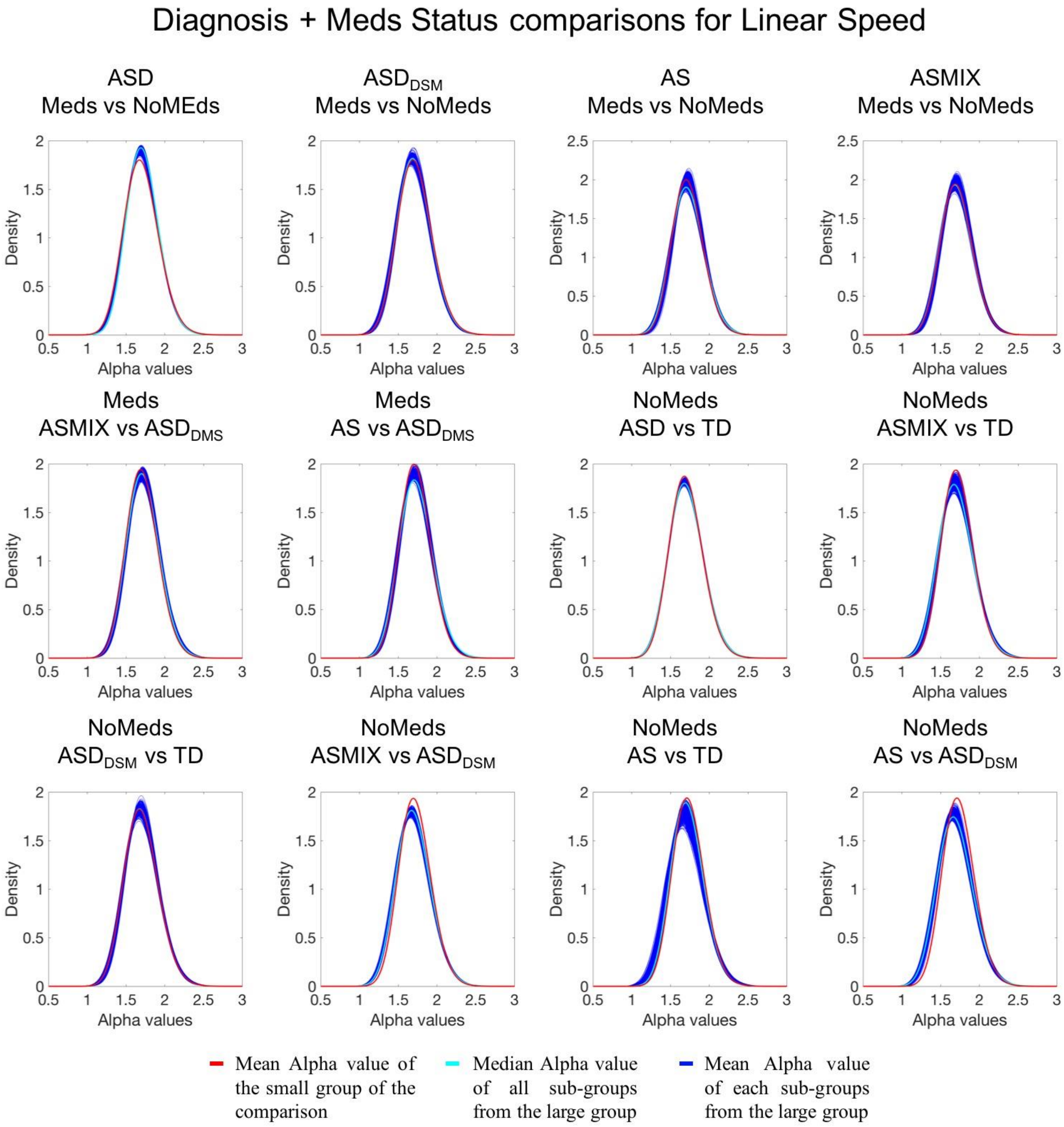
Diagnosis + Meds Status comparisons for Linear Speed
ASD
Meds vs NoMEds
ASDDSM
Meds vs NoMeds
AS
Meds vs NoMeds
ASMIX
Meds vs NoMeds
Meds
ASMIX vs ASDDMS
Meds
AS vs ASDDMS
NoMeds
ASD vs TD
NoMeds
ASMIX vs TD
NoMeds
ASDDSM vs TD
NoMeds
ASMIX vs ASDDSM
NoMeds
AS vs TD
NoMeds
AS vs ASDDSM
Density
Alpha values
Mean Alpha value of the small group of the comparison
Median Alpha value of all sub-groups from the large group
Mean Alpha value of each sub-groups from the large group

Figure S35.

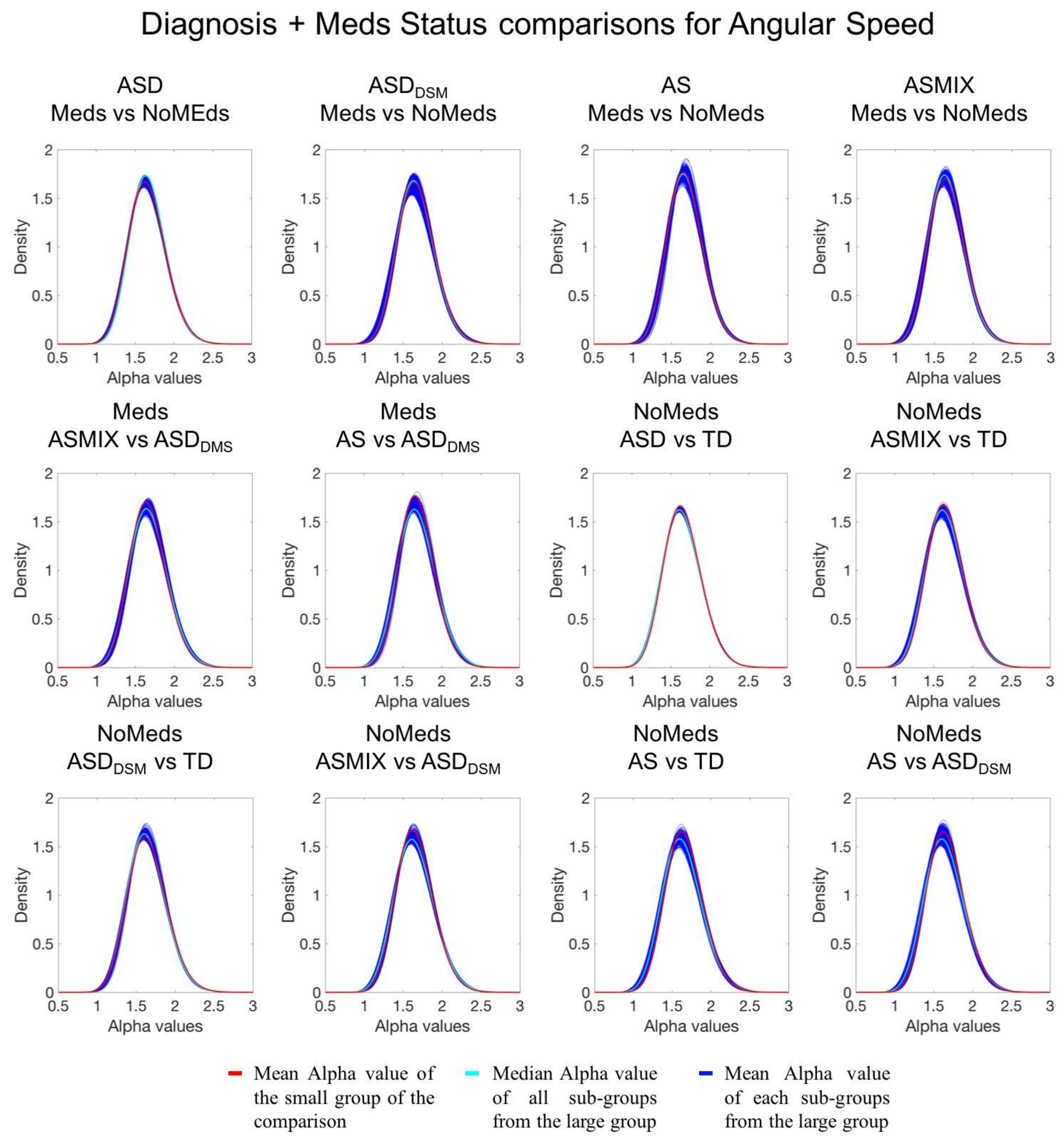
Diagnosis + Meds Status comparisons for Angular Speed
ASD
Meds vs NoMEds
ASD$_{DSM}$
Meds vs NoMeds
AS
Meds vs NoMeds
ASMIX
Meds vs NoMeds
Meds
ASMIX vs ASD$_{DMS}$
Meds
AS vs ASD$_{DMS}$
NoMeds
ASD vs TD
NoMeds
ASMIX vs TD
NoMeds
ASD$_{DSM}$ vs TD
NoMeds
ASMIX vs ASD$_{DSM}$
NoMeds
AS vs TD
NoMeds
AS vs ASD$_{DSM}$
Density
Alpha values
Mean Alpha value of the small group of the comparison
Median Alpha value of all sub-groups from the large group
Mean Alpha value of each sub-groups from the large group

Figure S36.

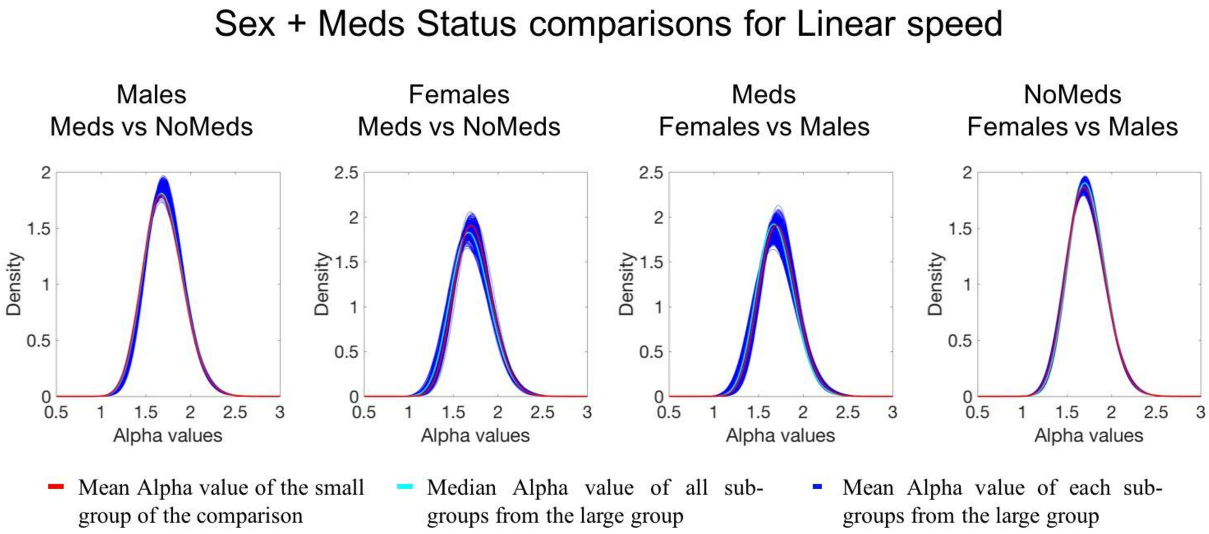

Figure S37.

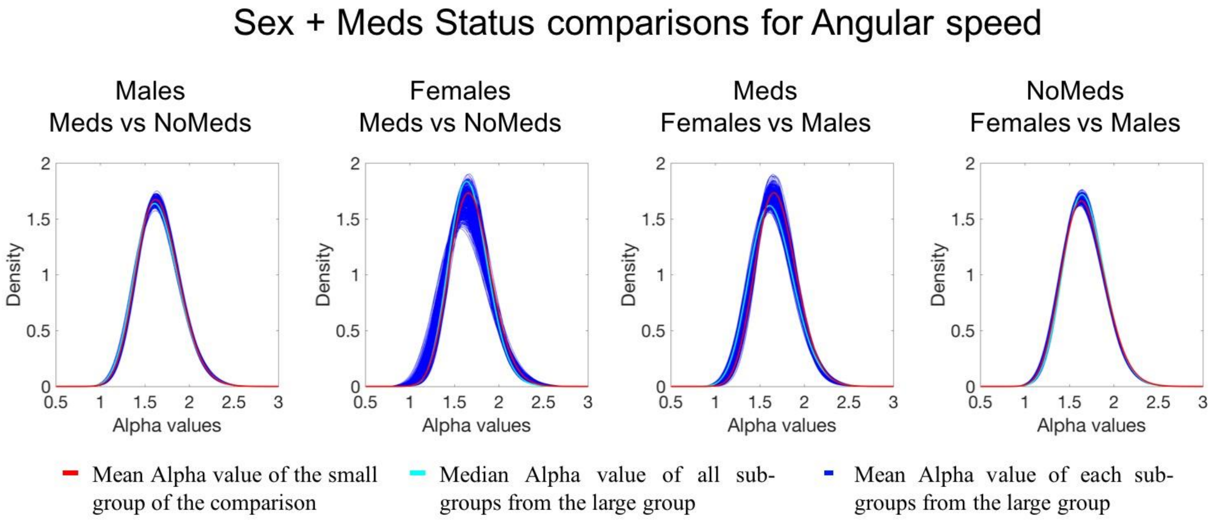

Figure S38.

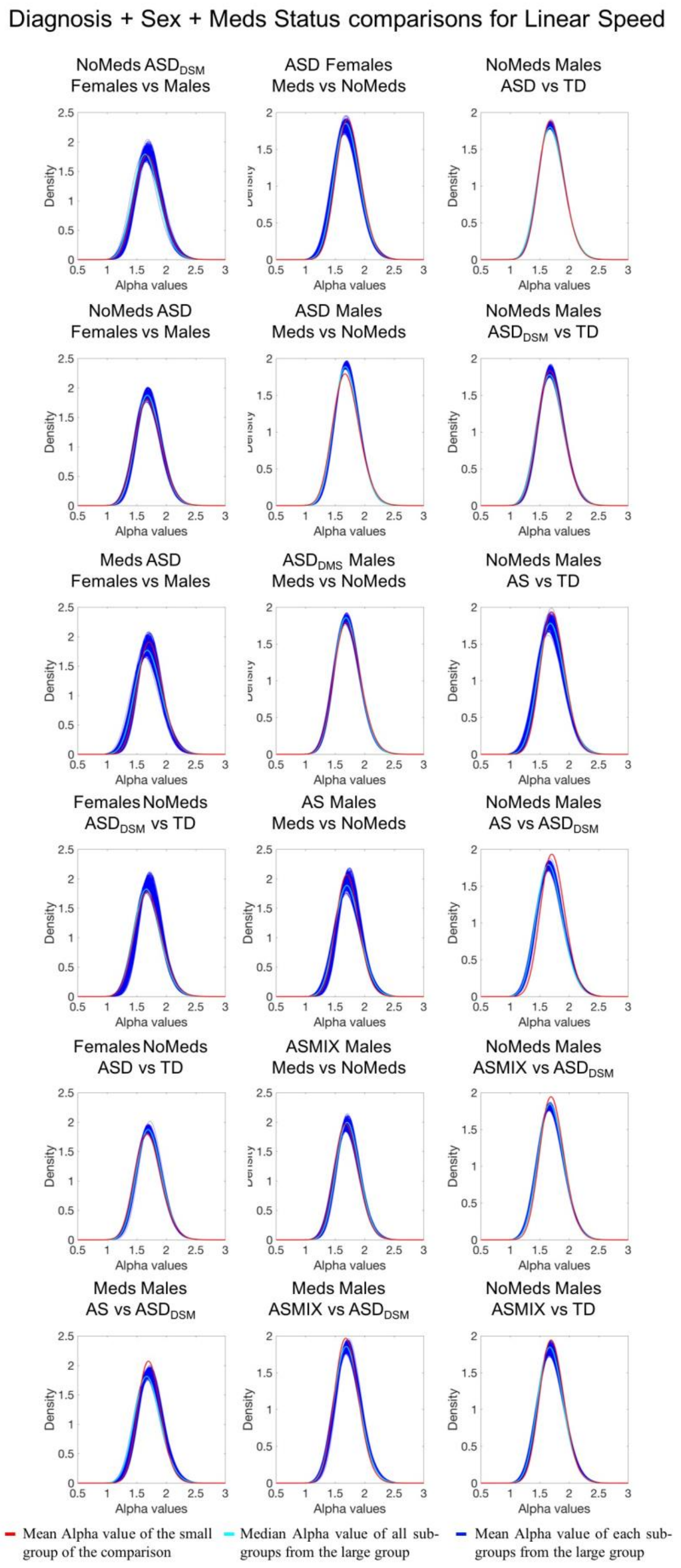
Diagnosis + Sex + Meds Status comparisons for Linear Speed
NoMeds ASD$_{DSM}$ Females vs Males
ASD Females Meds vs NoMeds
NoMeds Males ASD vs TD
NoMeds ASD Females vs Males
ASD Males Meds vs NoMeds
NoMeds Males ASD$_{DSM}$ vs TD
Meds ASD Females vs Males
ASD$_{DMS}$ Males Meds vs NoMeds
NoMeds Males AS vs TD
Females NoMeds ASD$_{DSM}$ vs TD
AS Males Meds vs NoMeds
NoMeds Males AS vs ASD$_{DSM}$
Females NoMeds ASD vs TD
ASMIX Males Meds vs NoMeds
NoMeds Males ASMIX vs ASD$_{DSM}$
Meds Males AS vs ASD$_{DSM}$
Meds Males ASMIX vs ASD$_{DSM}$
NoMeds Males ASMIX vs TD
Density
Alpha values
Mean Alpha value of the small group of the comparison
Median Alpha value of all sub-groups from the large group
Mean Alpha value of each sub-groups from the large group

Figure S39.

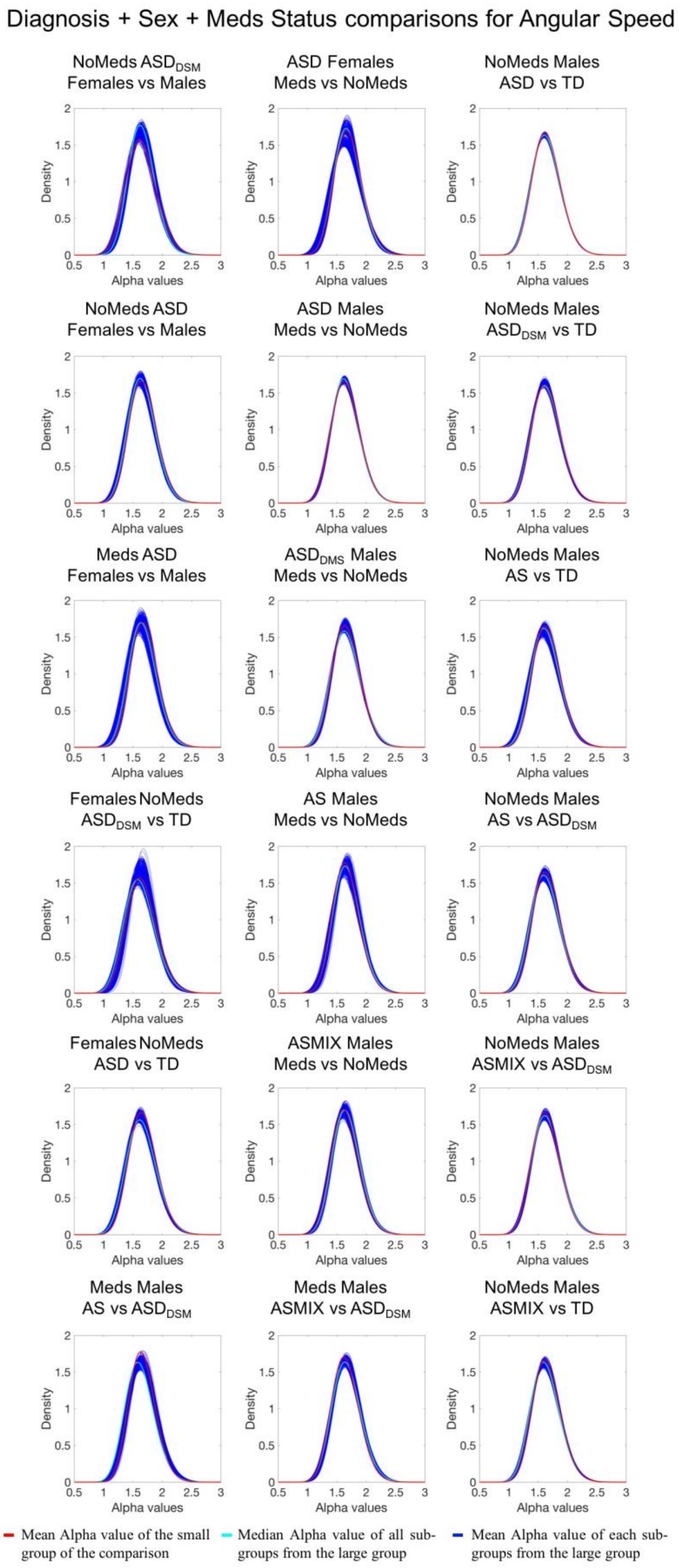
Diagnosis + Sex + Meds Status comparisons for Angular Speed
NoMeds ASD_{DSM} Females vs Males
ASD Females Meds vs NoMeds
NoMeds Males ASD vs TD
NoMeds ASD Females vs Males
ASD Males Meds vs NoMeds
NoMeds Males ASD_{DSM} vs TD
Meds ASD Females vs Males
ASD_{DMS} Males Meds vs NoMeds
NoMeds Males AS vs TD
Females NoMeds ASD_{DSM} vs TD
AS Males Meds vs NoMeds
NoMeds Males AS vs ASD_{DSM}
Females NoMeds ASD vs TD
ASMIX Males Meds vs NoMeds
NoMeds Males ASMIX vs ASD_{DSM}
Meds Males AS vs ASD_{DSM}
Meds Males ASMIX vs ASD_{DSM}
NoMeds Males ASMIX vs TD
Density
Alpha values
Mean Alpha value of the small group of the comparison
Median Alpha value of all sub-groups from the large group
Mean Alpha value of each sub-groups from the large group

Figure S40.

Figures S41-S48 display the comparisons for the estimated Gamma moments (mean and variance) and the scale Gamma value, as the size of the markers. The red dot refers to the small group, blue cluster of 500 dots are from the sub-groups extracted from the large group using the *bootstrapping method* and the cyan dot refers to the median of all the subgroups.

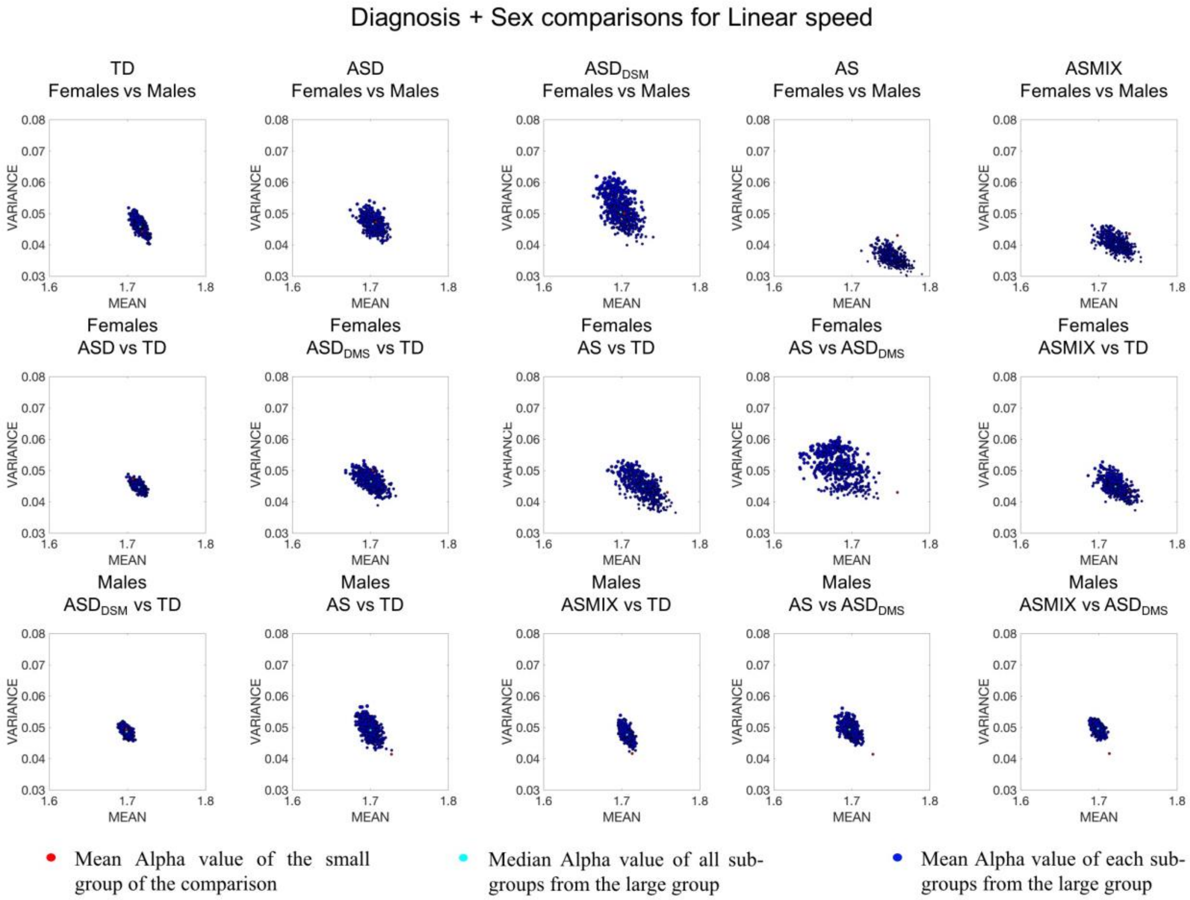

Figure S41.

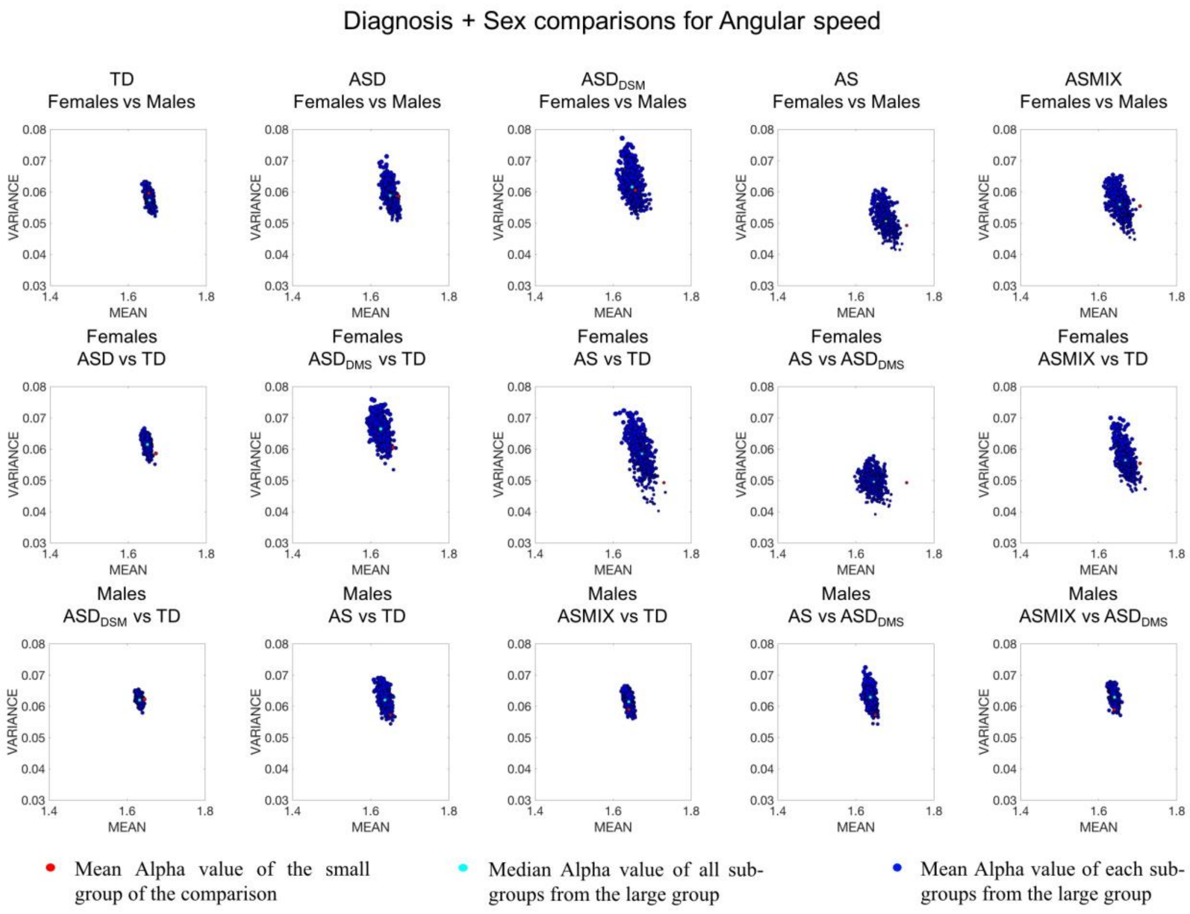

Diagnosis + Sex comparisons for Angular speed
TD Females vs Males
ASD Females vs Males
ASDDSM Females vs Males
AS Females vs Males
ASMIX Females vs Males
Females ASD vs TD
Females ASDDMS vs TD
Females AS vs TD
Females AS vs ASDDMS
Females ASMIX vs TD
Males ASDDSM vs TD
Males AS vs TD
Males ASMIX vs TD
Males AS vs ASDDMS
Males ASMIX vs ASDDMS
VARIANCE
MEAN
Mean Alpha value of the small group of the comparison
Median Alpha value of all sub-groups from the large group
Mean Alpha value of each sub-groups from the large group

Figure S42.

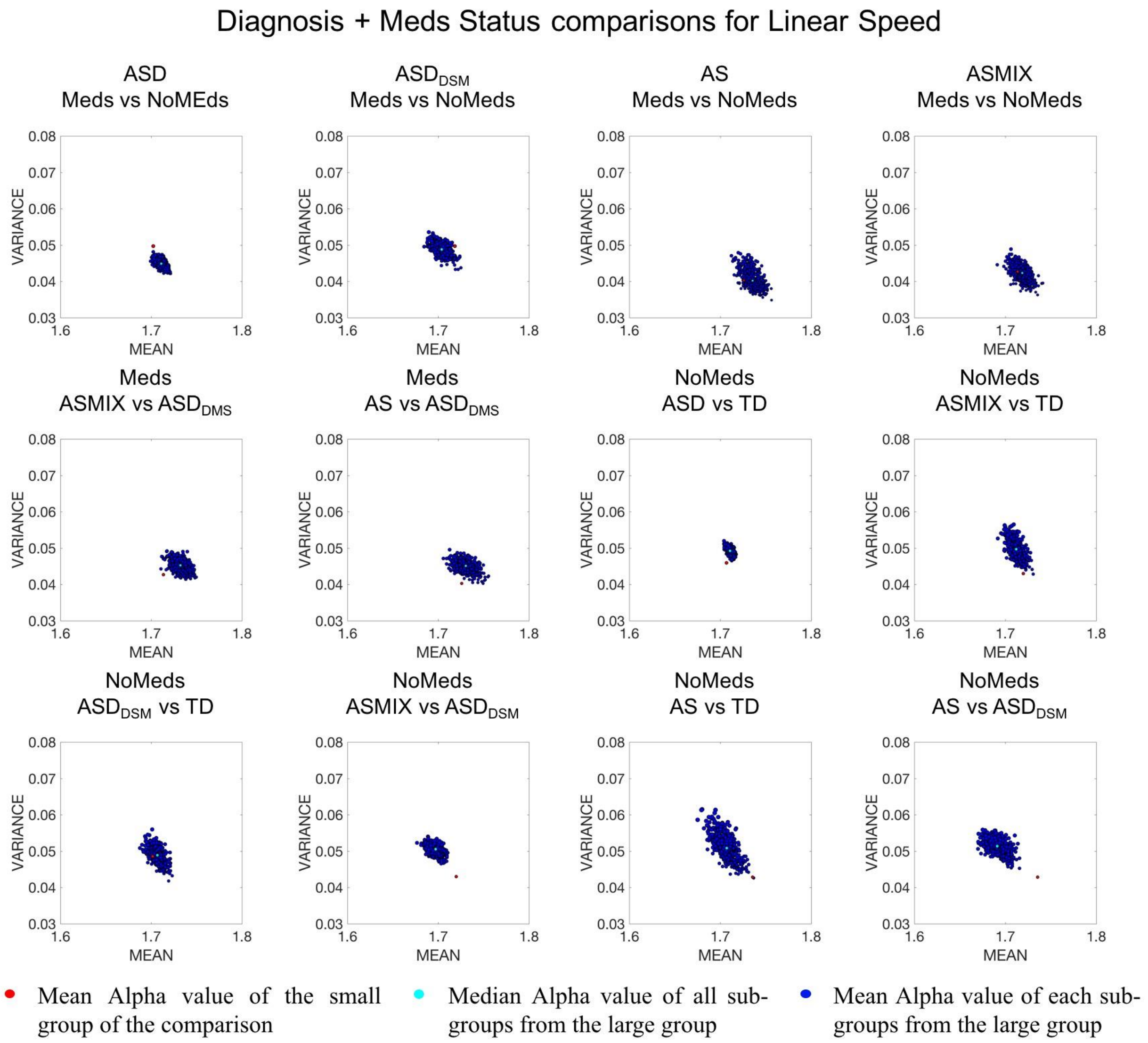
Diagnosis + Meds Status comparisons for Linear Speed
ASD
Meds vs NoMEds
ASD$_{DSM}$
Meds vs NoMeds
AS
Meds vs NoMeds
ASMIX
Meds vs NoMeds
Meds
ASMIX vs ASD$_{DMS}$
Meds
AS vs ASD$_{DMS}$
NoMeds
ASD vs TD
NoMeds
ASMIX vs TD
NoMeds
ASD$_{DSM}$ vs TD
NoMeds
ASMIX vs ASD$_{DSM}$
NoMeds
AS vs TD
NoMeds
AS vs ASD$_{DSM}$
VARIANCE
MEAN
0.03
0.04
0.05
0.06
0.07
0.08
1.6
1.7
1.8
Mean Alpha value of the small group of the comparison
Median Alpha value of all sub-groups from the large group
Mean Alpha value of each sub-groups from the large group

Figure S43.

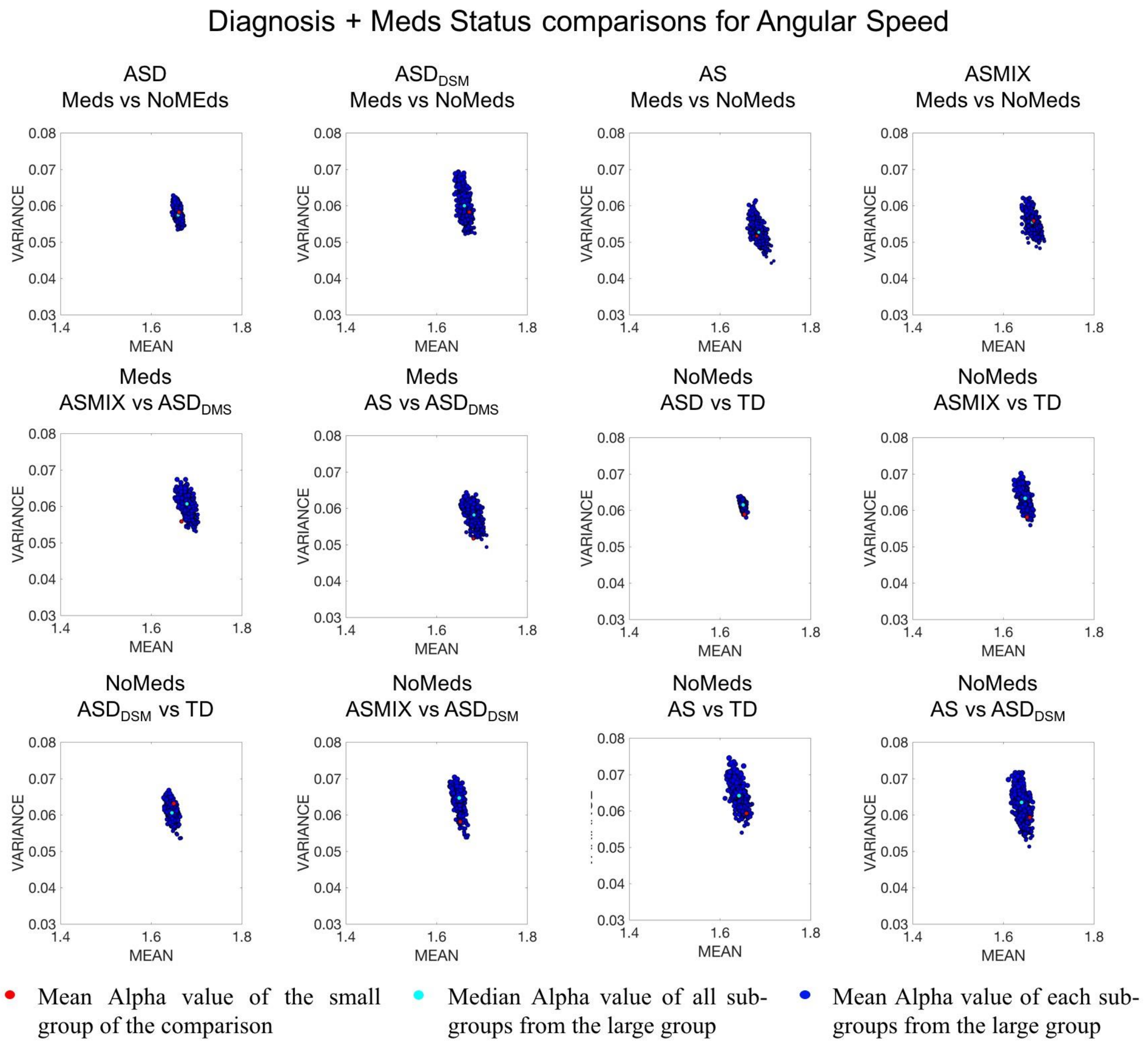
Diagnosis + Meds Status comparisons for Angular Speed
ASD
Meds vs NoMEds
ASDDSM
Meds vs NoMeds
AS
Meds vs NoMeds
ASMIX
Meds vs NoMeds
Meds
ASMIX vs ASDDMS
Meds
AS vs ASDDMS
NoMeds
ASD vs TD
NoMeds
ASMIX vs TD
NoMeds
ASDDSM vs TD
NoMeds
ASMIX vs ASDDSM
NoMeds
AS vs TD
NoMeds
AS vs ASDDSM
VARIANCE
MEAN
0.08
0.07
0.06
0.05
0.04
0.03
1.4
1.6
1.8
Mean Alpha value of the small group of the comparison
Median Alpha value of all sub-groups from the large group
Mean Alpha value of each sub-groups from the large group

Figure S44.

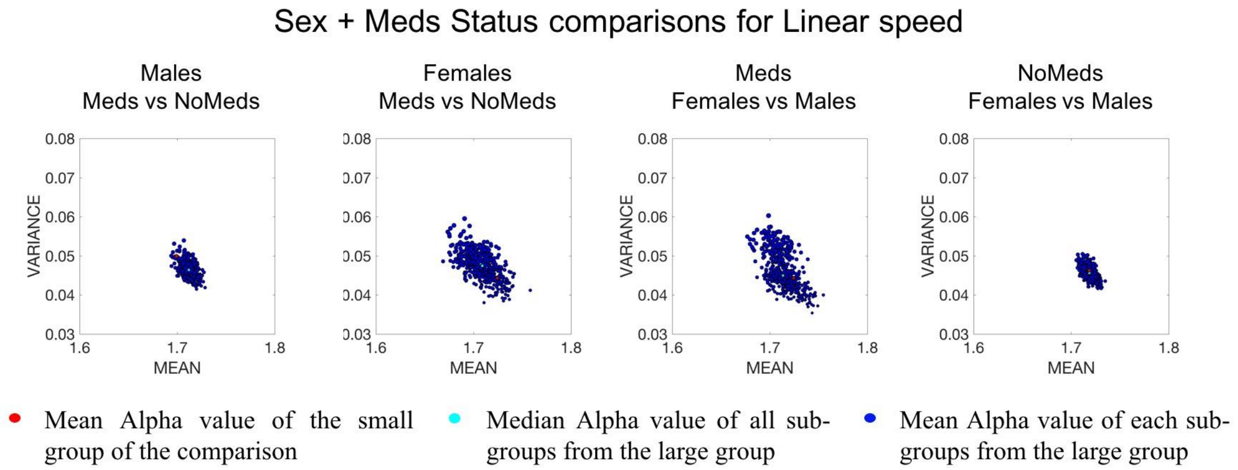

Figure S45.

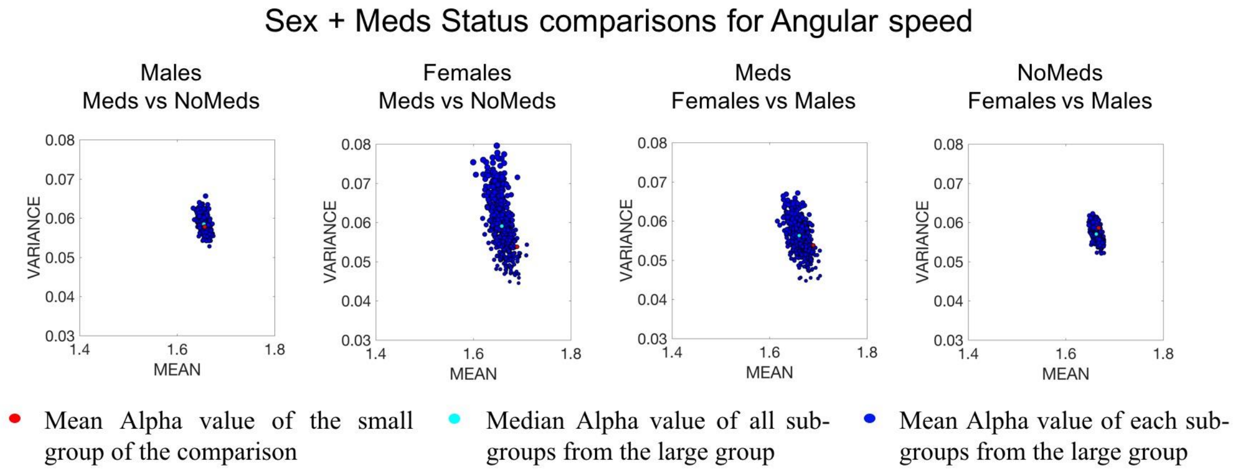

Figure S46.

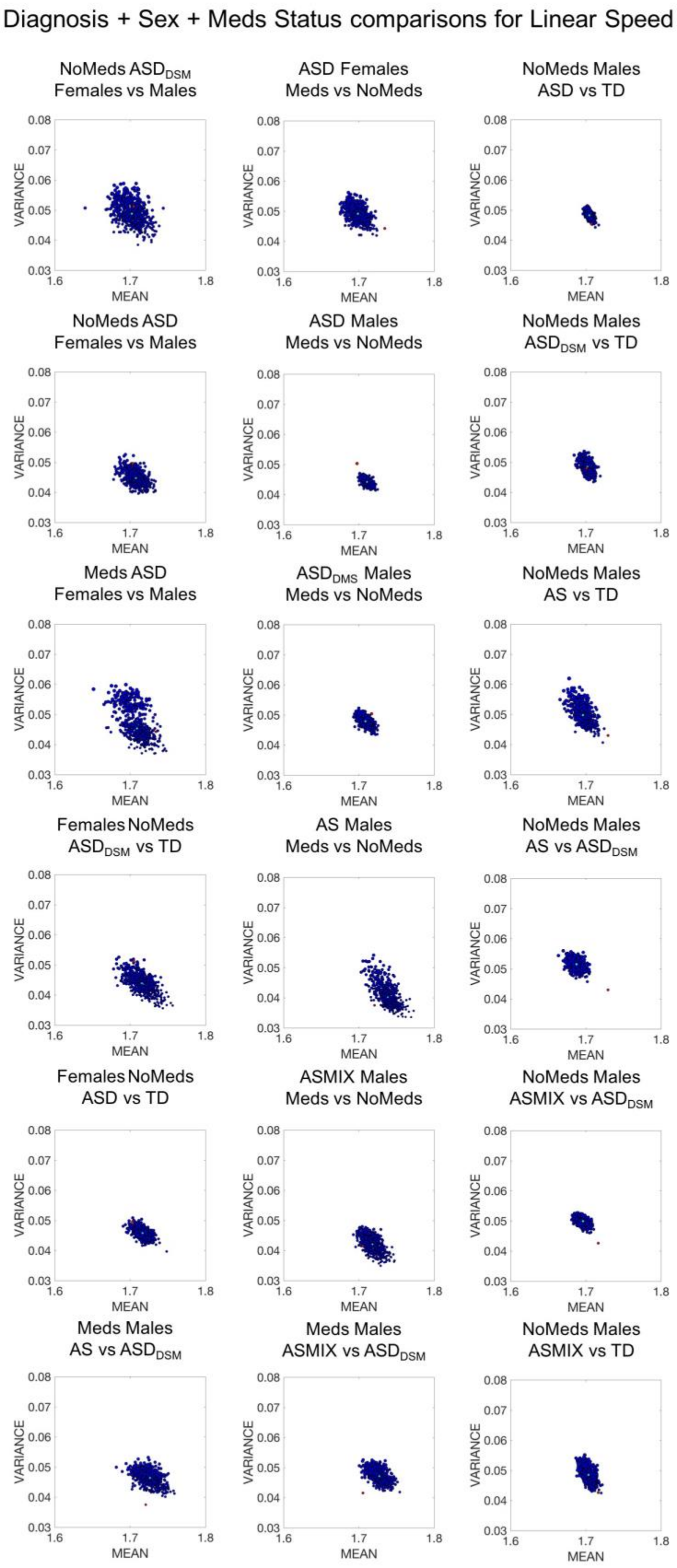

Mean Alpha value of the small group of the comparison

Median Alpha value of all sub-groups from the large group

Mean Alpha value of each sub-groups from the large group

Figure S47.

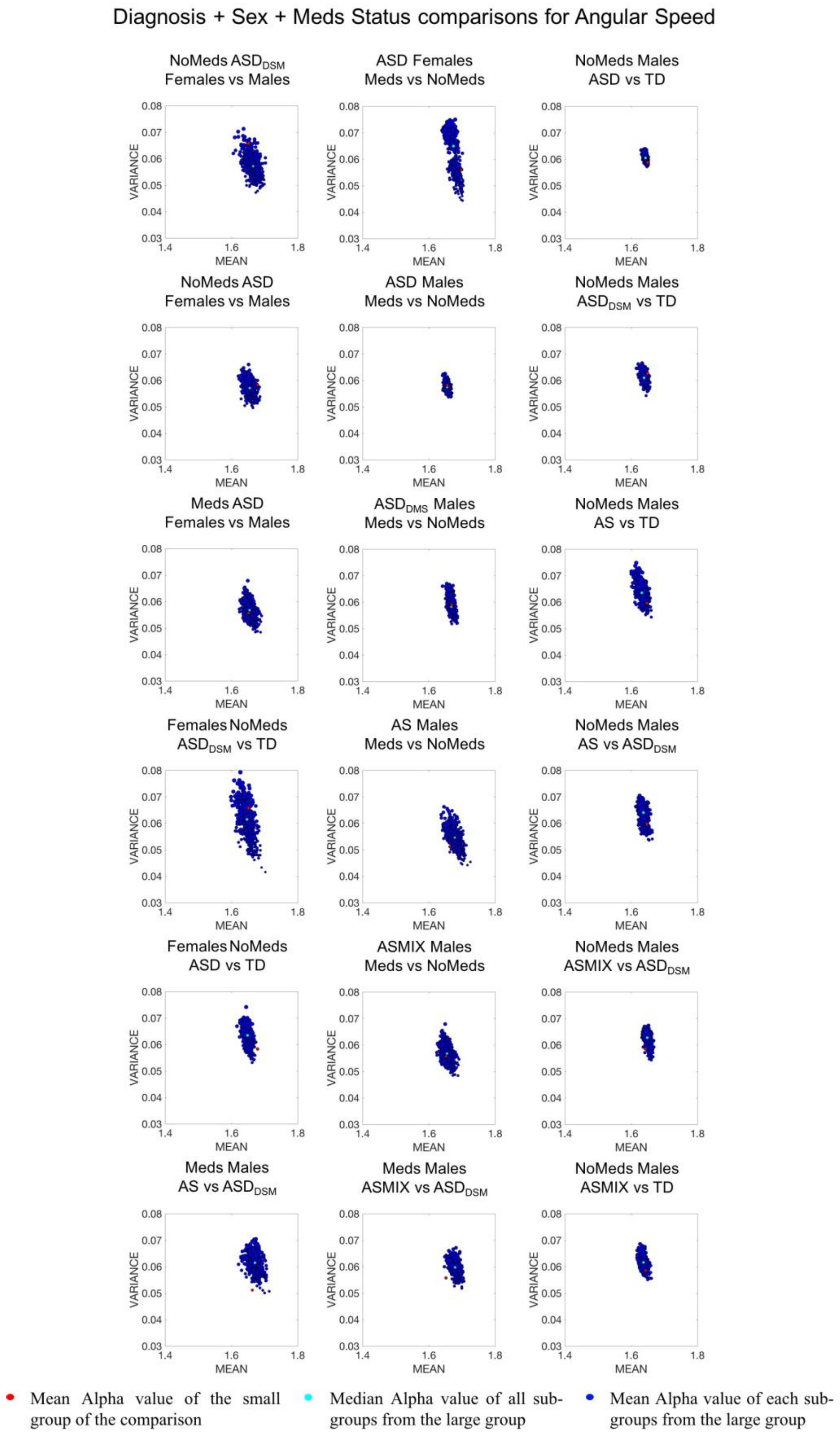
Diagnosis + Sex + Meds Status comparisons for Angular Speed
NoMeds ASD$_{DSM}$ Females vs Males
ASD Females Meds vs NoMeds
NoMeds Males ASD vs TD
NoMeds ASD Females vs Males
ASD Males Meds vs NoMeds
NoMeds Males ASD$_{DSM}$ vs TD
Meds ASD Females vs Males
ASD$_{DMS}$ Males Meds vs NoMeds
NoMeds Males AS vs TD
Females NoMeds ASD$_{DSM}$ vs TD
AS Males Meds vs NoMeds
NoMeds Males AS vs ASD$_{DSM}$
Females NoMeds ASD vs TD
ASMIX Males Meds vs NoMeds
NoMeds Males ASMIX vs ASD$_{DSM}$
Meds Males AS vs ASD$_{DSM}$
Meds Males ASMIX vs ASD$_{DSM}$
NoMeds Males ASMIX vs TD
VARIANCE
MEAN
Mean Alpha value of the small group of the comparison
Median Alpha value of all sub-groups from the large group
Mean Alpha value of each sub-groups from the large group

Figure S48.
