# Supplementary material for: Characterization of Noise Signatures of Involuntary Head Motion in the Autism Brain Imaging Data Exchange Repository
Source: Front Integr Neurosci. 2018 Mar 5;12:7. doi: 10.3389/fnint.2018.00007 (PMC5844956; doi:10.3389/fnint.2018.00007)
Supplement: Supplementary file 2 [file DataSheet2.docx]

# Appendix


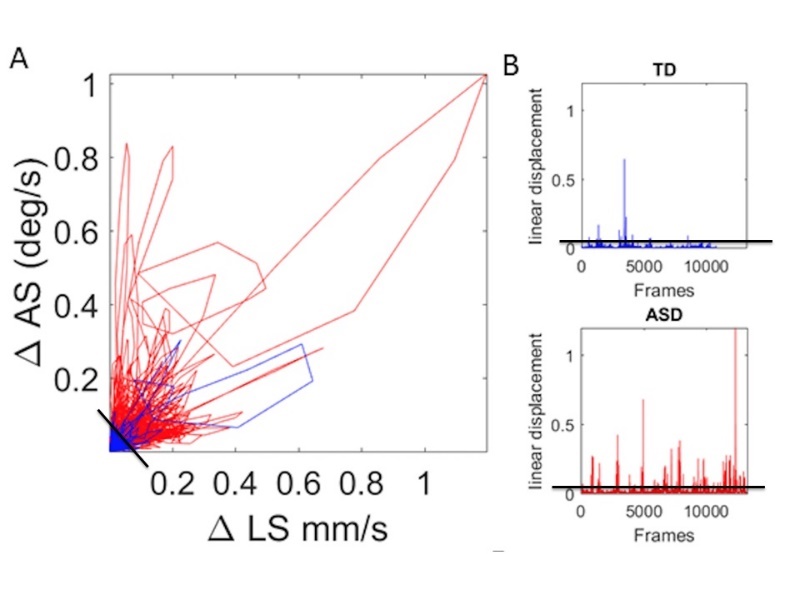


### Appendix Figure A1

**The relevance of performing appropriate statistical analyses of involuntary head motions in resting state fMRI experiments**. (A) Head motion data collated from participants in ABIDE (Figure from previously published work [10]). Parameter plane shows the frame-by-frame cumulative paths along the linear speed change in the horizontal axis (ΔLS) and the angular speed change (ΔAS) in the vertical axis. Head excursions of neurotypical participants (blue trace) cover far shorter paths than those from participants with ASD (red trace). Black line represents a threshold denoting the values corresponding to the speed amplitude in (B). This figure is used to provide a sense for the amount of data wasted in these fMRI work and the notion that (1) the gaps created by the threshold-based scrubbing methods are very different in TD than in ASD; (2) the ASD rate of change is far higher over the course of a session and as such, bound to require more energy than the TD controls; (3) the head-motion criterion for data cleansing in an earlier step of the analytic pipeline will skew the selection of the clean data that further enters in subsequent steps of the pipeline of analyses. As such, head motion data analysis affects the statistical inference procedures and interpretation derived from imaging data. (B) The profiles of the magnitude of the linear displacements (mm/s) of the head in data from (A) profiled frame by frame (one second unit time). Black line denoting a threshold to illustrate the disparity in the data scrubbed from TD control images *vs.* images from participants with ASD.


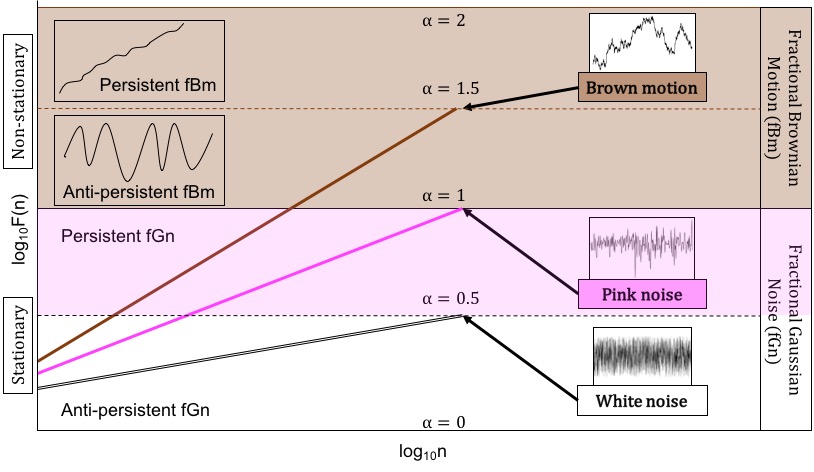


### Appendix Figure A2

**Relationship between α values and types of noise.** Graphic example of different types of noise and their corresponding α value. Fractional Brownian motions (fBm) are a family of processes defined by Mandelbrot and van Ness [35] in which the successive increments in position are correlated, that is, a positive correlation means that an increasing trend in the past is likely to be followed by and increasing trend in the future (persistent series). Conversely, a negative correlation signifies that an increasing trend in the past is likely to be followed by a decreasing trend in the future (anti-persistent series). These families correspond to α exponents ranging from 1 to 2. When α exponent is equal to 1.5, the process is named Brownian motion. It is a stochastic process that, mathematically, is the integral of a white Gaussian noise (α = 0.5). Contrary to fBm, the increments in position of this process are uncorrelated (each displacement is independent to the former, in direction as well as in amplitude). Fractional Gaussian noise is a family of fractal processes, defined as the series of successive increments in fBm. This family corresponds to α exponents ranging from 0 to 1. These two processes are interconvertible but they have different properties: fBm is non-stationary with time-dependent variance, while fGn is stationary and has constant expected mean value and variance over time [36]. Finally, we have a special case that is simply a statistically reliable departure from white noise in the direction of persistence (positive correlated). That case is when α exponent is equal to 1, corresponding to 1/f or pink noise. This noise is characterized by a form of temporal fluctuation that as a power density is inversely proportional to the frequency of the signal. This means that fluctuations at one-time scale are only loosely correlated with those of another time scale, showing a relative independence of the underlying processes acting at different time scales.


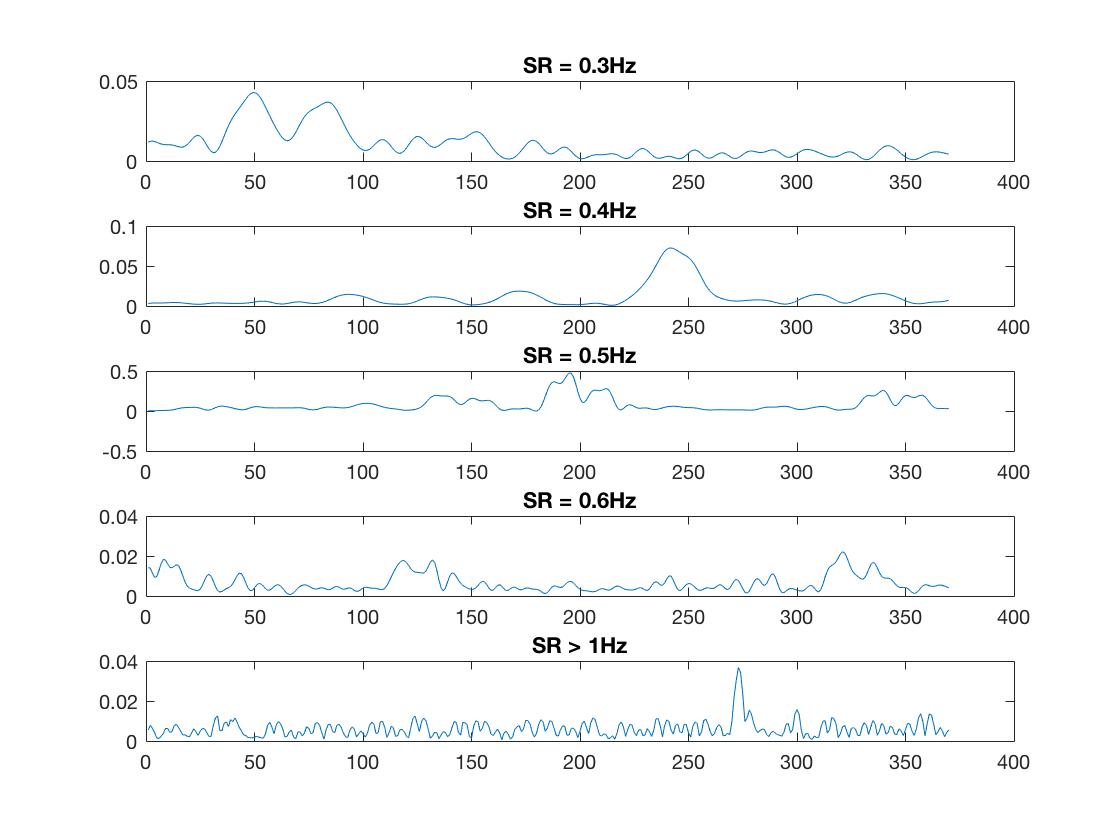


### Appendix Figure A3

**Examples demonstrating visible differences in the involuntary velocity-dependent head motion time series derived from sites with scanners of different sampling resolutions.** All original SR on the top of each plot (0.03, 0.04, 0.05, 0.06 and > 1 Hz). Data were resampled to the same SR=2 Hz while conserving the waveform shape; then time series were truncated to have the same number of points, 370 for consistency and to enable proper DFA calculations (see text).

## Distributional Analyses

One of the problems with traditional approaches involving time series lies in the “*one size fits all*” model employed to analyze head motion kinematics (e.g. position-orientation dependent data and their higher order derivatives). See for example **Appendix Figure 4A(A)** *vs***. (B)** explaining the differences in statistical approaches. Traditional methods a priori assume normality and stationarity in the cumulative distributions of the kinematic parameters extracted from the time series data. As such, theoretical moments (e.g. the Gaussian mean and variance) may be used to describe the variability in the data, without considering the nature of the random process under consideration (i.e. the series of events described by the time series data). As suggested above, these practices incur in gross data loss and likely bias the scrubbing step, ultimately influencing the statistical inference and interpretation of the clean image frames data kept and used in further analyses (**Appendix Figure A4**).

Our recent work involving the head motion data extractable from the ABIDE data sets, has introduced new analytical methods to overcome data loss in kinematic analyses of head motion data. The new methods *empirically* estimate the families of probability distribution functions that best fit normalized variations (micro-movements) in head motion amplitude [9, 10] (**Appendix Figure A4(B)**). They differ fundamentally from conventional methods of head motion analyses (e.g. [12, 37]) as they consider the skewed nature of the kinematics data (e.g. speed dependent data). Indeed, such data fails the range test (**Appendix Figure A4(C)**) -a fact that is not considered by traditional methods taking averages under an assumed theoretical mean, e.g. from a symmetric distribution. In the conventional approach to this step of the pipeline analyses, speed values representing +/- 2 standard deviations from the (assumed) theoretical mean speed may yield negative ranges in positive-scalar data that does not distribute symmetrically.


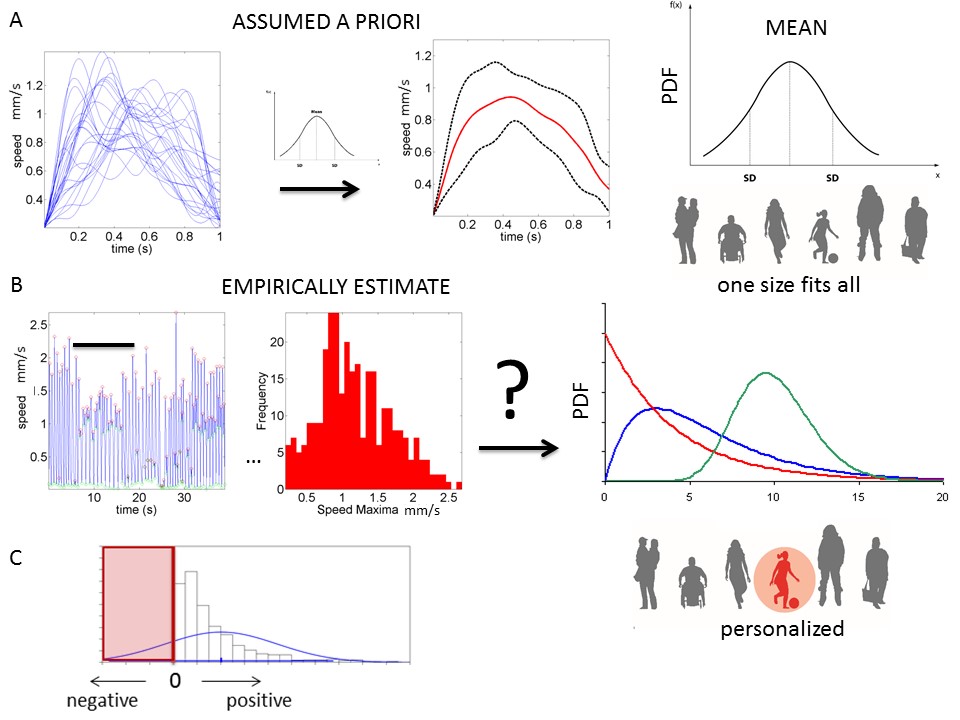


### Appendix Figure A4

**Theoretical assumed *vs.* empirically estimated statistical models avoiding the “*one size fits all*” approach to statistical inference.** (A) Given a time series corresponding to the linear speed, it is possible to build one-second-long epochs and average the speed values under an assumed theoretical mean (e.g. the Gaussian mean) using the same theoretical symmetric probability distribution function (PDF) for everyone. This however loses all the speed information that falls out of the assumed mean +/- the assumed standard deviation. (B) Instead of a priori assuming the PDF, we can empirically estimate it using the continuous data. For example, the same data in (A) spanning a few seconds can be examined by analyzing the fluctuations in peak amplitude (the peaks are marked by red circles and the valleys by green circles), so by isolating the peaks and binning them and building a frequency histogram, we can then use several estimation methods to find which PDF best fits the data with respect to some criteria we may be interested in, according to the investigation at hand. In this way, given the patterns of one person, we can build a personalized statistical estimate of that person’s kinematics. (C) As it turns out the skewed nature of these empirical distributions is such that enforcing a priori a theoretical symmetric distribution and blindly using its theoretical moments to do statistical inference is largely inappropriate, as demonstrated by the range test yielding *negative values* when taking the theoretical assumed mean +/- two times the standard deviation. This applies to the speed or distance based (positive) quantities of interest in the motion data under consideration.

## Bootstrapping to Match Sample Sizes for Statistical Comparison

Given two groups (one small and one large), we used random sampling with replacement and created subgroups drawn from the larger size group matching the size and age composition of the smaller group. We examined the large group’s age value by value, ensuring that the age composition of each subgroup matched that of the small group. For instance, when comparing the TD group (SR0, n = 1,074) with the AS group (SR0, n = 189) there is a disparity in size that we address with bootstrapping. To that end, we designated TD as “the large group” and AS “the small group” (**Appendix Figure A5(A)**). For each group, we built frequency histograms binning the age such that each small-size AS subgroup drawn at random with replacement from the large TD group would have the same age composition as the small group we use for reference (**Appendix Figure A5(B)**). Using the large TD group, we built 500 TD subgroups of size n=189 each and same age composition as the AS small group. Depending on the range of ages within each group, histograms with bin sizes of 0.5 year or 1 year were created for the large and small groups individually, based on of their age distributions (**Appendix Figure A5(B))**. Bin sizes of the small and large groups were overlaid, and the numbers compared to ensure proper age overlap for each subgroup **(Appendix Figure A5(C))**. Once age-matching was completed and all data binned uniformly, corrected sample sizes were obtained. From these sample sizes, the entire dataset from the smaller groups were used **(Appendix Figure A5(D))** to compare to each of the TD-drawn subgroups using the statistical methods described below. This procedure was repeated 500 times, allowing us to obtain 500 randomly chosen sub-samples from original datasets with similar size and age composition as a target group we wished to compare to (**Appendix Figure A5(D)**).

This bootstrapping method permitted us to sample enough from the TD group (500 sub-groups) to exhaust combinations of age and participant number (n = 189 in this case) and then, build a distribution of *p-values* (**Appendix Figure A5(E)**) from the 500 comparisons between each 189-participant drawn from the large TD group and the 189-participant from the small AS group. Similar procedure was performed with all other comparisons as needed, i.e. in the presence of disparate sizes between the two groups of interest.


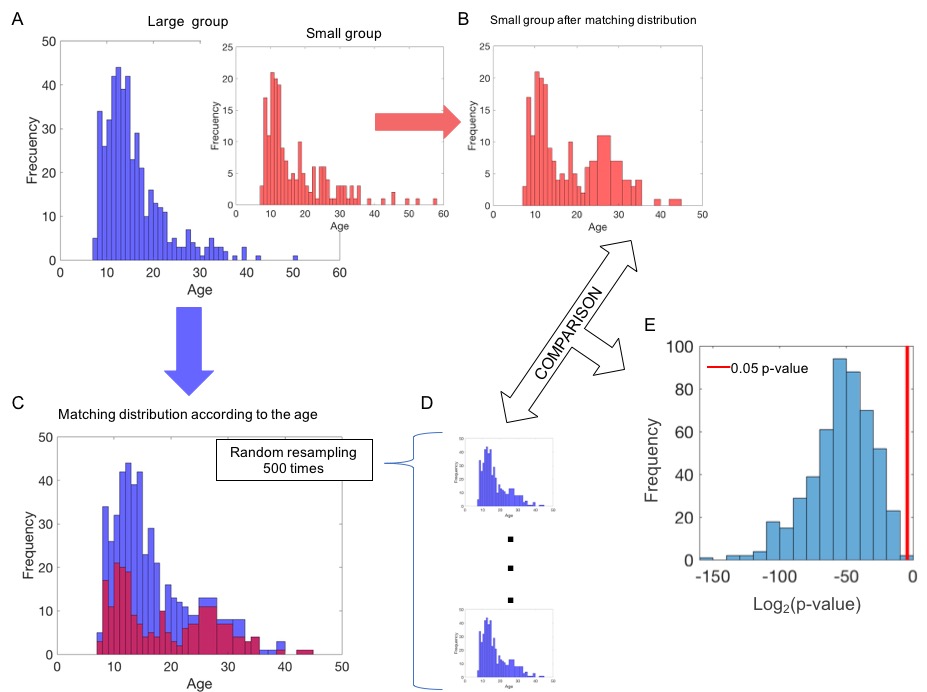


### Appendix Figure A5

**Explanation of the Bootstrapping method.** A) Inconsistent group sizes commonly found in the ABIDE datasets. The group with fewer samples is labeled ‘small group’ (colored in red) in contrast to the group comprised of more samples, coined ‘large group’ (colored in blue). For each group, we built frequency histograms binning the age such that each small-size subgroup drawn at random with replacement from the large group would have the same age composition as the small group we use for reference. B) Age-distribution of the small group after the bin sizes of the small and large groups are overlaid to maximize overlapping in age composition; C) Example of resulting age-matched large and small groups upon overlapping analyses ensuring age-bin composition matching. D) Random sub-samples drawn from the large group while preserving age-bin composition, N = 500 age-matching sub-samples with number of participants matching the small sample size. E) Frequency histogram of log_2_(*p-values)* from the 500 comparisons. The red line represents the cutoff value for significance at 95% confidence.
